# Aetiology of childhood pneumonia in low- and middle-income countries in the era of vaccination: a systematic review

**DOI:** 10.7189/jogh.12.10009

**Published:** 2022-07-23

**Authors:** Claire von Mollendorf, Daria Berger, Amanda Gwee, Trevor Duke, Stephen M Graham, Fiona M Russell, E Kim Mulholland, ARI review group, Trevor Duke, Hamish Graham, Steve Graham, Amy Gray, Amanda Gwee, Claire von Mollendorf, Kim Mulholland, Fiona Russell, Maeve Hume-Nixon, Saniya Kazi, Priya Kevat, Eleanor Neal, Cattram Nguyen, Alicia Quach, Rita Reyburn, Kathleen Ryan, Patrick Walker, Chris Wilkes, Poh Chua, Yasir Bin Nisar, Jonathon Simon, Wilson Were

**Affiliations:** 1 Murdoch Children’s Research Institute, Royal Children’s Hospital, Flemington Road, Parkville, Victoria, Australia; 2 Department of Paediatrics, The University of Melbourne, Parkville, Victoria, Australia; 3 Royal Children’s Hospital, Parkville, Victoria, Australia; 4 Department of Infectious Disease Epidemiology, London School of Hygiene and Tropical Medicine, London, UK

## Abstract

**Background:**

This systematic review aimed to describe common aetiologies of severe and non-severe community acquired pneumonia among children aged 1 month to 9 years in low- and middle-income countries.

**Methods:**

We searched the MEDLINE, EMBASE, and PubMed online databases for studies published from January 2010 to August 30, 2020. We included studies on acute community-acquired pneumonia or acute lower respiratory tract infection with ≥1 year of continuous data collection; clear consistent case definition for pneumonia; >1 specimen type (except empyema studies where only pleural fluid was required); testing for >1 pathogen including both viruses and bacteria. Two researchers reviewed the studies independently. Results were presented as a narrative summary. Quality of evidence was assessed with the Quality Assessment Tool for Quantitative Studies. The study was registered on PROSPERO [CRD42020206830].

**Results:**

We screened 5184 records; 1305 duplicates were removed. The remaining 3879 titles and abstracts were screened. Of these, 557 articles were identified for full-text review, and 55 met the inclusion criteria – 10 case-control studies, three post-mortem studies, 11 surveillance studies, eight cohort studies, five cross-sectional studies, 12 studies with another design and six studies that included patients with pleural effusions or empyema. Studies which described disease by severity showed higher bacterial detection (*Streptococcus pneumoniae*, *Staphylococcus aureus*) in severe vs non-severe cases. The most common virus causing severe disease was respiratory syncytial virus (RSV). Pathogens varied by age, with RSV and adenovirus more common in younger children. Influenza and atypical bacteria were more common in children 5-14 years than younger children. Malnourished and HIV-infected children had higher rates of pneumonia due to bacteria or tuberculosis.

**Conclusions:**

Several viral and bacterial pathogens were identified as important targets for prevention and treatment. Bacterial pathogens remain an important cause of moderate to severe disease, particularly in children with comorbidities despite widespread PCV and Hib vaccination.

Acute lower respiratory infections (ALRI), including pneumonia and viral bronchiolitis, remain among the leading causes of illness and death among children younger than 5 years despite the widespread introduction of pneumococcal conjugate vaccine (PCV) and *Haemophilus influenzae* type b (Hib) vaccine [1]. Several multi-country childhood pneumonia aetiology studies attempted to define the common causes of ALRIs. From 1984 to 1989, the BOSTID (Board on Science and Technology for International Development) Study [2], conducted in 10 countries in Africa, Asia, and Latin America, detected viruses and bacteria from upper respiratory tract specimens and bacterial blood culture and bacterial antigens from urine specimens. The study enrolled children aged <5 years with upper and lower respiratory tract infections with variable case definitions across sites. The study found a high prevalence of respiratory syncytial virus (RSV) (11%-37%) and bacteria (4.5%-40%), predominantly *Streptococcus pneumoniae* and *H. influenzae,* in children with ALRI [2].

The Pneumonia Etiology Research for Child Health (PERCH) study was initiated in 2008 to determine the changing aetiology of childhood ALRI in high burden settings in Africa and Asia [3]. This case-control study included cases consistent with the WHO definition of severe and very severe pneumonia cases, included multiple specimen types and utilised novel analytical methods to analyse microbiological findings. Overall, viruses were found to account for 61.4% of cases, and bacteria for 27.3%. The highest aetiological fraction was attributable to RSV (31%), followed by human metapneumovirus (HMPV) (7.5%), rhinovirus (7.5%), parainfluenza virus (7.4%), *S. pneumoniae* (6.7%), Hib (5.9%) and influenza virus (2.0%). *S. pneumoniae* and *S. aureus* were the most common bacterial causes of severe pneumonia [3]. Another case-control study by the GABRIEL (Global Approach to Biological Research, Infectious diseases and Epidemics in Low-income countries) network was conducted in eight countries, between 2010 and 2014 [4]. The study enrolled children meeting the WHO clinical pneumonia case definition [5] and *S. pneumoniae*, RSV, and rhinovirus were identified as the major causes of pneumonia [4].

This review aimed to determine the common aetiology of severe and non-severe community-acquired pneumonia (CAP) among children 1 month to 9 years of age in low- and middle-income countries (LMICs) globally. This included identifying the main aetiological agents responsible for childhood pneumonia; determining the variation of pneumonia aetiology by region, severity, mortality settings, age groups, comorbidities, and by PCV and Hib vaccine introduction status; and identifying the main pathogens responsible for pneumonia mortality.

## METHODS

### Search strategy and selection criteria

We conducted a systematic review, reported in accordance with PRISMA 2020 guidelines [6], to summarise common aetiological causes of childhood pneumonia in the era of widespread PCV and Hib vaccination. Our protocol was registered with PROSPERO on September 29, 2020 [CRD42020206830]. Studies were identified by searching electronic databases and scanning reference lists of included articles. We searched MEDLINE (Ovid), EMBASE (Ovid), and PubMed, for references from 2010 to date of search (August 30, 2020) in consultation with a research librarian, using Medical Subject Headings (MeSH), thesaurus terms and keywords. The PubMed search used keywords to retrieve E-pubs and items not indexed in MEDLINE. We included terms for pneumonia, different specimen types, different aetiological causes and LMICs. We used “include related terms” options in the searches and combined the search terms using Boolean operators “OR” and “AND”. For the detailed MEDLINE (OVID) and PubMed search strategies see Appendix S1 in the **Online Supplementary Document**.

This review was restricted to articles published from 2010 onwards to focus on the post-PCV and Hib vaccination period and build on a previous review conducted in 2010 [7]. We included studies of acute CAP and ALRI which contained data on children aged from one month to 9 years, had one or more year of continuous data collection, had a clear and consistent case definition for pneumonia (WHO- and non-WHO-defined pneumonia), included the testing of more than one specimen type (except for empyema studies where only pleural fluid was required), had data on more than one pathogen, and included both viruses and bacteria. We limited our search to English language articles from low-and-middle-income countries (LMICs) and included randomised controlled trials, clinical trials, and observational studies (cohort studies, cross-sectional studies, and case-control studies). We excluded retrospective studies that focused on patient subsets; studies that described aetiology of acute bronchiolitis only; studies where we were unable to distinguish the aetiology of pneumonia cases from other syndromes (eg, pneumonia cases within a study of invasive pneumococcal disease (IPD)) or distinguish lower respiratory cases from milder syndromes such as upper respiratory tract infections (URTIs); and studies of hospital-acquired pneumonia patients or ventilator associated pneumonia. Animal studies, case reports, comments, letters, and editorials were also excluded.

### Data extraction, quality assessment and data synthesis

All articles identified during our library database search were extracted into an EndNote library (X7.7.1, New York, USA). All articles were imported into COVIDENCE [8] and duplicates were excluded. Two reviewers screened titles and abstracts of selected citations. Full texts were obtained based on selected citations from screening results. Full text eligibility was performed independently by two reviewers and disagreement was resolved by consensus. Data extraction was performed in COVIDENCE, including: first author, year of publication, country, WHO region and World Bank income classification, mortality setting, PCV and Hib status, study aim, study design and setting, study period, population characteristics, case definition and eligibility determination, specimens collected, laboratory tests and pathogens tested for and identified. The quality and bias of studies were assessed using the “Quality Assessment Tool for Quantitative Studies” developed by the Effective Public Health Practice Project (EPHPP) [9]. This standardised tool results in an overall methodological rating of strong, moderate, or weak in eight areas: selection bias, study design, confounders, blinding, data collection methods, withdrawals and dropouts, intervention integrity and analysis. A narrative synthesis was performed based on identified themes that emerged as the review was conducted. No meta-analysis was conducted.

## RESULTS

**Figure 1:**
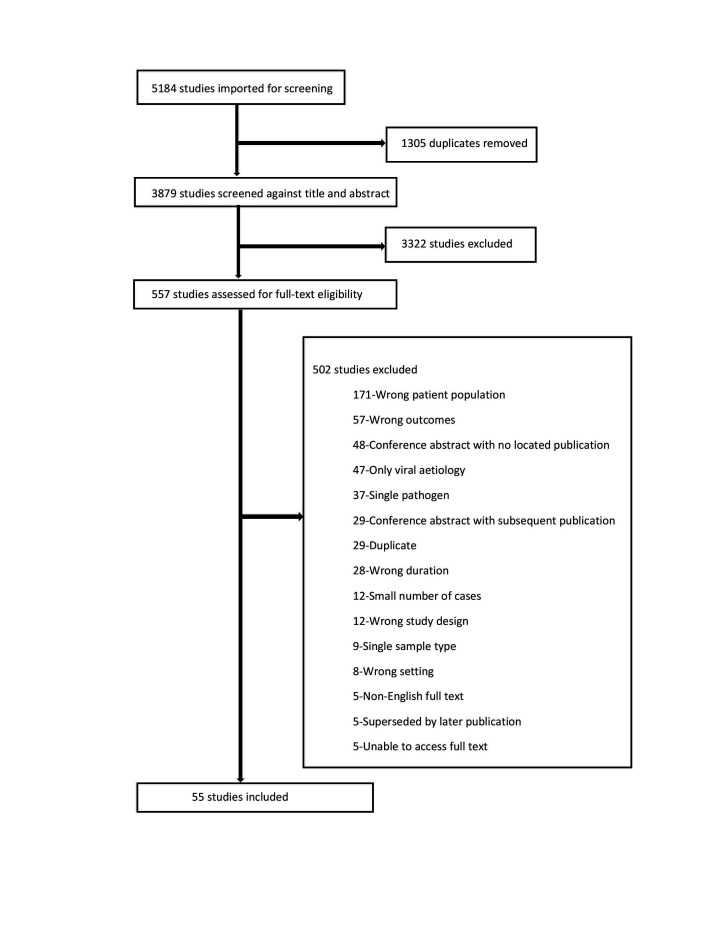
PRISMA flow diagram for search strategy of aetiology of childhood pneumonia review.

Our database search identified 5184 records; 1305 duplicates were removed (**Figure 1**). The remaining 3879 titles and abstracts were screened. Of these, 557 articles were identified for full text review, and 55 met the inclusion criteria. The most common reasons for excluding studies were: wrong patient population (no clear case definition, IPD, only subgroups); wrong outcomes (no aetiology results, no results by age group or diagnosis, only antibiotic resistance or mortality); and conference abstracts with no subsequent publications.

Characteristics are presented separately for each study type (Tables S1-S7 in the **Online Supplementary Document**): 10 case-control studies, three post-mortem studies, 10 surveillance programmes, eight cohort studies, four cross-sectional studies and 12 studies with another study design. We also identified eight studies in patients with pleural effusions or empyema. There were diverse pneumonia case definitions across all studies. Thirty-two studies (25 individual, seven network) included upper-middle income countries, 24 (17 individual, seven network) included lower-middle income countries, and 11 (six individual, five network) included low-income countries (LICs). Out of the 55 studies, 29 (53%) included children aged 5 years or younger only; 18 (33%) included older children, with the upper age limit ranging from 6 to 18 years, predominantly from the Africa Regional Office (AFRO) (n = 8) and for the Western Pacific Regional Office (WPRO) (n = 4) region. The remaining eight (14%) studies included all age groups, with five from the South East Asia Regional Office (SEARO) region and one each from AFRO/WPRO/Pan American health Organization (PAHO). Most studies were conducted in the PCV and Hib vaccination era.

### Case-control studies

Of the 10 case-control studies (**Table 1**; Table S1 in the **Online Supplementary Document**), two were part of the GABRIEL network [4,16] and four part of the PERCH network [3,12,15,17]. The remaining four were conducted in the context of long-standing surveillance programmes or cohort studies [10,11,13,14]. The GABRIEL network [4,16] included children 2-60 months of age with WHO-defined pneumonia hospitalised in eight countries (Cambodia, China, Haiti, India, Madagascar, Mali, Mongolia, Paraguay). Based on upper respiratory sample testing by polymerase chain reaction (PCR), the most common pathogens identified in 888 cases included *S. pneumoniae* (n = 605, 68.2%), *S. aureus* (n = 107, 12.1%), *Hib* (n = 47, 5.3%), HMPV (n = 76, 8.6%), rhinovirus (n = 221, 24.9%), and RSV (n = 178, 20.0%). *S. pneumoniae*, HMPV, rhinovirus, RSV, parainfluenza virus 1, 3, and 4, and influenza virus A and B were independently associated with pneumonia; adjusted population attributable fraction was 42.2% (95% confidence interval (CI) = 35.5%-48.2%) for *S. pneumoniae*, 18.2% (95% CI = 17.4%-19.0%) for RSV, and 11.2% (95% CI = 7.5%-14.7%) for rhinovirus. The mixed bacterial-viral detection rate was 59.6% in cases and 36.1% in controls.

**Table 1:** Aetiology of pneumonia in case-control studies

Lead author and publication date	Country	Specimen types	Diagnostic tests	Findings for LRTI cases			Findings for comparison group	EPHPP Quality Assessment Tool
**AFRO WHO REGION**								
**LOWER-MIDDLE INCOME COUNTRIES**								
				**% (n)**	**Odd ratios**	**By age n (%)**	**% (n)**	
Breiman 2015 [10]	Kenya	Viruses: NPS, OPS	Viruses: RT-PCR	**All** = 28.2% (731/2592)	**INFA** = 2.57 (1.01-6.52), **INFB** = 3.06 (0.41-23.17), **RSV** = 10.15 (3.16-32.58), **Pos≥1 virus** = 2.27 (1.51-3.42), **Pos >2 virus** = 2.36 (1.36-4.11).	**0-11mo: BC = 259: SP** = 4 (1.5%); **SA** = 10 (3.9%); **NTS** = 7 (2.7%); **ST** = 1 (0.4%).	**All** = 4.4% (115/2592)	Weak
						**NPS N = 285: INFA** = 27(9.4%); **INFB** = 4 (1.4%); **RSV** = 74 (25.9%); **AdV** = 57 (20%); **HMPV** = 39(13.7%); **RV/EV** = 32/76 (42%)		
		Bacteria: blood, NPS, OPS	Bacteria: RT-PCR, blood culture	**INFA** = 10.8% (79), **INFB** = 2.6% (19), **RSV** = 21.2% (155), **AdV** = 30.2% (221), **PIV1** = 3.6% (26), **PIV2** = 3.3% (24), **PIV3** = 9.8% (72), **HMPV** = 12.4% (91), **RV/EV** = 47.5% (97), **PAV** = 1.9% (4).		**12-23mo: BC = 236: SP** = 3 (1.3%); **SA** = 1 (0.4%); **NTS** = 3 (1.3%); **ST** = 2 (0.8%).		
						**NPS N = 241: INFA** = 26 (10.8%); **INFB** = 6 (2.5%); **RSV** = 49 (20.3%); **AdV** = 76 (31.5%); **HMPV** 23 = (9.5%); **RV/EV** = 36/73 (49%).	**INFA** = 4.3% (5), **INFB** = 0.9% (1), **RSV** = 2.6% (3), **AdV** 23.5% (27), **PIV1** = 0.9% (1), **PIV2** = 4.3% (5), **PIV3** = 4.3% (5), **HMPV** = 6.1% (7), **RV/EV** = 50% (24), **PAV** = 2.1% (1)	
						**24-59mo: BC = 341: SP** = 1 (0.3%); **SA** = 3 (0.9%); **NTS** = 3 (0.9%); **ST** = 2 (0.6%).		
						**NPS N = 289: INFA** = 39 (13.5%); **INFB** = 11 (3.8%); **RSV** = 46 (15.8%); **AdV** = 108 (36.9%); **HMPV** = 35 (11.9%); **RV/EV** = 29/55 (53%).		
Feikin 2013 [11]	Kenya	Viruses: NPS/ OPS cases + controls	Viruses: qPCR	**For cases in CCS = 199: NPS/OPS** INFA = 18 (9), INFB = 4 (2), INFA/B = 22 (11.1), RSV = 50 (25.1), AdV = 45 (22.6), PIV1 = 4 (2), PIV2 = 12 (6), PIV3 = 20 (10), HMPV = 12 (6), MP = 2 (1.5), RV/EV = 68 (50.4), PAV = 2 (1.5), Pos >1 virus = 113 (84).	**INFA** = 7.2 (0.93-55), **INFB** = 2.0 (0.2-19), **INFA/B** = 4.8 (1.1-21), **RSV** 2.9 (1.3-6.7), **AdV** = 0.89 (0.46-1.8), **PIV1** = 0.60 (0.11-3.3), **PIV2** = 2.6 (0.62-10), **PIV3** = 1.3 (0.49-3.6), **HMPV** = 0.82 (0.28-2.4), **MP** = NC, **RV/EV** = 0.80 (0.41-1.6), **PAV** = 0.30 (0.04-2.3), Pos >1 virus = 1.7 (0.97-2.9).	**<1yo: BC = 172: SP** 1 (0.6%); **NTS** 2 (1.2%).	**For controls in CCS = 93: NPS/OPS** INFA = 1 (1.1), INFB = 1 (1.1), INFA/B 2 (2.2), RSV = 8 (8.6), AdV = 17 (18.3), PIV1 = 3(3.2), PIV2 = 3(3.2), PIV3 = 6 (6.5), HMPV = 6 (6.5), MP = 0 (0), RV/EV = 30 (45.5), PAV = 2 (3.0), Pos >1 virus = 43 (65).	Moderate
						**NPS N = 137: INFA** = 7 (5%); **INFB** = 2 (1.5%); **RSV** = 45 (33%); **AdV** = 18 (13%); **HMPV** = 8 (5.8%); **RV** = 14/40 (35%).		
						**12-23mo: BC = 188: SP** = 3 (1.6%); **HI** = 1 (0.5%); **NTS** = 7 (3.7%).		
						**NPS N = 117: INFA** = 10 (8.5%); **INFB** = 1 (0.9%); **RSV** = 24 (21%); **AdV** = 16 (14%); **HMPV** = 8 (6.8%); **RV** = 25/42 (60%).		
		Bacteria: blood	Bacteria: qPCR, culture	**All: BC** = 735: SP = 5 (0.7), HI = 1 (0.1); **NP/OP** = 408 RSV = 90 (22); AdV = 66 (16); RV/EV = 68/135 (50)		**24-59mo: BC = 375: SP** = 2 (0.5%); **NTS** = 5 (1.3%).		
						**NPS N = 154: INFA** = 10 (6.5%); **INFB** = 2 (1.3%); **RSV** = 21 (14%); **AdV** = 32 (21%); **HMPV** = 5 (3.2%); **RV** = 29/53 (55%).		
Hammitt 2012 [12]	Kenya	Viruses: NPS, OPS, IS, Serum.	Viruses: Serology, PCR.	All cases (N = 805): RSVA = 136 (16.9), RSVB = 77 (9.6), AdV = 39 (4.8), RV = 184 (22.9), PIV1 = 9 (1.1), PIV2 = 5 (0.6), PIV3 = 47 (5.8), PIV4 = 11 (1.4), INFA = 7 (0.9), INFB = 2 (0.3), INFC = 3 (0.4), HMPV = 25 (3.1), MP = 3 (0.4).	**RSVA** = **3.8 (2.2–6.6)**, **RSVB** = **11.9 (3.7–38.2)**, **AdV** = 0.7 (0.4–1.2), **RV** = 1.0 (0.7-1.3), **PIV1** = 0.9 (0.3–2.7), **PIV2** = **0.3 (0.1-0.8)**, **PIV3** = 0.9 (0.5-1.6), **PIV4** = 1.4 (0.4-4.5), **INFA** = 0.7 (0.2-2.2), **INFC** = 0.8 (0.1-4.8), **HMPV** = 2.8 (0.9-8.1), **MP** = 0.5 (0.1-2.1).	**No details by age:** Cases with all samples (n = 257): 24 (9) bacteria, 137 (53) viruses, 39 (15) mixed; Considering CCS: 58 (23) bacteria, 33 (13) viral, 5(2) mixed.	All controls (N = 369): RSVA = 16 (4.3), RSVB = 3 (0.8), AdV = 28 (7.6), RV = 82 (22.2), PIV1 = 5 (1.4), PIV2 = 8 (2.2), PIV3 = 22 (6.0), PIV4 = 4 (1.1), INFA = 5 (1.4), INFB = 0 (0.0), INFC = 2 (0.5), HMPV = 4 (1.1), MP = 4 (1.1).	Strong
		Bacteria: NPS, OPS, IS, blood, serum	Bacteria: Serology, PCR, culture.					
**UPPER-MIDDLE INCOME COUNTRIES**								
Zar 2016 [13]	South Africa	Viruses: NPS, IS.	Viruses: qRTPCR FTDResp33.	**Viruses:** RSV = 66(23%), INF = 32 (11%), PIV = 35 (12%), AdV = 53 (19%), HMPV = 29 (10%), BV = 37 (13%), CMV = 151 (53%), CoV = 33 (12%), EV = 37 (13%), RV = 100 (35%).	**Viruses:** OR (95%CI): RSV = 8.05 (4.21-15.38), INF = 4.13 (2.06-8.26), PIV = 2.03 (1.20-3.42), AdV = 2.15 (1.31-3.53), HMPV = 1.12 (0.67-1.88), BV = 2.29 (1.25-4.17), CMV = 1.57 (1.11-2.21), CoV = 1.20 (0.75-1.97), EV = 0.93 (0.58-1.49), RV = 0.87 (0.63-1.20).		**Viruses:** RSV = 17 (4%), INF = 11 (3%), PIV = 26 (6%), AdV = 41 (10%), HMPV = 44 (11%), BV = 32 (8%), CMV = 177 (43%), CoV = 43 (10%), EV = 57 (14%), RV = 161 (39%).	Strong
		Bacteria:NPS, IS, Blood.	Bacteria qRTPCR FTDResp33; Blood culture	**Bacteria**: BP = 6 (2%), Hib = 4 (1%), MP = 10 (4%), SA = 81 (28%), HI = 152 (54%), SP = 168 (60%), MC = 214 (75%). **Fungi:** PJP = 44 (16%).	**Bacteria:** OR (95%CI): BP = 11.08 (1.33-92.54), Hib = 1.08 (0.28-4.10), MP = 1.20 (0.54-2.78), SA = 0.70 (0.48-1.02), HI = 1.67 (1.20-2.30), SP = 1.07 (0.76-1.48), MC = 1.19 (0.82-1.74). **Fungi:** PJP = 0.35 (0.22-0.55)		**Bacteria:** BP = 1 (0%), Hib 5 = (1%), MP = 14 (3%), SA = 142 (35%), HI = 164 (40%), SP = 237 (58%), MC = 292 (71%). **Fungi:** PJP = 122 (30%).	
**SEARO WHO REGION**								
**LOWER-MIDDLE INCOME COUNTRIES**								
Chowdhury 2020 [14]	Bangladesh	Viruses: NPW.	Viruses: rRT-PCR.	**Virus + ve** = 69.9% (251/359): RV = 22% (79), RSV = 8.9% (32), AdV = 6.4% (23), PIV3 = 5% (18), HMPV = 4.5% (16), INFA = 3.6% (13), INFB = 0.8% (3), PIV1 = 0.8% (3), PIV2 = 0.3% (1). Multiple viruses = 17.5% (63).	**RSV** = **13.1 (1.6-106.1)**	**Inpatient death:** RSV = 0%; AdV = 4% (1/23); INF = 6% (1/16); RV = 5% (4/79); PIV = 14% (3/22); HMPV = 13% (2/16).	**Virus + ve** = 44.8% (148/330): RV = 24.8% (82), AdV = 7.9% (26), HMPV = 1.5% (5), RSV = 0.9% (3), HPIV3 = 0.9% (3), INFA virus = 0.3% (1), HPIV1 = 0.3% (1), INFB virus = 0.3% (1). Multiple viruses = 7.9% (26).	Moderate
					**AdV** = 1.4 (0.6-3.5)			
					**INF** = 8.7 (1.0-78.9)			
		Bacteria: blood.	Bacteria: Culture.	**Bacteria BC +ve** = 4% (16): PA = 25% (4), Enterococcus = 12.5% (2), ST = 12.5%(2), SP = 6.3% (1), SA = 6.3% (1), KP = 6.3% (1).	**RV** = 0.7 (0.4-1.4)	**Post discharge death:** RSV = 0%; AdV = 4% (1/23); INF = 6% (1/16); RV = 3% (2/79); PIV = 5% (1/22); HMPV = 0%.		
					**PIV** = 3.8 (1.0-14.8)			
					**HMPV** = **2.7 (1.3-5.5)**			
**UPPER-MIDDLE INCOME COUNTRIES**								
Piralam 2020 [15]	Thailand	Viruses: NPS/ OPS.	Viruses: qRTPCR assay (FTD Resp33).	**SP NPS/OPS:** PCR Positive = 121 (54.5%), Culture positive = 89 (40.1%), PCR or culture = 127 (57.2%); **SP whole blood**: PCR positive 3 (1.4%). No cases were positive for SP by blood culture. Pneumococcal density was not increased in mixed viral infections with RSV or INF.			**SP NPS/OPS:** PCR Positive = 406 (62.5%), Culture positive = 340 (52.4%), PCR or culture = 417 (64.2%); **SP whole blood**: PCR positive = 5 (0.8%)	Strong
		Bacteria NPS/ OPS, Blood.	Bacteria qRTPCR assay, Culture.					
**MIXED WHO REGIONS AND INCOME CLASSIFICATIONS**								
**GABRIEL NETWORK**								
Benet 2017 [16]	India Madagascar Mali Paraguay	Viruses: NS, NPA, blood, PF, urine.	Both viruses & bacteria: RT-PCR	**Hypoxaemic pneumonia Respiratory samples = 70:** SP = 63.9% (44); SA = 17.4% (12); Hib = 5.7% (4); HMPV = 14.5% (10); AdV = 5.7% (4); RSV = 25.7% (18); PIV1 = 4.3% (3); PIV2 = 1.4% (1); PIV3 = 1.4% (1); PIV4 = 2.9% (2); INFA = 5.7% (4); **Blood samples:** SP = 14.3% (10); SA = 4.3% (3); Hib = 4.3% (3)	Significant aOR: **HMPV** = 2.4 (1.0-5.8); **RSV** = 2.5 (1.1-5.3)	**Findings associated with death**: **SP PCR pos** 5/13 (38.5%) HR = 4.6 (1.5-14.0); **PIV2 pos** 1/13 (7.7%) HR = 23.6 (3.0-183.9)	**Non-hypoxaemic respiratory samples = 335:** SP = 60.3% (202); SA = 17.3% (58); Hib = 5.1% (17); MP = 0.9% (3); HMPV = 6.9% (23); AdV = 7.8% (23); RSV = 13.1% (44); PIV1 = 3.9% (13); PIV2 = 0.3% (1); PIV3 = 6.3% (21); PIV4 = 3% (10); INFA = 7.2% (24); **Blood samples:** SP 12.2% (41); SA = 1.5% (5); Hib = 4.5% (15)	Weak
		Bacteria: blood, fluid, respiratory specimens.						
Benet 2017 [4]	Cambodia China Mongolia India Madagascar Mali Paraguay Haiti	Both viruses & bacteria: NPS, urine, blood, PF	Viruses: RT-PCR.	**Cases = 888:** SP = 605 (68.2%); SA = 107 (12.1%); HI = 47 (5.3%); MP = 13 (1.5%); HMPV = 76 (8.6%); EV = 42 (4.7%); RV = 221 (24.9%); RSV = 178 (20.0%); PIV1 = 26 (2.9%); PIV2 = 4 (0.4%); PIV3 = 57 (6.4%); PIV4 = 21 (2.4%); INFA = 59 (6.6%); INFB = 26 (2.9%)	**SP** = 2.6 (2.0-3.3); **MP** = 9.2 (2.5-33.5); **HMPV** = 11.0 (5.4-22.3); **RV** = 1.8 (1.4-2.4); **RSV** = 11.7 (7.4-18.5); **PIV1** = 7.5 (2.9-19.7); **PIV3** = 6.7 (3.6-12.6); **PIV4** = 2.6 (1.1-6.0); **INFA** = 55.2 (7.4-411.3); **INFB** = 3.3 (1.5-7.3)	**Population attributable fraction by age:**	**Controls = 870;** SP = 412 (47.5%); SA = 148 (17.0%); HI = 57 (6.6%); MP = 6 (0.7%); HMPV = 10 (1.1%); EV = 38 (4.4%); RV = 188 (21.6%); RSV = 34 (3.9%); PIV1 = 9 (1.0%); PIV2 = 5 (0.6%); PIV3 = 18 (2.1%); PIV4 = 12 (1.5%); INFA = 4 (0.5%); INFB = 11 (1.3%)	Moderate
						**2–11 mo: SP** = 43.5 (33.6-51.9); **RSV** = 24.6 (23.5-25.7) **; HMPV** = 6.4 (5.1-7.7).		
						**12–23 mo: SP** = 44.4 (28.4-56.8); **RSV** = 16.6 (15.2-18.0); **HMPV** = 9.9 (8.8-10.9).		
			Bacteria Culture, RT-PCR.			**24–60 mo:** SP = 41.6 (30.6-50.9); RSV = 11.0 (8.6-13.3); HMPV = 7.1 (6.2-8.1).		
**PERCH NETWORK**								
				**Aetiological fraction for all**	**Aetiological fraction by age/severity**			
O'Brien 2019 [3]	The Gambia Zambia South Africa Kenya Bangladesh Thailand Mali	Viruses: NPS/ OPS.	Viruses: FTD Resp33 multiplex qPCR; NPS/ OPS culture.	Viruses = 61.4% of causes, whereas bacteria accounted for = 27.3% and Mycobacterium tuberculosis for 5.9%.	**AF<1yo:** RSV = 39.7% (36.3-43.5), SP = 4.7% (3.2-6.6), HMPV = 8.3% (6.5-10.7).		See Aetiological fraction	Strong
					**AF> = 1yo:** RSV = 16.5% (13.5-19.8), RV = 15.4% (10.6-21.0), SP = 10.1% (7.4-13.6).			
		Bacteria: Blood, NPS/ OPS, IS, lung aspirate, PF, GA.	Bacteria: BC/PCR; NPS/ OPS culture/PCR; IS culture; Lung aspirate culture/PCR; PF culture/PCR; GA culture.	**AF for all ages and cases**: RSV = 31.1% (28.4-34.2), RV = 7.5% (5.3-10.1), HMPV = 7.5% (5.9-9.5), PIV = 7.4% (5.8-9.3), INF = 2.0% (1.1-3.2), HI = 5.9% (3.8-8.5), SP = 6.7% (5.1-8.5), TB = 5.9% (3.9-8.3), SA = 2.7% (1.5-4.3), PJP = 2.0% (0.9-3.3).	**AF severe pneumonia**: RSV = 35.2% (31.7-39.6), RV = 8.1% (5.4-11.1), HMPV = 8.2% (6.5-10.6), SP 4.6% (3.2-6.2).			
					**AF very severe pneumonia:** RSV = 25.2% (22.0-29.1), HI = 7.9% (4.0-12.7), HMPV = 7.8% (5.2-11.0), SP 9.7% (6.9-13.1).			
Thea 2017 [17]	The Gambia Zambia South Africa Kenya Bangladesh Thailand Mali	Viruses: IS, NPS.	Viruses and bacteria: qRTPCR	**Radiological pneumonia - n(%)**: HI = 600 (53.5); Hib = 22 (2.0); MC = 672 (59.9); PJP = 94 (8.4); SA = 140 (12.5); SP = 795 (70.9); AdV = 149 (13.2); CMV = 572 (50.8); HMPV = 133 (11.9); INFA = 39 (3.5); INFB = 15 (1.3); PIV1 = 84 (7.5); PIV2 = 19 (1.7); PIV3 = 75 (6.7); PIV4 = 27 (2.4); RV = 243 (21.7); RSV = 279 (24.8)	**Odds ratio adjusted for NP/OP:** HI = 1.04 (0.75-1.45), Hib = 1.07 (0.36-3.16), MC = 0.87 (0.62-1.24), PJP = 1.03 (0.54-1.98), SA = 0.87 (0.55-1.40), SP = 0.98 (0.66-1.44), AdV = 0.74 (0.47-1.17), CMV = 0.69 (.50-.95), HMPV = 0.71 (.42-1.21), INFA = 0.48 (.17-1.38), INFB = 2.13 (0.23-20.0), PIV1 = 2.17 (0.96-4.91), PIV2 = 2.74 (0.73-10.31), PIV3 = 1.18 (0.53-2.60), PIV4 = 0.52 (0.17-1.57), RV = 0.78 (0.54-1.12), RSV = 1.08 (0.61-1.89)		**Non-pneum**: HI = 168 (43.2); Hib = 8 (2.1); MC 258 (66.3); PJP = 20 (5.1); SA = 46 (11.8); SP = 279 (71.7); AdV = 58 (14.8); CMV = 204 (52.2); HMPV = 41 (10.5); INFA = 19 (4.9); INFB = 11 (2.8); PIV1 = 18 (4.6); PIV2 = 4 (1.0); PIV3 = 21 (5.4); PIV4 = 10 (2.6); RV = 92 (23.7); RSV = 60 (15.3)	Strong
		Bacteria: IS, NPS						

*SP – Streptococcus pneumoniae, SA – Staphylococcus aureus, MC – Moraxella catarrhalis, BP – Bordetella pertussis, Hib – Haemophilus influenzae* type b, *MP – Mycoplasma pneumoniae, HI – Haemophilus influenza, NTS – Non-typhi Salmonella, KP – Klebsiella pneumoniae, CP – Chlamydophila pneumoniae, PA – Pseudomonas aeruginosa, ST – Salmonella typhi,* RV – Rhinovirus, EV – Enterovirus, RSV – Respiratory syncytial virus, INFA/B/C – Influenza (types A, B, and C), PIV1/2/3/4 – Parainfluenza (types 1, 2, 3, and 4), AdV – Adenovirus, HMPV – Metapneumovirus, BV – Bocavirus, CMV – Cytomegalovirus, CoV – Coronavirus (NL63,229E, OC43, and HKU1), PAV – Parechovirus, PJP – *Pneumocystis jirovecii*, NPW – Nasopharyngeal wash, NPS – Nasopharyngeal swab, PF – pleural fluid, OPS – oropharyngeal swab, IS – induced sputum, GA – gastric aspirate, BC – blood culture; y – year, yo – year old, mo – months

The PERCH network [3,12,15,17] included children 1-59 months of age with WHO-defined (2005) severe and very severe pneumonia [5] hospitalised in seven countries (Bangladesh, The Gambia, Kenya, Mali, South Africa, Thailand, Zambia). All countries had introduced Hib, except Thailand, and PCV, except Thailand, Bangladesh and Zambia (the latter introduced PCV in the last few months of the study). Based on an integrated aetiological analysis incorporating multiple specimens (including oro/nasopharyngeal swabs) and tests, viruses accounted for 61.4% of causes, bacteria for 27.3% and *Mycobacterium tuberculosis* for 5.9%. This varied across age groups and pneumonia severity, with viruses less common (54.5% vs 68.0%) and bacteria more common (33.7% vs 22.8%) in very severe compared with severe pneumonia cases. Results also varied according to specimen type and test used. Around 3% of blood cultures and 13.5% of lung aspirate cultures across all sites tested positive for bacteria. For all age groups and cases, RSV had the highest aetiological fraction, 31.1% (95% CI = 28.4-34.2). Mixed bacterial-viral detection was high in both cases (83.5%) and controls (75.8%) [3].

In the four case-control studies not part of networks [10,11,13,14], only RSV and influenza were consistently shown to be more commonly detected in cases than controls. In two of these studies, the control group were children visiting clinics for non-severe illness, immunisations or medicine refills, with no history of fever, respiratory symptoms or diarrhoea during the preceding two weeks [10,11]; one study included children with no pneumonia on admission, and no recent history of respiratory symptoms [14], while the last study included controls who were asymptomatic or had URTI symptoms [13]. Severe acute respiratory illness (SARI) surveillance in Kenya reported that the frequency of viruses differed by age, with RSV more common in the 0-11-month age group and influenza and adenovirus more common in the 24-59-month age group [10,11]. Rhinovirus was common across all age groups.

Only PERCH described ALRI aetiology by severity of disease [3], with a higher proportion of bacteria (*S. pneumoniae* and *H. influenzae*) observed in very severe (cough or difficulty breathing and one or more danger signs) compared to severe pneumonia (cough or difficulty breathing with lower chest wall indrawing). Two other studies described deaths in pneumonia patients diagnosed with viral and bacterial aetiology – HMPV (in severely malnourished children) [14], parainfluenza virus and *S. pneumoniae* [16] were found to be important in these cases. In the latter study, three of the four sites introduced PCV during the course of the study [16]. For children with pneumonia and comorbidities, the PERCH study found malnutrition was more common in cases than controls. Among cases, those who had *Pneumocystis jirovecii* detected on nasopharyngeal swab were more likely than other cases to be <6 months of age and malnourished [3].

The only high mortality settings (under 5 mortality rate >50 deaths per 1000 live births) were LICs/LMICs that were part of the two network studies. In PERCH, the three high mortality sites in Africa (The Gambia, Mali and Zambia) reported RSV as the most common pathogen in HIV-uninfected CXR positive patients, with parainfluenza, *S. pneumoniae* or HMPV rated second respectively. RSV was also the most common pathogen in the lower mortality sites. In the GABRIEL Network, Haiti, Mali and Madagascar had high mortality. *S. pneumoniae* was the main bacterium associated with pneumonia in these countries as well as the lower mortality countries.

In the PERCH study, bacterial and virus proportions varied by WHO region [3]. AFRO countries showed a higher proportion of bacterial pathogens, while SEARO countries detected proportionally more viruses; likely partially due to differences in the presentation of enrolled cases, with proportionally more cases in Bangladesh presenting with wheezing [3]. In the GABRIEL network study, *S. pneumoniae* was high in the AFRO, PAHO, and in one WPRO site; the exceptions were China, Cambodia and India (Lucknow), where viruses were detected more commonly [4]. Of the 10 case-control studies, five were deemed of high quality, three moderate and two weak as rated by the EPHPP Quality Assessment Tool.

### Post-mortem studies

Of the three post-mortem studies (**Table 2**; Table S2 in the **Online Supplementary Document**) [18-20], two were part of the CHAMPS (Child Health and Mortality Prevention Surveillance) study which included sites in the WHO SEARO (Bangladesh) and AFRO regions (Mali, Mozambique, South Africa, Kenya) [19,20]. These studies showed that CAP was responsible for 25.2% (in children 0-15 years) to 47% (in children <60 months) of deaths. The most common pathogens identified in children 1-59 months of age who died of lower respiratory tract infections were nosocomial and community-acquired *Klebsiella pneumoniae* (15.6%-17.8%), cytomegalovirus (CMV, 7%-15.6%), *S. pneumoniae* (12.5%-15.1%), RSV (5.5%-21.9%) and *P. jirovecii* (9%-18.8%). Tuberculous and non-tuberculous mycobacteria were common as a standalone direct cause of death, and less so as a comorbid condition. Across all studies, HIV prevalence ranged from 12%-34%. Both Hib and PCV were in routine use in all the countries included in these studies.

**Table 2:** Aetiology of pneumonia in post-mortem studies

Lead Author and publication date	Country	Specimen types and diagnostic tests	Findings (infection prevalence)	EPHPP Quality Assessment Tool
**AFRO WHO REGION**				
**LOWER-MIDDLE INCOME COUNTRIES**				
Bates 2016 [18]	Zambia	Lung tissue: Xpert MTB/RIF assay RT-PCR, and Ziehl–Neelsen staining.	**N = 121:** TB = 10 (8%), CMV pneumonia 8 (7%), PJP 6 (5%).	Weak
			**HIV negative = 62:** 86% lung pathology; 4 (6%) TB, 4 (6%) CMV, 2 (3%) PJP.	
			**HIV positive = 34**: 100% lung pathology; 5 (15%) TB, 3 (9%) CMV, 3 (9%) PJP.	
			**HIV unknown = 25**: 96% lung pathology; 1 (4%) TB, 1 (4%) CMV, 1 (4%) PJP. Malnutrition – 50% (56/111) of cases with lung pathology – predominant comorbidity for five most prevalent lung pathologies: TB, CMV, PJP, bronchopneumonia, pneumonia, interstitial pneumonitis.	
**UPPER-MIDDLE INCOME COUNTRIES**				
Chawana 2019 [19]	South Africa	Blood and tissue: Histopathology, Fast Track diagnostics kit	12.8% HIV infected on post-mortem. 62.4% of cases were malnourished.	Weak
			**Overall CAP** = 25.2% (32/127). RSV = 21.9% (7/32); PJP = 18.8% (6/32); CMV = 15.6% (5/32); *K. pneumoniae* = 15.6% (5/32); Influenza = 12.5% (4/32); *S pneumoniae* = 12.5% (4/32); *M. catarrhalis* = 9.4% (3/32); *H. influenzae* = 9.4% (3/32); *B. pertussis* = 6.3% (2/32); *P. aeruginosa* = 6.3% (2/32); *S. aureus* = 6.3% (2/32); HMPV = 0.8% (1/32).	
			**1-11 mo: N = 67: All CAP** = 29.9% (20/67). RSV = 35% (7); PJP = 30% (6); CMV = 25% (5); *K. pneumoniae* = 15% (3); Influenza = 5% (1); *M. catarrhalis* 5% (1); *B. pertussis* = 10% (2); *P. aeruginosa* = 5% (1); *S. aureus* = 5% (1); HMPV = 5% (1).	
			**12-59 mo: N = 37: All CAP** = 27% (10/37). *K. pneumoniae* = 20% (2); Influenza = 20% (2); *S pneumoniae* = 40% (4); *M. catarrhalis* = 20% (2); *H. influenzae* = 30% (3); *P. aeruginosa* = 10% (1); *S. aureus* = 10% (1).	
			**≥60 mo: N = 23: All CAP** = 8.7% (2/23). Influenza = 50% (1); Unspecified = 50% (1).	
**MIXED WHO REGIONS AND INCOME CLASSIFICATIONS**				
**CHAMPS (Child Health and Mortality Prevention Surveillance) NETWORK**				
Taylor 2020 [20]	Bangladesh Mali Mozambique South Africa Kenya	Biopsies from lungs, heart, brain, liver, and bone marrow. Peripheral blood, cerebrospinal fluid, stool and nasopharyngeal secretions: Blood and CSF cultures. TaqMan Array molecular assays.	In neonates LRTI’s immediate cause = 86/449 (19%) of deaths; in children LRTI = 143/304 (47%) of deaths. No stillbirths were due to LRTI. **Neonatal deaths** (n = 449 - 240 with infectious cause): *A baumannii* = 50 (20.8%), *K pneumoniae* = 35 (14.6%), *E coli or Shigella* = 7 (2.9%), *S agalactiae* = 3 (1.2%), *S aureus* = 7 (2.9%), *Streptococcus* = 6 (2.5%), *E faecalis* = 1 (0.4%), *S pneumoniae* = 3 (1.2%).	Strong
			**Child deaths (1–59 mos)** (n = 304 – 275 with infectious cause): *K pneumoniae* = 54 (19.6%), *S pneumoniae* = 46 (16.7%), HIV = 3 (1.1%), *Cytomegalovirus* = 24 (8.7%), *A baumannii* = 10 (3.6%), *S aureus* = 22 (8.0%), *H influenzae* = 19 (6.9%), *E coli* = 4 (1.4%), RSV = 17 (6.2%), Adenovirus = 11 (4.0%), PJP = 17 (6.2%), *P aeruginosa* = 9 (3.3%), *Streptococcus* = 9 (3.3%), Parainfluenza virus type3 = 9 (3.3%).	

CMV – *Cytomegalovirus,* PJP – *Pneumocystis jirovecii,* TB – tuberculosis, CAP – community acquired pneumonia, mos – months

A study from Zambia [18] included post-mortem examination of the lungs in 121 children who died in-hospital and 92% had lung pathology. Of the 97 children with HIV results, 34% were HIV-infected with lung pathology observed in all cases. Overall, bacterial bronchopneumonia was the most common pathology (50%), followed by interstitial pneumonitis (17%), tuberculosis (8%), CMV pneumonia (7%) and *P. jirovecii* pneumonia (5%). Malnutrition was the leading comorbidity in all cases (50%). Chawana et al. in South Africa [19] included 127 children up to 14 years of age, 32 (25%) whose immediate or underlying cause of death was CAP in a lower mortality setting. Overall, 12.8% were HIV infected, 23.6% were HIV-exposed uninfected and 62.4% were malnourished. In children 1-11 months where CAP was deemed to be the cause of death, the most common pathogens identified were RSV, PJP (3 HIV-uninfected and 3 HIV-infected), and CMV (2/5 were HIV-infected). The most common pathogens identified in children aged 12-59 months were *S. pneumoniae* and *H. influenzae.* Only two children ≥5 years were included. Of the three post-mortem studies, one was deemed of high quality and two weak as rated by the EPHPP Quality Assessment Tool.

### Empyema or pleural effusion studies

Eight studies included information on the aetiology of pleural effusions and empyema (**Table 3**; Table S3 in the **Online Supplementary Document**) [21-23,25-28]. Most studies did not test for viral pathogens and excluded TB-associated pleural effusions.

**Table 3:** Aetiology of pneumonia in empyema

Lead Author and publication date	Country	Specimen types and diagnostic tests	Findings												EPHPP Quality Assessment Tool
PAHO WHO REGION															
UPPER-MIDDLE INCOME COUNTRIES															
Feris-Iglesias 2014 [21]	Dominican Republic	Viruses: rRT-PCR on pleural fluid.	**Detected by culture n (%)**: SP = 19 (15.7), SA = 19 (15.7), SPy = 1 (0.8), SMi = 1 (0.8), Candida Sp = 1 (0.8), No aetiology = 81 (66.9); **Detected by PCR:** SP = 61 (54.5), SA = 0 (0), SPy = 2 (1.8), SMi = 0 (0), Candida Sp = 0 (0), No aetiology = 49 (43.8); **Detected by culture and/or PCR:** SP = 62 (51.2), SA = 19 (16.7), SPy = 2 (1.7), SMi 1 (0.8), Candida Sp = 1 (0.8), No aetiology = 36 (29.8). Among the 112 samples tested by PCR, no RSV or RV was detected.												Weak
		Bacteria: Pleural fluid culture and PCR.													
SEARO WHO REGION															
LOWER-MIDDLE INCOME COUNTRIES															
Dass 2011 [22]	India	Viruses: Not tested.	**Culture** was positive in 48/150 cases (32%) from pleural fluid. SP = 31/150 (20.7%), SA = 11 (7.3%), KP = 3 (2%), Hib = 2 (1.3%), Enterococcus = 1 (0.7%).												Weak
		Bacteria: Gram stain and culture on pleural pus and blood.	Death = 5 cases (3.4%).												
UPPER-MIDDLE INCOME COUNTRIES															
Lochindarat 2014 [23]	Thailand	Viruses: Not tested.	**Blood sample/Bacterial culture** = 5/66 (8%) positive; SP = 1, HI = 1, SA = 2, En spp = 1; **PF sample/Bacterial culture/local laboratory** = 13/70 (19%) positive; SP = 2, HI = 1, PA = 1, SA = 6, Strep spp = 2, AB = 1; PF sample/Bacterial culture/CIDM = 15/71 (21%) positive; SP = 2, HI = 1, SA = 8, AB = 1, SM = 1; **PF sample/ PCR/CIDM** = 18/71 (25%) positive; SP = 13, HI = 6, MP = 1. Overall CFR = 6/71 (8%)												Weak
		Bacteria: PCR and culture on pleural fluid and blood.													
AFRO WHO REGION															
LOW-INCOME COUNTRIES															
Howie 2014 [24]	The Gambia	**Viruses**: PCR on lung and pleural aspiration; **Bacteria:** Culture, non-molecular serotyping latex agglutination, qPCR, molecular serotyping on lung and pleural aspiration	**Culture and molecular results** (N = 52): SP = 48 (91%), HI = 12 (23%), SA = 3 (6%), Kb species = 2 (4%), RSV = 2 (4%), AdV 2 (4%), EVB = 1 (2%), CoVHKU1 = 1 (2%), INFC = 1 (2%), CMV = 1 (2%), AB species = 3 (6%), EB species 2 (4%), Salm species = 2 (4%), SPs = 1 (2%), BD species 1 (2%), PV species = 1 (2%).												Strong
			**Culture results** = 21/56 (38%) specimens: SP = 14 (25%), HI (non-type b) = 3 (5%), SA = 3 (5%). Ziehl-Neelsen staining = 37/56 (66%), lung aspirate samples (all negative); 35/37 (95%) underwent culture for MTB, and all were negative.												
LOWER-MIDDLE INCOME COUNTRIES															
Kuti 2014 [25]	Nigeria	Bacteria: Culture on pleural fluid	SA = 19 (68%); SP = 2 (7%); KP = 2 (7%); EC = 1 (3.6%); No growth = 4 (14%). Pneumonia with effusions = 4/28 (14.3%); Pneumonia without effusions = 35/324 (10.8%)												Weak
UPPER-MIDDLE INCOME COUNTRIES															
Zampoli 2015 [26]	South Africa	Viruses: Not tested, Bacteria: PCR and culture on pleural fluid and blood	**Cohort A: Blood culture** = 132/142 (93%) – All bacteria = 32 (24%), SP 19 (14%), SA 11 (8%), HI spp = 2 (1.5%), Other strep = 1, Gram-neg organisms = 1; **Pleural fluid culture** = 136/142 (96%) – All bacteria = 45 (33%), SP = 14 (10%), SA = 20 (15%), HI spp = 2 (1.5%), Other strep = 3 (2%), Gram-negative organisms = 3 (2%), MTB = 10/104 (10%); **Combined blood + pleural fluid cultures** = 142 (100%) – All bacteria = 56 (39%), SP = 25 (18%), SA = 25 (18%), HI spp = 3 (2%), Other streptococci = 4 (3%), Gram-negative organisms = 4 (3%); **Pleural Fluid PCR** = 54/142 (38%) – All bacteria = 37 (68.5%), SP = 26 (48%), SA = 9 (17%), HI spp = 3 (5.5%), Other strep = 3 (5.5%).												Weak
			**Cohort B: Combined blood + pleural fluid cultures** = 22 (100%) – All bacteria = 7 (32%), SP = 1 (4.5%), SA = 2 (9%), HI spp = 1 (4.5%), Other strep = 1 (4.5%), Gram-negative organisms = 1 (4.5%), MTB = 3 (14%). Overall = 19/135 (14%) admitted to ICU; 29/135 (21%) needed surgery, 6/135 died (in-hospital mortality 4.4%).												
Ghoor 2018 [27]	South Africa	Viruses: Not tested, Bacteria: Culture, biochemistry, PCR on blood, sputum, pleural fluid and gastric washings	Overall = 36/65 (55.3%) positive, 34 on culture of blood or pleural fluid and 2 isolated by multiplex PCR: SA = 14, 21.5%, SP = 5, 7.7%, MTB = 5, 7.7%, KP = 3, 4.6%. One patient (1.5%) grew both MTB and SA on pleural fluid, while the other 4 cases of MTB were cultured on gastric washings or sputum samples. Incidence of empyema = 1.46 (95% CI = 1.05-1.97) per 100 000 population and 3.40 (95% CI = 2.45-4.59) per 1000 hospitalised cases of acute lower respiratory infection. Complications: 8 (12.3%) thoracotomy; 7 (10.8%) intubation/ventilation; 1 died (case fatality ratio 1.5%)												Weak
MIXED WHO REGIONS AND INCOME CLASSIFICATIONS															
PERCH NETWORK															
Ebruke 2020 [28]	The Gambia, South Africa, Bangladesh, Mali	Viruses and bacteria: Multiplex qPCR on pleural fluid; Bacteria: Culture on pleural fluid	**LUNG ASPIRATE**: **PCR** N = 29: Any positive = 11 (38%), SP = 7 (24%), HI = 4 (14%), CP = 1 (3%), MC = 4 (14%), PJP = 1 (3%), AdV = 1 (3%), CMV = 2 (7%), HMPV = 1 (3%); **Culture** N = 44: Any positive = 5 (11%), SP = 5 (11%), HI = 1 (2%), CP = 0 (0%), MC = 0 (0%), PJP = 0 (0%); **Either PCR or culture** N = 44: Any positive = 13 (30%), SP = 9 (20%), HI = 4 (9%), CP = 1 (2%), MC = 4 (9%), PJP = 1 (2%), AdV = 1 (2%), CMV = 2 (4%), HMPV = 1 (2%), combo: SP+HI = 2 (4%), SP+MC = 2 (4%), AdV+CP = 1 (2%), HI+MC+SP+PJP+CMV = 1 (2%), HI+MC+HMPV = 1 (2%). **PLEURAL FLUID**: **PCR** N = 11: Any positive = 9 (82%), SP = 4 (36%), HI = 1 (9%), SA = 4 (36%), EC = 0 (0), Strep Group F = 0 (0), HBOV = 1 (9%); **Culture** N = 14: Any positive = 9 (64%), SP = 1 (7%), HI = 0 (0%), SA = 7 (50%), EC = 1 (7%), Strep Group F = 1 (7%), HBOV = N/A; **Either PCR or culture** N = 14: Any positive = 12 (86%), SP = 5 (36%), HI = 1 (7%), SA = 7 (50%), E.coli = 1 (7%), Strep Group F = 1 (7%), HBOV = 1 (7%); combo: SA+HBOV = 1 (7%), SA+SP = 1 (7%), EC+SGrF+HI = 1 (7%). Deaths = 2 (4.5%) in patients who had lung aspirate collected												Strong

Bacteria: *SP – Streptococcus pneumoniae, MP – Mycoplasma pneumoniae, SA – Staphylococcus aureus, CP – Chlamydophilia pneumoniae, MC – M. catarrhalis, CB – Coxiella burnetii, MT – Mycobacterium tuberculosis, KP – Klebsiella pneumoniae, AB – Acinetobacter baumanii, EC – Escherichia coli, BP – Burkholderia pseudomallei, LG – Legionella spp, PA – Pseudomonas aeruginosa, AC – Acinetobacter calcoaceticus, HI – Haemophilus influenzae,* Viruses: AdV – adenovirus, BV – bocavirus, CoV – coronavirus, EV – enterovirus, RV – rhinovirus, HMPV – human metapneumovirus, INF – influenza, PIV – parainfluenza virus, RSV – respiratory syncytial virus, CMV – cytomegalovirus; y – year, yo – year old, mo – months

One PERCH network study [28] showed a predominance of bacterial pathogens (*S. pneumoniae* = 20% and *H. influenzae* = 9% in lung aspirate and *S. aureus* = 50% and *S. pneumoniae* = 36% in pleural fluid), which contrasted with overall PERCH findings. One study in The Gambia [24], a high mortality setting, included culture and molecular analysis of pleural effusions and lung aspirates from children 2-59 months with severe pneumonia. A combination of singleplex and multiplex PCRs detected pathogens more frequently than culture, with a predominance of bacteria (*S. pneumoniae* PCR positive = 91% and *S. pneumoniae* culture positive = 25%) [24].

Other studies also showed a preponderance of bacterial causes, especially *S. pneumoniae* and *S. aureus*. Detection rates varied depending on whether culture or PCR was used, and if PCV was introduced. The highest detection rate for *S. pneumoniae* using culture was in a study conducted in India, prior to routine PCV introduction, which detected pneumococci in 20.7% (n = 31/150) of pleural fluid samples [22]; while Feris-Iglesias et al. [21] reported a pneumococcal detection rate of 54.5% (n = 61/112) using PCR pre-PCV introduction in the Dominican Republic. One study from a high mortality setting identified only 28 patients with effusions, two-thirds of which had *S. aureus* identified on pleural effusion culture [25].

A study from South Africa enrolled 65 children <14 years of age with a 20% HIV-positivity rate [27]. More than half of the patients (55.3%) had a bacterial pathogen identified predominantly on culture of blood or pleural fluid. The most common pathogen was *S. aureus* (n = 14), followed by *S. pneumoniae* (n = 5) and *M.tuberculosis* (n = 5); although 28 children were treated for TB despite only a minority having a microbiological diagnosis [27]. Another study from South Africa in children <12 years old, identified *M.tuberculosis* on culture in 12 (8%) cases as part of a prospective cohort and 3 (14%) cases as part a retrospective cohort; there was no difference by HIV status [26]. Of the eight included studies, two were deemed of high quality and six weak as rated by the EPHPP Quality Assessment Tool.

### Surveillance studies

Ten surveillance studies [29-38] tested patients for a variety of viruses and bacteria, using different specimen types (**Table 4**; Table S4 in the **Online Supplementary Document**). In all studies, a high proportion of pneumonia patients (49%-78%) tested positive for one or more respiratory viruses by PCR; most commonly for rhinovirus (15.1%-51.7%), RSV (5.7%-45.9%), influenza (6%-20.4%), HMPV (5%-11%) and adenovirus (5%-21%). Prevalence varied according to age groups and severity of pneumonia cases included in each study. RSV was consistently one of the most common viruses identified in children aged <1 year, with adenovirus, rhinovirus and HMPV also frequently detected [33,35,38]. In all children <5 years, the pattern was similar, while older children (5-14 years) had higher detection rates for influenza and lower for RSV [37,38]. A study from South Africa compared aetiology in HIV-infected and -uninfected children admitted with SARI. HIV-infected cases had more pneumococcal infections (7% vs 4%) detected on whole blood *lytA* PCR or blood culture and more adenovirus (32% vs 27%) than HIV-uninfected children. In contrast, HIV-uninfected children were more likely to have HMPV (7% vs 4%), RSV (27% vs 13%) and >1 virus detected (34% vs 28%) than HIV-infected cases [33].

**Table 4:** Aetiology in surveillance studies

Lead Author and publication date	Country		Specimen types and diagnostic tests	Findings			EPHPP Quality Assessment Tool
EMRO WHO REGION							
LOWER-MIDDLE INCOME COUNTRIES							
				**All cases n (%)**	**By age group n (%)**		
Ali 2016 [29]	Pakistan		**Viruses:** TAG respiratory viral panel on NPS.	N = 817 cases: **BC** performed = 356: All positive BC = 5 (1.4), GAS = 1 (0.3), CB = 2 (0.6), SP = 2 (0.6).	**0-5mos** = 817: **BC** performed = 194: All positive BC = 4 (2.6), GAS = 1 (0.3), CB = 2 (0.6), SP = 1 (0.3); **LA +ve** = 154/201: AdV = 6 (3), BV = 1 (0.5), CoV229E = 1 (0.5), CoVHKU1 = 5 (2.5), CoVNL63 = 3 (1.5), CoVOC43 = 9 (4.5), EV/RV = 110 (54.7), HMPV = 1 (0.5), INFB = 3 (1.5), PIV1 = 1 (0.5), PIV2 = 1 (0.5), PIV3 = 17 (8.5), PIV4 = 10 (5), RSV = 5 (2.5).		Weak
			**Bacteria:** Blood culture.	**Luminex assay (LA) positive** = 179/230: AdV = 8 (3.5), BV = 1 (0.4), CoV229E = 1 (0.4), CoVHKU1 = 5 (2.2), CoVNL63 = 4 (1.7), CoVOC43 = 11 (4.8), EV/RV = 119 (51.7), HMPV = 5 (2.2), INFB = 4 (1.7), PIV1 = 2 (0.9) PIV2 = 2 (0.9) PIV3 = 19 (8.3), PIV4 = 10 (4.3), RSV = 13 (5.7).	**6-23mos** = 797: **BC** performed = 162: All positive BC = 1 (0.6): SP = 1 (0.6); **LA +ve** = 25/29: AdV = 2 (6.9), BV = 0, CoV229E = 0, CoVHKU1 = 0, CoVNL63 = 1 (3.4), CoVOC43 = 2 (6.9), EV/RV = 9 (31), HMPV = 4 (13.8), INFB = 1 (3.4), PIV1 = 1 (3.4), PIV2 = 1 (3.4), PIV3 = 2 (6.9), PIV4 = 0, RSV = 8 (27.6).		
SEARO WHO REGION							
UPPER-MIDDLE INCOME COUNTRIES							
				**All cases n (%)**	**By age group n (%)**		
Olsen 2010 [36]	Thailand		**Viruses**: RT-PCR on NPS, serum.	**All** (n = 3910): CP = 92/3417 (2.7), CB = 3/755 (0.4), MP = 38/3417 (1.1), MT = 92 (2.4), AdV = 100 (2.6), BV = 53/1165 (4.5), CoV229E = 10/1920 (0.5), CoVHKU1 = 11/1920 (0.6), CoVNL63 = 8/1920 (0.4), CoVOC43 = 35/1920 (1.8), INFA = 436 (11.2), INFB = 150 (3.8), HMPV = 60 (1.5), PIV1 = 67 (1.7), PIV2 = 36 (0.9), PIV3 = 164 (4.2), RSV = 597 (15.3), RV = 470/3417 (13.8).	**<5yo** (n = 1325): CP = 13/1152 (1.1), CB = 1/150 (0.7), MP = 11/1152 (1.0), AdV = 70 (5.3), BV = 44/379 (11.6), CoV229E = 3/529 (0.6), CoVHKU1 = 3/529 (0.6), CoVNL63 = 1/529 (0.2), CoVOC43 = 8/529 (1.5), INFA = 117 (8.8), INFB = 39 (2.9), HMPV = 38 (2.9), PIV1 = 35 (2.6), PIV2 = 17 (1.3), PIV3 = 107 (8.1), RSV = 498 (37.6), RV = 242/1152 (21).		Weak
			**Bacteria**: PCR on NPS, ELISA on serum, sputum.		**5-17yo** (n = 408): CP = 3/365 (0.8), MP = 12/365 (3.3), MT = 3 (0.7), AdV = 8 (2), BV = 4/118 (3.4), CoV229E = 1/167 (0.6), CoVNL63 = 1/167 (0.6), CoVOC43 = 2/167 (1.2), INFA = 85 (20.8), INFB = 52 (12.7), HMPV = 5 (1.2), PIV1 = 9 (2.2), PIV2 = 8 (2), PIV3 = 10 (2.5), RSV = 36 (8.8), RV = 55/365 (15.1).		
Baggett 2012 [30]	Thailand		**Viruses**rRTPCR on NPS.	902/7207 (12.5) INF positive cases. Co-infection with RSV: 30 (7.2) INFA(H1N1) pdm09 pts, 29 (11) with H3N2, and 8 (6.7) with INFB virus.	**<5 yo:** 190/2436 (7.8%) INF positive. 38/68 (56) INF-RSV co-infections. No deaths recorded in children.		Weak
			**Bacteria:** Blood culture.	**BC** in 282 (31) of INF-infected patients, and 1 positive for SA. 2336 INF-negative patients had blood cultured; 146 positive BC, including 7 SA & 12 SP.	**5-17yo**: 243 INF positive		
Naorat 2013 [34]	Thailand		**Viruses:** rRTPCR on NPS.	RSV positive <5yo = 802/4839 (16.6); 5-19yo = 74/1802 (4.1); Only in RSV positive group – INFA = 77/1137 (6.8), INFB = 14/1137 (1.2), AdV = 21/1137 (1.9), HMPV = 5/181 (2.8).			Strong
			**Bacteria:** Blood culture.	RSV positive <12months = 230/1182 (19.5); 12-59months = 572/3657 (15.6); RSV positive incidence <5yo = 981 (919-1043) per 100 000 py; 5-19yo = 23 (18-29) per 100 000 py.			
				1750 (3.2) deaths in all age groups; 8 RSV positive deaths – 7 were in ≥50yo.			
Bunthi 2019 [32]	Thailand		**Viruses:** rRTPCR on tracheal aspirates.	**Overall** = 589/972 (60.6%) tested positive for ≥1 pathogen.	**By age group: <5 y:** N = 600, **RSV = 18.3% (110), AdV = 4.7% (28), HMPV = 2.5% (15),** INFA H1N1 = 0.5% (3), PIV3 = 1.8% (11), INFA H3 = 0.8% (5), PIV1 = 0.8% (5), INFB = 0.1% (1), PIV2 = 1% (6), MP = 3.4% (20), CP = 2.5% (15), Hib = 1.5% (9), MC = 20.3% (20), KP = 1% (6), SP = 0.1% (1), SA = 0.1% (1), EC = 0.3% (2). **≥5 y**: N = 372: MP = 21 (3.6%), A(H1N1)pdm09 = 14 (2.4%), RSV = 10 (1.7%)	**Deaths:** Overall = 220/972 (22.6%).	Weak
						**<5 y** = 27.3% (60/220). RSV = 1.2% (7/600), AdV = 0.3% (2/600), HMPV = 3% (2/600), INFA = H1N1 0.1% (1/600), PIV3 = 0.3% (2/600), PIV2 = 0.1% (1/600), MP = 0.1% (1/600), CP = 0.1% (1/600), Hib = 0.1% (1/600), MC = 0.1% (1/600), KP = 0.3% (2/600), SA = 0.1% (1/600).	
				**Virus positive** = 394 (40.5%) of cases; Single virus = 236 (24.3%). RSV = 12.3%, INFA = 3.9%, INFB = 3.9%, AdV = 3.0%.		**5-9 y** = 3.2% (7/220) deaths.	
			**Bacteria**: Blood culture rRTPCR on tracheal aspirate; Fatal cases: NPA, throat swabs, serum, tissue.	**Bacteria positive** = 341 (35%) of cases; Single bacteria = 128 (13.2%). MP = 4.2%, HI = 1.7%, MC = 1.4%, SP = 1.0%. Mixed detection were found in 225 cases (23.1%).		**≥5 y** = 160/372 (43%). MP = 9 (1.5%), A(H1N1) = 7(1.2%), RSV = 3 (0.5%).	
AFRO WHO REGION							
LOW-INCOME COUNTRIES							
				**Overall n (%)**	**By age group**	**Deaths**	
O'Callaghan-Gordo 2011 [35]	Mozambique		**Viruses** Multiplex PCR on NPA.	394/807 (49) +ve with 475 viruses:	**<3mo** = 50 (13), RV = 14 (10), ADV = 1 (2), RSV = 11 (29), HMPV = 6 (21), INF = 4 (14), PIV = 4 (20), EV3 = (30).	**<3mo** = 4/44 (9).	Strong
						**3-<12 mo** = 15/138 (11).	
			**Bacteria:** Blood culture.	RV = 96 (41), ADV = 102 (21), RSV = 50 (11), HMPV = 39 (8), INF = 39 (8), PIV = 31 (7), EV = 18 (4)	**3-12mo** = 149 (38), RV = 67 (50), ADV = 12 (21), RSV 16 (42), HMPV = 16 (55), INF = 7 (25), PIV = 7 (35), EV3 = (30).	**1-5 y** = 14/177 (8).	
					**12-<60mo** = 195 (49), RV = 54 (40), ADV = 44 (77), RSV 11 (29), HMPV 7 (24), INF = 17 (61), PIV 9 (45), EV4 (40).	**HIV +ve** = 10/55 (18)	
Razanajatovo 2018 [37]	Madagascar		**Viruses**: In-house multiplex rtPCR on NPS.	Overall, viral = 667/876 (76) & bacterial = 314/876 (36). **Viruses: N** = **924:** RSV = 348 (37.7), FLUA = 170 (18.4), RV = 125 (13.5), ADV = 77 (8.3), FLUB = 58 (6.3), BV = 40 (4.3), HMPV = 33 (3.6), CoVOC43 = 21 (2.3), CoVNL63 = 15 (1.6), PIV2 = 12 (1.3), PIV1 = 10 (1.1), PIV3 = 9 (1.0), CoV229E = 4 (0.4), CoVHKU1 = 2 (0.2%).		**n (%)<5yrs (N = 710):** FLUA = 121 (17), FLUB 38 (5.4), Influenza = 145 (20.4), COV-OC43 19 = (2.7), COV-NL63 = 14 (2.0), RSV = 326 (45.9), HMPV = 29 (4.1), RV = 107 (15.1), AdV = 69 (9.7), BV = 34 (4.8), SP = 159 (22.4), Hib = 70 (9.9).	Strong
			**Bacteria**: Sputum gram-stain and culture.	**Common bacteria: N** = **370:** SP = 189 (50.3), Hib = 79 (21.4), other Strep spp = 30 (8.1), KP = 17 (4.6), SA = 10 (2.7), EC = 4 (1.1), AB = 3 (0.8).		**5-14yrs (N** = **37)**: FLUA = 7 (18.9), FLUB = 4 (10.8), INF = 11 (29.7), COVOC43 = 2 (5.4), COVNL63 = 0 (0.0), RSV = 8 (21.6), HMPV = 2 (5.4), RV = 6 (16.2), AdV = 3 (8.1), BV = 4 (10.8), SP = 10 (27.0), Hib = 3 (8.1).	
LOWER-MIDDLE INCOME COUNTRIES							
				**Overall n (%)**		**Incidence by age**	
Berkley 2010 [31]	Kenya		**Viruses** rRTPCR on NPW.	**LRTI** overall positive = 56% (425); 36 (4.7%) bacteraemic, with 16 having respiratory virus detected (44%).		**Incidence (per 100 000 children by age group)**	Weak
						**All LRTI:** Age <5 = 1522; Age 5-<13 = 99.	
						**RSV:** Age <5 = 535; Age 5-<13 = 15.	
				Bacterial species were SP (12), *E coli* (9), NTS (3), SA (3), Acinetobacter species (3), Beta-haemolytic streptococci (3), Enterobacter species (2), and HI (1).		**CoV299E**: Age <5 = 105; Age 5-<13 = 3.	
						**INFA:** Age <5 = 82; Age 5-<13 = 15.	
						**PIV3**: Age <5 = 57; Age 5-<13 = 6.	
						**AdV**: Age <5 = 55; Age 5-<13 = 9.	
			**Bacteria**: Blood culture.	**URTI** Overall positive = 44% (42); **Well control** overall positive = 28% (16).		**HMPV**: Age <5 = 44; Age 5-<13 = 6.	
				**LRTI group:** RSV = 27% (206/759); Non-RSV = 22% (165/759); **URTI group**: RSV = 16% (15/96); Non-RSV = 26% (25/96); **Well group**: RSV = 4% (2/57); Non-RSV = 23% (13/57).		Deaths: 24 deaths in LRTI group, with 8 in virus positive children (1.9%).	
UPPER-MIDDLE INCOME COUNTRIES				**Overall n (%)**	**By age group**	**By HIV status**	
Cohen 2015 [33]	South Africa		**Viruses**: rRT-PCR on NPA.	**Invasive bacterial infection** on culture = 75/3196 (2%); SP = 253/6612 (4%); **Any virus** identified = 6517/8393 (78%); >1 virus = 2760/8393 (33%); INF = 613/8394 (7%); INFB = 171/8394 (2%); EV = 877/8393 (10%); RV = 3115/8393 (37%); HMPV = 504/8393 (5%); PIV1 = 161/8392 (2%); PIV2 = 116/8392 (1%); PIV3 = 535/8392 (6%); PIV1-3 = 789/8393 (9%); RSV = 2216/8393 (26%); Any aetiology identified = 6635/8723 (76%).	**0-3 Months** n/N (%) **Viruses:** INF = 109/2726 (4); AdV = 298/2558 (12); EV = 207/2725 (8); RV = 816/2725 (30); RSV = 897/2725 (33); Any respiratory virus = 1883/2725 (69); >1 Respiratory virus = 602/2725 (22); **IBD** on culture = 32/1440 (2); SP = 70/2254 (3); **4-11 Months** n/N (%) **Viruses:** INF = 201/2637 (8); ADV = 646/2448 (26); EV = 245/2637 (9); RV = 1027/2637 (39); HMPV = 211/2637 (8); RSV = 717/2637 (27); Any virus = 2146/2637 (81); >1 virus = 932/2637 (35); **IBD** = 19/876 (2); SP = 75/2063 (4).	**HIV-infected** n/N (%) **Viruses:** INF = 49/688 (7); AdV = 198/628 (32); EV = 56/688 (8); RV = 245/688 (36); HMPV = 26/688 (4); PIV = 62/688 (9); RSV = 88/688 (13); Any virus = 466/688 (68); >1 virus = 190/688 (28); **IBD** on culture = 12/311 (4); SP = 44/642 (7). **HIV-uninfected** n/N (%) **Viruses:** INF = 362/5161 (7); AdV = 1298/4837 (27); EV = 513/5160 (10); RV = 1952/5160 (39); HMPV = 357/5160 (7); PIV = 486/5160 (9); RSV = 1412 /5160 (27); Any virus = 4098/5160 (79); >1 virus = 1733/5160 (34); **IBD** on culture = 51/2092 (2); SP = 180/4786 (4)	Strong
					**12-23 Months** n/N (%) **Viruses:** INF = 153/1703 (9); ADV = 600/1559 (38); EV = 249/1703 (15); RV = 677/1703 (40); HMPV = 97/1703 (6); PIV3 = 126/1702 (7); RSV = 374/1703 (22); Any virus = 1410/1703 (83); >1 virus = 676/1703 (40); **IBD** = 14/499 (3); SP = 59/1302 (5).		
			**Bacteria**: Blood for lytA PCR and culture.		**24-59 Months** n/N (%) **Viruses:** INF = 150/1328 (11); ADV = 517/1234 (42); EV = 176/1328 (13); RV = 595/1328 (45); HMPV = 67/1328 (5); RSV = 228/1328 (17); Any virus = 1078/1328 (81); >1virus = 550/1328 (41); **IBD** = 13/381 (3); SP = 49/993 (5).		
PAHO WHO REGION							
UPPER-MIDDLE INCOME COUNTRIES				**Overall n (%)**		**By age group**	
Verani 2013 [38]	Guatemala		**Viruses**PCR on NP/OP swab.	50.4% of patients had at least one virus detected, and 365 (9.4%) tested positive for two or more viruses. The most common pathogens isolated among the patients with blood cultures results were SA (n = 32, 2.4%) and SP (n = 12, 0.9%).		**<1yo** n = 1349, RSV = 39%, AdV = 5%, HMPV = 6%, INFA = 5%, INFB = 0.5%, PIV1 = 1.5%, PIV2 = 1%, PIV3 = 5%.	Weak
						**1-4yo** n = 641, RSV = 22%, AdV = 8%, HMPV = 11%, INFA = 6%, INFB = 2%, PIV1 = 2%, PIV2 = 1%, PIV3 = 5%.	
			**Bacteria**: PCR on NP/OP swab & blood culture.			**5-14yo** RSV = 8%, AdV = 10%, HMPV = 3%, INFA = 7%, INFB = 3%, PIV1 = 2%, PIV2 = 1%, PIV3 = 3%.	
						3%-5% of cases died.	

Bacteria: *GAS – group A strep, CB – campylobacter, SP – Streptococcus pneumoniae, Spy – Streptococcus pyogenes, SMi – Streptococcus mitis, MP – Mycoplasma pneumoniae, SA – Staphylococcus aureus, CP – Chlamydophilia pneumoniae, CB – Coxiella burnetii, MT – Mycobacterium tuberculosis, KP – Klebsiella pneumoniae, AB – Acinetobacter baumanii, EC – Escherichia coli, BP – Burkholderia pseudomallei, LG – Legionella spp, PA – Pseudomonas aeruginosa, AC – Acinetobacter calcoaceticus, HI – H. influenza, SH – Staphylococcus haemolyticus, SM – Stenotrophomonas maltophilia, PM – Proteus mirabilis, SeM – Serratia marcescens;* Viruses: AdV – adenovirus, BV – bocavirus, CoV – coronavirus, EV – enterovirus, RV – rhinovirus, HMPV – human metapneumovirus, INF – influenza, PIV – parainfluenza virus, RSV – respiratory syncytial virus;y – year, yo – year old, mo – months

For bacterial diagnosis, studies that included blood cultures [29-31] had low positivity (3%-4%); while those that used other samples (tracheal aspirates or sputum) or PCR had higher positivity rates. A study in Madagascar, a high mortality setting, reported detection rates of 22.4% for *S. pneumoniae* and 9.9% for Hib on sputum culture in 710 children <5 years with routine PCV and Hib vaccine use [37]. Hib was introduced in 2008 with coverage reported as 71%-74% using WHO-UNICEF estimates [37]. One study from Thailand, which defined TB using the WHO definition of ≥2 acid fast bacilli sputum smear-positive results or one positive smear with an abnormal chest radiograph, detected no TB cases in children aged <5 years and only 3 cases in children 5-17 years [36].

Most studies only reported overall deaths in children with pneumonia [29,36]. Bunthi et.al. described pathogens detected in fatal and non-fatal pneumonia cases in a low mortality setting [32]. Participants with severe pneumonia were recruited across 30 different health care sites in Thailand. In children <5 years, 60 (10%) cases died and 31 (52%) had positive laboratory results. The most common pathogens detected were RSV, adenovirus, HMPV and *K. pneumoniae* [32]. Of the ten surveillance studies, four were deemed of high quality and six weak as rated by the EPHPP Quality Assessment Tool.

### Cohort studies

The eight cohort studies included in the review [39-46] had a study period ranging from 1-4 years (**Table 5**; Table S5 in the **Online Supplementary Document**). Only one study was in the post-PCV period [41]. Viruses were detected in respiratory samples using PCR, with one study also using virus-specific serum antibody titres [46]. The most common respiratory viruses detected were rhinovirus (31%-40.1%), adenovirus (19.1%-50%), RSV (12.9%-16.9%), influenza (45.7%), and enterovirus (25.3%). Culture was predominantly used for bacterial detection, with or without PCR. Most studies showed low rates of positive blood culture (1%-5.4%); the exception was a study in rural Mozambique, a high mortality setting, with high rates of HIV and PJP, which showed a blood culture positivity rate of 14.8% [43]. Nearly half of these cultures were positive for pneumococcus and a quarter for Hib; however, this study was conducted prior to the introduction of PCV and Hib vaccine. A later study from the same site in Mozambique, following the introduction of Hib vaccine, detected 22 cases (7.9%) with positive blood cultures [40]. The most common bacteria identified were *S. pneumoniae*, Hib, and non-typhoidal *Salmonella* (individual numbers not reported) and the most common virus identified in both HIV-infected (31.2%) and HIV-uninfected children (44.7%) was rhinovirus. HIV-infected cases had more RSV (16.5% vs 10.5%), parainfluenza (10.1% vs 2.6%), bocavirus (9.3% vs 2.6%), and influenza (6.8% vs 5.3%) than HIV-uninfected children. In contrast, HIV-uninfected children were more likely to have adenovirus (28.9% vs 17.3%) and HMPV (10.5% vs 8%) than HIV-infected cases [40].

**Table 5:** Aetiology of cohort studies

Lead Author and publication date	Country	Specimen types and diagnostic tests	Infection prevalence in LRTI cases (overall)	Infection prevalence in LRTI cases (by age group) and sequelae/deaths	EPHPP Quality Assessment Tool
AFRO WHO REGION					
LOW- INCOME COUNTRIES					
Lanaspa 2015 [43]	Mozambique	**Viruses:** PCR on blood, NPA.	PCP = 6.8% (57) positive.	PCP positive group: 0-12 mo = 84.2% (48/57), 1-5 y = 15.8% (9/57).	Weak
			BC = 108/730 (14.8%) positive.	PCP negative group = 777. 0-12 mo = 51.2%, 1-5 y = 48.8%.	
		**Bacteria**: Culture on blood, NPA.	SP = 42.3% (46/108), Hib = 23.1% (25/108), enteric Gram-negative bacilli = 10.2% (11/108).	PCP case fatality rate = 20.8%, non-PCP case fatality rate = 10.2%.	
			Viral detection in NPA = 392/806 (48.6%) positive for respiratory viruses, with multiple infections being common (76/392, 19.4% of positive NPA).	PCP Prevalence = 14.3% HIV-positive; PCP Prevalence = 3.3% in HIV-negative.	
Annamalay 2016 [40]	Mozambique	**Viruses:** RT-PCR on NPA.	All cases = 206/277 (74.4%) tested positive on NPA: RV = 92 (33.2%), AdV = 19.1%, RSV = 15.5%.	RSV-positive children (mean age = 8.9 mo) were younger than RSV-negative children (mean age = 13.4 mo, *P* = 0.022). Adenovirus-positive children (mean age = 18.6 mo) were older than adenovirus-negative children (mean age = 11.5 mo).	Weak
			Bacteraemia all cause 22 (7.9%).		
		**Bacteria**: Blood culture.	HIV-uninfected (n = 237): RV = 44.7%, AdV = 28.9%, RSV = 10.5%, PIV = 2.6%, HMPV = 10.5%, BV = 2.6%, INF = 5.3%, EV = 2.6%.	Of the RV-A positive cases = 23/47 (48.9%) were <12 mo old.	
			HIV-infected (n = 38): RV = 31.2%, AdV = 17.3%, RSV = 16.5%, PIV = 10.1%, HMPV = 8%, BV = 9.3%, INF = 6.8%, EV = 4.2%, CV = 1.7%.	Of the RV-C positive cases = 15/35 (42.9%) were <12 mo old.	
**LOWER-MIDDLE INCOME COUNTRIES**					
Assane 2018 [41]	Senegal	**Viruses:** RT-PCR on BAL, sinus fluids, throat swab.	AdV = 81 (50%), INF = 74 (45.7%), RV = 65 (40.1%), EV = 41 (25.3%), RSV = 26 (16.1%).	**0-6 mo** AdV = 17, INF = 15, RV = 18, RSV = 10, EV = 8, Hib = 2, SP = 7, MC = 3, other = 2.	Weak
				**6-12 mo** AdV = 11, INF = 7, RV = 10, RSV = 6, EV = 8, Hib = 1, SP = 5, MC = 4, other = 2.	
			Single AdV infection rare = 3.7% (6). AdV associated with other viruses = 25.31% (41) and bacteria and = 4.94% (8).	**12-14 mo** AdV = 20, INF = 20, RV = 15, RSV = 4, EV = 7, Hib = 3, SP = 11, MC = 11, other = 1.	
		**Bacteria:** Culture BAL, sinus fluids, throat swab.	INF single-virus co-infections = 33.3% (54), virus & bacteria co-infections = 12.35% (20), RV and EV single infections = 1.85% (3).	**24-60 mo** AdV = 29, INF = 23, RV = 17, RSV = 5, EV = 14, Hib = 6, SP = 4, MC = 7, other = 6.	
			SP = 29 (17.9%), MC = 25 (15.43%), HI = 13 (8.02%). Bacterial single infections rare: SP = 2%, MC = 2%, HI = 1%.	**60-112 mo** AdV = 4, INF = 9, RV = 5, RSV = 1, EV = 4, Hib = 1, SP = 2, MC = 0, other = 0.	
SEARO WHO REGION					
LOWER-MIDDLE INCOME COUNTRIES					
Jullien 2020 [42]	Bhutan	**Viruses:** Multiplex RT-PCR on NPW.	IBD: All positive **blood culture** = 8/148 (5.4%), SP = 2/148 (1.4%), SP **RT-PCR in dried blood spot** sample (Ct LytA) = 1/148 (0.7%), All positive pleural culture = 1/1 (100%), SP = 1/1 (100%).	6/189 (3.2%) children died; 30 children PICU	Weak
		**Bacteria:** Blood culture; RT-PCR (lytA) on blood.	Viral detection: **Rapid flu test** = 9/32 (28%); **NPW** positive = 103/115 (89.6%), Single viral infection in NPW = 68/103 (66%), Mixed viral infection in NPW = 35/103 (34%), RSV = 52/115 (45.2%), RV = 42/115 (36.5%), PIV = 19/115 (16.5%), INF 16/115 (13.9%), AdV = 8/115 (7.0%), BV = 6/115 (5.2%), HMPV = 4/115 (3.5%), CoV = 2/115 (1.7%).		
Mathew 2015 [44]	India	**Viruses:** Multiplex PCR on NPA, BAL.	**Bacterial culture: Blood culture** = 49/2285 (2.1%): SA = 15, SP = 10, HI = 4, KP = 6, AB spp = 5, ST = 3, EB spp = 1, EC = 1, PS spp = 0, SM = 0, Yeast spp = 0, Multiple = 4; **NPA culture** 322/2323 (13.9%): SA = 22, SP = 255, HI = 31, KP = 3, AB spp = 1, ST = 0, EB spp = 0, EC = 3, PS spp = 4, SM = 1, Yeast spp = 1, Multiple = 1; **BAL culture** 3/30: SA = 1, SP = 1, AB spp = 1.	108 (4.6%) deaths; Mortality rate for pneumonia = 1.2%, severe pneumonia = 4.7%, very severe pneumonia = 15.8%.	Moderate
		**Bacteria**: Culture, Serology, Multiplex PCR on Blood, NPA, BAL.	**NPA PCR** = 422/428 (98.6%) positive = 352 (82.2%) multiple = 70 (16.4%) single: SP = 35 (50%), CMV = 13 (18.6%), RSV = 9 (12.9%), other viruses = 6 (8.7%), SA = 5 (7.1%), HI = 2 (2.9%). Total numbers SP = 327, HI = 133, SA = 86, RSV A/B = 103, INF = 15, PIV = 32, AdV = 16, RV = 45, CV = 34, EV = 15, HMPV = 12, PaV = 4, SARS = 4, CMV = 236, MP = 3, CP = 0; **BAL PCR** (n = 30) single pathogens = 10 (SP = 3, CMV = 3, SA = 2, HI = 2) and multiple = 18; **Serology positive** MP = 103 (4.3%), CP = 26 (1.1%).		
UPPER-MIDDLE INCOME COUNTRIES					
Aman 2020 [39]	Indonesia	**Viruses**: rRTPCR, ELISA, serology respiratory and blood.	All ages = 242 (57.6%) tested positive. Influenza = 51 (3), RSV = 11 (1), Measles = 11, MTB = 12 (5), KP = 6, SP = 6 (1), PA = 6 (1), AB = 5 (1).	1-5 y = 54/104 (51.9%), Influenza = 11/48 (22.9%), bacteria = 3/41 (7.3%), resp viruses = 20/29 (69%), 4 deaths (3.8%).	Weak
		**Bacteria**: RTPCR, culture on respiratory specimens, blood, faeces.	No TB cases in children <18yo.	5-18 y 38/106 (35.8%). Influenza = 9/48 (18.8%), bacteria = 4/41 (9.8%), resp viruses = 3/29 (10.3%), 3 deaths (2.8%).	
WPRO WHO REGION					
UPPER-MIDDLE INCOME COUNTRIES					
Nathan 2020 [45]	Malaysia	**Viruses:** Multiplex PCR on induced sputum.	Overall = 186/300 (62%).	No deaths reported	Moderate
			Viruses: **IS PCR**: virus alone = 23.7% (71) and virus together with bacteria = 13% (40). Viruses alone were RV = 22 (31.0%), RSV = 12 (16.9%), HMPV = 16 (22.5%), INF = 4 (5.6%), PIV = 3 (4.2%), AdV = 3 (4.2%), BV = 2 (2.8%) and multiple viruses = 9 (12.7%).		
		**Bacteria**: Bacterial culture, PCR on induced sputum and blood.	Bacteria: **IS PCR** = 65.4% (91/139) as bacteria alone, together with a virus = 33.8% (47/139). HI = (57), SA = (56), SP = (37), MP = (1), BP (2), MC = (4). **Blood PCR**: SA = (4). In 19 patients (13.7%), >1 bacteria were detected via PCR. **Blood cultures** were positive for 3 (1%) children: HI = (1), SP = (1) and SA = (1).		
Zhang 2011 [46]	China	**Viruses:** Virus-specific serum antibody titres on acute and convalescent serum using ELISA, Ag & DFA on NPS.	**Viral cases**: Total = 353/821 (43%); RSV = 149/821 (18%); PIV = 62/821 (8%); INF = 75 (9%); AdV = 67 (8%).	**Age <1yo n = 320:** RSV = 75 (23%); PIV = 28 (9%); INF = 27 (8%); AdV = 35 (11%); Total viral rate = 165 (52%). SP = 26 (8%); Hib = 35 (11%); MC = 10 (3%); MP = 10 (3%); Total bacterial rate = 71 (22%).	Moderate
				**Age 1-3yo n** = **221:** RSV = 35 (16%); PIV = 15 (7%); INF 17 (8%); AdV = 17 (8%); Total viral rate = 84 (38%). SP = 56 (25%); Hib = 40 (18%); MC = 2 (0.9%); MP = 17 (8%); Total bacterial rate = 98 (44%).	
		**Bacteria:** Bacterial antibody assays on acute and convalescent serum samples using ELISA.	**Bacterial cases**: Total rate = 228/821 (28%); SP = 119/821 (14%); Hib = 95/821 (12%); MC = 14/821 (1.7%); MP = 93/821 (11%).	**Age 3-5yo n = 147:** RSV = 22 (15%); PIV = 10 (7%); INF = 14 (10%); AdV = 9 (6%); Total viral rate = 55 (37%). SP = 16 (11%); Hib = 10 (7%); MC = 1 (0.7%); MP = 24 (16%); Total bacterial rate = 27 (18%).	
			107 (13%) children had mixed viral bacterial infection. Of those with RSV, 37% (55/149) had concurrent bacterial infection.	**Age ≥5yo n = 133:** RSV = 17 (13%); PIV = 9 (7%); INF = 17 (13%); AdV = 6 (5%); Total viral rate = 49 (37%). SP = 21 (16%); Hib = 10 (8%); MC = 1 (0.8%); MP = 42 (32%); Total bacterial rate = 32 (24%).	
				ICU admissions = 98 (12%); 5 died (CFR 0.6%).	

BC – blood culture, NPW – nasopharyngeal washing, IS – induced sputum; *SA – Staph aureus, SP – Streptococcus pneumoniae, HI – Haemophilus influenzae, KP – Klebsiella pneumoniae, AB – Acinetobacter spp, ST – Salmonella typhi, EB – Enterobacter spp, EC – Enterococcus coli, PS – Pseudomonas spp, SM – Stenotrophomonas maltophila*; CMV – cytomegalovirus, RSV – respiratory syncytial virus, INF – influenza - 15, PIV – parainfluenza, AdV – adenovirus, RV – rhinovirus, CV – coronavirus, EV – enterovirus, HMPV – human metapneumovirus, PaV – parechovirus, BV – bocavirus, SARS – severe acute respiratory syndrome, MP – mycoplasma pneumoniae, CP – chlamydophila pneumoniae, MC – M. catarrhalis; BP – bordetella pertussis; y – year, yo – year old, mo – months

One study used bacterial antibody assays, with 14% of patients positive for pneumococcus and 12% for Hib [46]. Nathan et al. [45] enrolled children with WHO-defined (2013) very severe pneumonia [47] and collected induced sputum and blood samples. Single virus infections were detected in 23.7% (n = 71; rhinovirus (31%), HMPV (22.5%), RSV (16.9%)), and single bacterial infections in 25% (n = 75; *H. influenzae* (29.3%), *S. aureus* (24.0%), *S. pneumoniae* (22.7%)). Co-infections were detected in 40 (13.3%) patients [45]. Of the eight cohort studies, three were deemed of moderate quality and five weak as rated by the EPHPP Quality Assessment Tool.

### Cross-sectional studies

Four cross-sectional studies (**Table 6**; Table S6 in the **Online Supplementary Document**) were included in the review [48-51]. All studies were conducted prior to PCV introduction, and only one study included all ages. Nascimento-Carvalho et al. [49] identified an aetiology in 86.2% of 181 enrolled CAP cases using ELISA and PCR on nasopharyngeal aspirates for viruses and blood culture. ELISA was used in paired serum samples and PCR on serum for bacteria; 84 (46.4%) had viral infections, 26 (14.4%) bacterial infections, 46 (70.8%) mixed viral-bacterial infections, 18 (27.7%) viral-viral infections, and 1 (1.5%) bacterial-bacterial infection. Severe/very severe CAP was detected among 67 (73.6%) cases with a single infection, and 48 (73.8%) with co-infections. There was a similar frequency of viral infection in WHO-defined (2000) severe/very severe and non-severe cases (*P* = 0.90) [52]; whereas pneumococcal infections increased significantly across the severity of cases (*P* = 0.04) in children aged 2-59 months [49].

**Table 6:** Aetiology of cross-sectional studies

Lead Author and publication date	Country	Specimen types and diagnostic tests	Findings	EPHPP Quality Assessment Tool
AFRO WHO REGION				
LOWER-MIDDLE INCOME COUNTRIES				
Kwofie 2012 [48]	Ghana	**Viruses:** RT-PCR on NPS.	≥1 virus = 33/128 (25.7%). Multiple viral infections in 2 patients. Bacteria positive = 12 (9.4%) patients – SA = 10, Kb species = 1, Coliform = 1. RSV and SA co-infection = 2.	Weak
		**Bacteria**: Conventional biochemical methods and culture on blood.	**≤5 mo** (n = 30)≥1 virus = 6 (20.0), RSV = 4 (13.3), AdV = 2 (6.7), PIV1 = 0 (0.0), PIV3 = 0 (0.0), INFB = 0 (0.0); **6-23 mo** (n = 59)≥1 virus = 18(30.5), RSV = 9(15.3), AdV = 8(13.6), PIV1 = 1(1.7), PIV3 = 2 (3.4), INFB = 1 (1.7); **24-60 mo** (n = 39)≥1 virus = 9(23.1), RSV = 5(12.8), AdV = 3(7.7), PIV1 = 1(2.6), PIV3 = 1(2.6), INFB = 0(0.0).	
PAHO WHO REGION				
UPPER-MIDDLE INCOME COUNTRIES				
Nascimento-Carvalho 2016 [49]	Brazil	**Viruses:** PCR and ELISA on NPA.	**N (%):** SP = 39 (21.5), HI = 13 (7.2), MP = 11 (6.1), CT = 9 (5.0), MC = 4 (2.2), SN = 3 (1.7), RV = 39 (21.5), RSV = 36 (19.9), PIV = 35 (19.3), INFA/B = 15 (8.3), BV = 17 (9.4), AdV = 10 (5.5), EV = 10 (5.5), HMPV = 8 (4.4).	Weak
			**Sole bacterial infection**: Non-severe = 3/24 (12.5%); Severe = 17/58 (29.3%); Very severe = 5/9 (55.6%).	
		**Bacteria**: Blood culture, ELISA in paired serum samples, PCR on serum.	**Sole viral infection**: Non-severe = 21/24 (87.5); Severe = 41/58 (70.7%); Very severe = 4/9 (44.4%).	
			**Overall:** Viral infection similar severe/very severe and non-severe cases (46.1% vs 47.2%; *P* = 0.9). Pneumococcal infection increased non-severe (13.2%), severe (23.4%), very severe (35.3%) cases (*P* = 0.04). Frequency **sole bacterial infection** different (*P* = 0.04) among non-severe (12.5%), severe (29.3%) or very severe (55.6%).	
WPRO WHO REGION				
UPPER-MIDDLE INCOME COUNTRIES				
Xu 2018 [50]	China	**Viruses**: RT-PCR on throat swabs.	Among 585 samples, single infection = 36.41% (213), multiple infections = 9.91% (58). Positive detection rate: <5 yo = 67/96 (69.79%); 5-14 yo = 49/62 (79.03%)	Weak
		**Bacteria**: Particle agglutination antibody test on serum.	**<5yo:** MP = 21 (21.88), INFA/B = 9 (9.38), AdV = 4 (4.17), RSV A/B = 8 (8.33), PIVs = 1 (1.04), CoV = 1 (1.04), RV = 1 (1.04), BoV = 1 (1.04). **5-14yo**: MP = 24 (38.71), INFA/B = 3 (4.84), AdV = 2 (3.23), RSV A/B = 1 (1.61), PIV = 1 (1.61).	
Zhong 2019 [51]	China	**Viruses**: RT-PCR on NP secretions.	1181 (88.5%) positive ≥1 virus or atypical bacteria; Viral infection = 1138 (85.2%). Detection rates: HPIV = 203 (15.2%), INFA = 67 (5.0%), INFB = 36 (2.7%), RV = 414 (31%), RSV = 440 (33%), HMPV = 93 (7%), CoV = 40 (3%), AdV = 115 (8.6%), BV = 54 (4%), MP = 69 (5.2%), CP = 25 (1.9%).	Weak
			Co-infection rates: HPIV = 24.8%, CoV = 65.0%, INFB = 63.9%, BV = 59.3%, AdV = 56.5%, RV = 51.7%.	
		**Bacteria**: RT-PCR on NP secretions or sputum.	Positivity rate all pathogens: children 1-11 mo = 88.5% (684/773), 12-35 mo = 91.4% (352/385), 36-71 mo = 81.9% (145/177).	
			Positivity rate PIV only: children 1-11 mo = 88.5% (684/773), 12-35 mo = 91.4% (352/385), 36-71 mo = 81.9% (145/177).	

Bacteria: *SP* – *Streptococcus pneumoniae, HI* – *Haemophilus influenza, SA* – *Staphylococcus aureus, Kb* – *Klebsiella species, AB* – *Acinetobacter species, EB* – *Enterobacter species, Salm* – *Salmonella species, SPs* – *Streptococcus pseudopneumoniae, BD* – *Bacteroides species, PV* – *Prevotella species, MTB* – *Mycobacterium tuberculosis, MP* – *M. pneumoniae, CT* – *C. trachomatis, MC* – *M. catarrhalis, SN* – *S. negevensis*; Viruses: RSV – respiratory syncytial virus, AdV – adenovirus, EV – enterovirus, CoV – coronavirus, INF – influenza, CMV – cytomegalovirus, RV – rhinovirus, PIV – parainfluenza, BV – bocavirus, HMPV – human metapneumovirus; y – year, yo – year old, mo – months

Bacteria identified varied across studies depending on specimens taken and diagnostics used. In children with severe pneumonia in Ghana, the most common bacterium identified was *S. aureus* [48], while in Brazil, *S. pneumoniae* and *H. influenzae* were detected most frequently using culture, PCR, and ELISA [49]. In China, *Mycoplasma pneumoniae* was detected most commonly using a serum antibody test [50] or PCR on respiratory secretions [51]. The most common viruses detected across studies included RSV (14.1%-33%), rhinovirus (21.5%-31%), parainfluenza virus (3.1%-19.3%), and adenovirus (5.5%-10.2%) [48-51]. Parainfluenza virus co-infection with atypical bacteria was associated with longer hospital admissions than single parainfluenza virus infections [51]. Of the four cross-sectional studies, all were rated by the EPHPP Quality Assessment Tool as weak.

### Other studies

The 12 remaining studies [53-64], included a variety of study designs (**Table 7**; Table S7 in the **Online Supplementary Document**). One study was a secondary data analysis from the GABRIEL Network [56], which reported the detection of influenza viruses in 888 hospitalised children aged 2 to 60 months with radiologically confirmed pneumonia. Influenza virus was identified in 9.7% of children; other common viral causes detected were RSV (20.0%) and rhinovirus (24.9%). Although high bacterial carriage was detected on respiratory samples, blood culture was positive in only 2.7% of cases. The use of blood RT-PCR testing increased the detection of bacteria (*S. aureus* 1.8%, *S. pneumoniae* 10.4% and *H. influenzae* 3.4%), but this may also be reflective of carriage.

**Table 7:** Aetiology of other studies

Lead Author and publication date	Country	Specimen types and diagnostic tests	Infection prevalence in LRTI cases and deaths	Infection prevalence in LRTI cases (by age group) and deaths				EPHPP Quality Assessment Tool
WPRO WHO REGION								
LOWER-MIDDLE INCOME COUNTRIES								
Dembele 2019 [57]	Philippines	**Viruses:** PCR on NPS.	Of 5054 NPS 61.0% tested positive for at least one virus. RSV = 1352/5054 (27.0%), RV = 1156/5054 (23.0%).	**2-59 mo NPS** (n = 4305): **Viruses** RSV = 1021 (23.7%); INF = 163(3.8%); RV = 812 (18.9%); EV = 63 (1.5%); AdV = 49 (1.1%); HMPV = 163 (3.8%); PIV = 116 (2.7%); Multiple viruses = 185 (4.3%); **Bacteria** = 42/2542 (1.7%).				Weak
		**Bacteria** Blood culture.		**CFR: <2mo** = 40/749 (CFR = 5.3); **2-5mo** = 76/1087 (CFR = 7); **6-11mo** = 50/1114 (CFR = 4.5) **12-35mo** = 59/1736 (CFR = 3.4); **36-59mo** = 13/368 (CFR = 3.5).				
Guerrier 2013 [59]	Cambodia	**Viruses:** PCR on NPA.	**Viruses**: ≥1 viral pathogens = 551/1006 (55%). Single virus = 491/1006 (49%) RV = 169 (34%), RSV = 167 (34%), PIV = 40 (8%), HMPV = 39 (8%), INF = 31 (6%), BV = 16 (3%), AdV = 14 (3%), CoV = 9 (2%) and EV = 5 (1%).	Virus positive in pneumonia cases: 0-11 mo = 98 (35%); 12-23 mo = 85 (52%); 24-59 mo = 56 (51%).				Weak
		**Bacteria** Blood culture.	Pneumonia cases no viruses = 184/423 (44%), 1 virus = 198 (47%), 2 viruses = 40 (9%), 3 viruses = 1 (0.2%), RV = 95 (40%), RSV = 64 (27%). **Bacteria** = 10/672 (1.4%) positive: SA (3), SP = (2), HI = (2), *B. pseudomallei* = (2) and KP = (1).	Twelve patients died (7 pneumonia and 5 bronchiolitis).				
Huong 2014 [60]	Vietnam	**Viruses:** RTPCR on BAL.	All = 215 (29.78%) cases were positive for atypical pathogens. MP = 190/215 (88.37%); CP = 13/215 (6.05%); LP = 12/215 (5.58%).	1-2yo = 120, Mp = 37.1%, CP = 2.1%, LP = 2.1%, Mixed = 5.2%.				Weak
			Severe-ApCAP group = 97/215 (45.12%), MP = 84/97 (86.60%), CP = 6/97 (6.19%), LP = 7/97 (7.22%); Co-infection with bacteria = 27.83% (27/97): SP = 14/27, HI = 8/27, co-infection with respiratory viruses = 13.4% (13/97): RSV = 2/13, INF = A/B virus = 3/13, AdV = 4/13, RV = 4/13.	>2-5yo = 47 Mp = 26.8%, CP = 3.1%, LP = 4.1%, Mixed = 2.1%.				
		**Bacteria** Culture & multiplex PCR on BAL, Serum serology.	Non-severe ApCAP = 118/215 (54.88%), MP = 106/118 (89.83%), CP = 7/118 (5.93%), LP = 5/118 (4.24%); Co-infection with bacteria = 9.3% (11/118): SP = 4/118, HI = 4/118, co-infection with respiratory viruses = 5.1% (6/118): RSV = 0, INF A/B virus = 0, AdV = 0, RV = 4/6, Other viruses = 2/6.	>5-10yo = 39, Mp = 12.4%, CP = 1.0%, LP = 1.0%, Mixed = 1.0%.				
UPPER-MIDDLE INCOME COUNTRIES								
Chen 2013 [54]	China	**Viruses:** DFA and RT-PCR on NPA.	295/1598 (18.5%) – MP alone = 199 (12.5%), CP alone = 81 (5.1%), co-infected = 15 (5.1%).	**By age: <1yo:**MP = 80/817 (9.8%), CP = 40/817 (4.9%), co-infxn = 8/817 (0.1%).				Weak
			Of these cases, URTI = 19/295 (6.4%), **LRTI** = 250/295 (84.7%).	**1-5yo:** MP = 75/616 (12.2%), CP = 30/616 (4.9%), co-infxn = 5/616 (0.1%).				
		**Bacteria:** PCR on NPA, Blood for serology.	LRTI cases: MP = 85.9% (171/199), CP = 81.5% (66/81).	**>5yo:** MP 44/165 (26.7%), CP 11/165 (6.7%), co-infxn 2/165 (0.1%).				
Oumei 2018 [64]	China	**Viruses:** DFA on OPS.	MP = 486 (32.4%).	**6mo-1year** (n = 212): RSV = 62 (4.13), ADV = 13 (0.87), IVA = 7 (0.47), IVB = 7 (0.47), PIV1 = 7 (0.47), PIV2 = 6 (0.40), PIV3 = 9 (0.60), MP = 82 (5.47), Other = 61 (4.07).				Weak
				**1-3years** (n = 502) RSV = 63 (4.20), ADV = 30 (2.00), IVA = 23 (1.53), IVB = 18 (1.20), PIV1 = 21 (1.40), PIV2 = 24 (1.60), PIV3 = 22 (1.50), HMPV = 4 (0.27), MP = 198 (13.20).				
		**Bacteria:** Serum serology.	One viral pathogen = 291 (33.5%); RSV = 173 (11.5%); ADV = 75 (5%); IVA = 61 (4.1%); IVB = 51 (3.4%); PIV1 = 44 (2.9%); PIV2 = 47 (3.1%); PIV3 = 47 (3.1%); HMPV = 5 (0.3%).	**3-5years** (n = 455) RSV 31 (2.07), ADV 24 (1.60), IVA 17 (1.13), IVB 13 (0.87), PIV1 12 (0.80), PIV2 10 (0.67), PIV3 11 (0.73), HMPV 1 (0.07), MP 98 (6.53), Others 314 (20.93).				
			Negative cases = 809 (53.9%).	**5-14years** (n = 331) RSV = 17 (1.13), ADV = 8 (0.53), IVA 14 (0.93), IVB = 13 (0.87), PIV1 = 4 (0.27), PIV2 = 7 (0.47), PIV3 = 5 (0.30), MP = 108 (7.20), Others = 205 (13.67).				
Jiang 2017 [61]	China	**Viruses:** PCR and DFA on NPA.	≥1 respiratory pathogen = 70.1% (593/846): RSV = (22.9%), HRV = (22.1%), MP (15.8%), BV = (6.0%), PIV = (4.0%) and SP = (3.0%).	Positive: 70.7% < 6 mo old, 76.1% 6-11mo, 70.2% 1-<3yo, 74.0% 3-<5yo, 78.0% ≥ 5yo.				Moderate
		**Bacteria:** Culture on blood, pleural fluid, BAL; serum serology.	Co-infection identified = 34.6% (293/846) – mixed viral-bacterial infections = 209 (71.3%). Mixed viral-viral infections = 56 (19.1%) patients, mixed bacterial-bacterial = 28 (9.6%).	RSV (24.6% vs 3%, *P* < 0.01) more common children <5 y old; MP (42.4% vs 13.6%, *P* < 0.01) more common in children ≥5 y old.				
EURO WHO REGION								
UPPER-MIDDLE INCOME COUNTRIES								
Aykac 2018 [53]	Turkey	**Viruses:** PCR on NPS.	**LRTI group** = 264/1240 (21.3%) samples analysed or 264/339 (77.9%) positive samples. RSV = 64 (18.8%), RV = 44, Multiple = 46, PIV = 32, INF = 29, AdV = 17, CoV = 11.	**<1 y** = 186/339: RSV = 56, RV = 28, PIV 32, INF = 14, AdV = 12, CoV = 7.				Weak
			Positive blood cultures = 18/192 (9.3%): KP = 3, SH = 3, SP = 2, SE = 2.	**1-2 y** = 44/339: RSV = 8, RV = 9, PIV = 9, INF = 3, AdV = 4, CoV = 0.				
		**Bacteria:** Blood culture.	**URTI group:** RSV = 9, RV = 14, PIV = 12, INF = 8, AdV = 3, CoV = 4.	**2-5 y** = 56/339: RSV = 7, RV = 12, PIV = 2, INF = 10, AdV = 3, CoV = 4.				
			7/339 (2%) died – AdV = 2, CoV = 1, multiple viruses = 1, INF = 1, RV = 1, HMPV = 1.	**>5 y** = 53/339: RSV = 3, RV = 13, PIV = 3, INF = 10, AdV = 1, CoV = 5.				
				7 died: <1 yo = 3, >5 y of age = 3				
PAHO WHO REGION								
UPPER-MIDDLE INCOME COUNTRIES								
Jonnalagadda 2017 [62]	Ecuador	**Viruses:** PCR on NPS.	RSV = 159 (39.2%), HMPV = 71 (17.5%), AdV = 62 (15.3%), PIV = 57 (14.0%), INF = 40 (9.9%), SP = 37/403 (9.2%), MP = 3 (0.74%)	<1yo = 238: RSV = 105 (44.1%), HMPV = 40 (16.8%), AdV = 35 (14.7%), PIV = 40 (17%), INF = 33 (13.9%), SP = 20 (8.5%), MP = 0 (0%).				Moderate
		**Bacteria:** Blood PCR.		1-5yo = 168: RSV = 54 (32.1%), HMPV = 31 (18.5%), AdV = 27 (16.1%), PIV = 17 (10%), INF = 17 (10%), SP = 17 (10.1%), MP = 3 (1.8%).				
SEARO WHO REGION								
LOWER-MIDDLE INCOME COUNTRIES								
Chisti 2014 [55]	Bangladesh	**Bacteria:** Blood culture, Xpert MTB/RIF, MC&S on gastric lavage and IS	4% blood culture positive = 18/405 – SP = 4, KP = 2, HI = 2, ST = 2, AB = 2, SA = 1, SalmE = 1, Ps spps = 1, Ent spps = 1, Polymicrobial = 2.	Died in hospital = 9% (35/405) ; died at home after discharge = 9% (32/369).				Weak
			TB positive overall = 6.8% (27/396) - culture = 10/396 (3%); Xpert = 21/214 (10%).					
AFRO WHO REGION								
LOW-INCOME COUNTRIES								
Graham 2011 [58]	Malawi	**Viruses**: IFA on NPA.	Confirmed bacterial pneumonia = 58: SP = 34, ST = 10, Hib = 8, SA = 4, EC = 2, KP 1, PCP 16, MTB = 10, Unknown = 243. Lung aspirate culture positive = 2/54.	Overall case-fatality rate = 10.1%. Died with confirmed bacterial pneumonia = 2/56 (4%), Died with PCP = 11/15 (73%).				Moderate
		**Bacteria:** Blood/ Lung aspirate culture and PCR.						
UPPER-MIDDLE INCOME COUNTRIES								
Morrow 2014 [63]	South Africa	**Viruses:** PCR on NPA, Viral shell vial culture & rapid viral Ag on blood; Fungi: PCP DFA on NPA/IS/BAL	PCP = 109/202 (54.0%); CMV = 124/202 (61.4%); Other viruses = 70/202 (34.7%); Bacteraemia = 20/202 (9.9%). In-hospital mortality was 35 (32.1%) in children with PCP compared to 16 (17.2%) in those without PCP (RR = 1.87; 95% CI = 1.11-3.15; *P* = 0.02). Only HIV infection was predictive of mortality (OR = 3.7, 95% CI = 1.5-9.0; *P* = 0.004).			Moderate		
MIXED WHO REGIONS AND INCOME CLASSIFICATIONS								
GABRIEL NETWORK								
Dananche 2018 [56]	Cambodia, China, Mongolia, India, Madagascar, Mali, Haiti, Paraguay	**Viruses:** rt-PCR on NPS/NPA, whole blood, pleural effusion.	**Viruses in respiratory samples** = 888: INF = 86 (9.7%), AdV = 68 (7.7%), BV = 82 (9.2%), CoVNL63 = 10 (1.1%), CoV229E = 7 (0.8%), CoVOC43 = 20 (2.2%), CoVHKU = 23 (2.6%), EV = 42 (4.7%), HMPV = 76 (8.6%), PIV1 = 26 (2.9%), PIV2 = 4 (0.5%), PIV3 = 57 (6.4%), PIV4 = 21 (2.4%), PaV = 21 (2.4%), RSV = 178 (20.0%), RV = 221 (24.9%).		Death in influenza positive = 3/80 (3.8%), Death overall = 21/850 (2.5%)		Weak	
		**Bacteria:** rt-PCR on NPS/NPA, blood culture, pleural effusion culture.	**Bacteria in respiratory samples** = 888: SP = 605 (68%), SA = 107 (12.0%), HI = 47 (5.3%), MP = 13 (1.5%), CP = 4 (0.5%), Viral and bacterial co-colonization = 529 (59.6%).					
			**Blood culture** positive = 24/888 (2.7%), RT-PCR positive for S. aureus = 13/711 (1.8%), RT-PCR positive for SP = 74/711 (10.4%), RT-PCR positive for HI = 24/711 (3.4%).					

Viruses: INFA – influenza A, INFB – influenza B, PIV – parainfluenza virus, AdV – adenovirus, RSV – respiratory syncytial virus, CoV – coronavirus, EV – enterovirus, RV – rhinovirus, BV – bocavirus, HMPV – Human metapneumovirus, PaV – Parechovirus; bacteria: *SH – Staphylococcus hominis, SP - Streptococcus pneumoniae, SE - Staphylococcus epidermidis, KP - Klebsiella pneumoniae, MP - Mycoplasma pneumoniae, CP - Chlamydophila pneumoniae, LP – L. pneumophila, HI – Haemophilus influenzae, ST – Salmonella typhi, AB – Acinetobacter, SA – Staphylococcus aureus, SalmE – Salmonella enteritidis, PS – Pseudomonas* species, Ent – *Enterobacter* species;

Fungi: PCP – *Pneumocystis* pneumonia; DFA – direct immunofluorescence assay, IFA – indirect immunofluorescence assay; IS – induced sputum, BAL – bronchoalveolar lavage, NPA – nasopharyngeal aspirate, NPS – nasopharyngeal swab, OPS – oropharyngeal swab, y – year, yo – year old, mo – months

Three studies from China [54,61,64] and one from Vietnam [60], included children up to 15 years of age and utilised nasopharyngeal swabs for viruses and serology testing for atypical bacteria (*M. pneumoniae, Chlamydia pneumoniae*). Neither of these countries had PCV as part of their routine vaccination programme. In China, atypical pathogens were more commonly detected in children ≥5 years old (MP = 26.7%-42.4%, CP = 6.7%) compared with younger children (MP = 5.5%-13.6%, CP = 4.9%), whereas viruses such as RSV were more commonly detected in younger children (4%-24.6%) vs older children (1%-3%). The study in Vietnam [60] identified the highest rate of severe atypical pneumonia in hospitalised children <2 years of age, which differed from other studies. Those with severe pneumonia were also more likely to be co-infected with other bacterial pathogens (predominantly pneumococcus) or respiratory viruses than the non-severe group.

Jiang et al. [61] focused on co-infections in children 1 month to 14 years of age, with CAP admitted to a tertiary hospital in China. Of 293 cases, 71.3% were mixed viral-bacterial infections, 19.1% mixed viral-viral infections, and 9.6% mixed bacterial-bacterial infections. Young age (<6 months) and admission to a paediatric intensive care unit (PICU) were associated with co-infections [61].

Two studies [58,63] were conducted in countries with high rates of HIV (Malawi and South Africa). One study focused on causes of severe/very severe pneumonia and detected bacteria in 18% of cases (predominantly *S. pneumoniae* and *S. typhimurium*), as well as PJP in 16 cases and TB in 10 cases [58]. The second study described the incidence of PJP (over 50% of cases), which was predominantly diagnosed in HIV-infected individuals. In addition, 61% had CMV, while only five patients were diagnosed with TB [63]. Neither country had introduced PCV at the time of the studies.

A study from Bangladesh [55], which enrolled severely malnourished (z score weight for height<-3 or z score weight for age<-4 or nutritional oedema) children <5 years with radiological pneumonia, explored different diagnostics and specimens for TB diagnosis. Induced sputum culture was positive in 2.5% (n = 10/394) of cases, while gastric lavage culture was positive in 1.5% (n = 6) cases. The yield from Xpert MTB/RIF was higher from both induced sputum (n = 16, 7.6%) and gastric lavage (n = 11, 5.1%). In addition, 4% of blood cultures were positive [55]. Of the 12 studies, four were deemed of moderate quality and eight weak as rated by the EPHPP Quality Assessment Tool.

## DISCUSSION

This systematic review identified the main aetiological agents associated with childhood pneumonia in LMICs in the era of widespread routine PCV and Hib vaccine use. A limited number of pathogens, including RSV, HMPV, influenza, parainfluenza, *S. pneumoniae*, *H. influenzae, S. aureus, M. pneumoniae* and *M. tuberculosis*, accounted for most pneumonia cases in most regions, even though case definitions and detection methods varied between studies and settings. PCV coverage, age, severity of disease, medical conditions and regional differences need to be considered in the interpretation of aetiological results and treatment of pneumonia.

Pathogens appear to vary by region and between high and low mortality settings. AFRO region studies generally showed a predominance of bacterial pathogens. SEARO/WPRO countries proportionally demonstrated more viruses, while WPRO countries such as China, showed atypical bacteria to be important in older children. Although some of these differences may be real variations, they are also likely a function of variable diagnostic capacity, difference in laboratory quality and standards and difference in routine testing.

Studies which described disease by severity showed higher bacterial detection in severe cases compared with non-severe cases. This included complicated disease, such as empyema, (*S. pneumoniae, H. influenzae*, and *S. aureus*) and post-mortem studies. *M. tuberculosis* was also detected when appropriate testing was done as a primary cause of death or, to a lesser extent, as a comorbid condition. RSV was found to be important in hospitalised infants who died and, in studies published subsequent to the review, in children who died outside a health facility [65-68]. This review was undertaken before COVID-19 pneumonia data in children was reported. However, subsequently, a South African study conducted during the peak of the first wave of the COVID-19 outbreak identified histopathology lung findings in 11 cases in which COVID-19 was considered to have contributed to the child’s death [69].

In mild and moderate disease, viruses were the predominant cause of ALRI requiring hospital admission in young children. The most common virus causing severe disease was RSV, especially in children <2 years of age. Influenza and atypical bacteria (*C. pneumoniae* and *M. pneumoniae*) were more common in older children compared with younger children. Severe disease is usually attributed to bacteria as a single pathogen; however, it can also often come from a viral infection followed or accompanied by a bacterial infection, especially in susceptible hosts.

Respiratory tract co-infections are complex and dependent on multiple factors, including the different pathogens involved. Numerous studies in the review had limited bacterial testing and did not report on co-infections. Additionally, the Integrated Analysis model used in PERCH assumed that each pneumonia case was caused by a primary pathogen [3].

Children colonised with pneumococci who are co-infected with respiratory viruses tend to have high nasopharyngeal pneumococcal density [70-72]. Higher pneumococcal colonisation density has also been associated with severe pneumonia [73]. However, a recent study from Israel during COVID-19, when there was no RSV circulating due to public health measures, found that pneumonia admission rates in children declined but pneumococcal density remained unchanged throughout the same period. This suggests that pneumococcal density has less of a a role in pneumonia severity but RSV (and other viruses) may play a more prominent role in disease progession and severity [74].

### Public health strategies

Targeting high risk populations is a common public health prevention strategy. Children and infants living with HIV are known to be at increased risk of incidence and mortality from pneumonia. This increased risk is evident across all common infectious causes of pneumonia (ie, bacteria, viruses and TB), but also includes opportunistic pathogens such as *P. jirovecii* and cytomegalovirus [75,76]. ALRI co-infections in HIV-infected children are common. The epidemiology of ALRI in HIV-infected children has changed since the introduction of strategies to reduce mother-to-child HIV transmission, early anti-retroviral therapy and routine cotrimoxazole preventive treatment [75]. Results in HIV-infected children with radiologically confirmed pneumonia from two PERCH sites [75,76] reported the highest aetiological fraction for *P. jirovecii*, *S. pneumoniae*, *S. aureus* (in both), *M. tuberculosis* (in Zambia) [76], and RSV (in South Africa) [75]. CMV was not an important contributor to the burden of disease [75,76]. Empirical treatment for HIV-infected children should include coverage for common and opportunistic pathogens, although uncertainty remains about the pathogenicity of CMV and the empirical treatment’s effectiveness [77].

There is a synergistic relationship between malnutrition and infection [78]. Malnutrition is associated with a change in the pattern of colonising organisms and variations in normal intestinal function with associated malabsorption, inflammation, changes in metabolism, and leakage of bacteria. Malnutrition compromises mucosal epithelial barriers in the gastrointestinal and respiratory tracts, reducing the first line of defense against infections [78]. Children with malnutrition have high rates of bacterial pneumonia and TB and are more likely to be admitted to hospital with bacterial pneumonia [3]. Severely malnourished children often have an atypical pneumonia presentation and are unable to cough effectively. Malnutrition has also been shown to be associated with a higher risk of mortality amongst pneumonia cases [79,80]. Despite this, there were very few studies on the aetiology of pneumonia in malnourished children. More research is needed to address questions on changes in nutritional status and immune competence during and after infection events.

Many studies in the review did not include testing for *M.tuberculosis*. When tested for, TB was found to be a frequent primary cause of pneumonia or comorbidity in children, especially in cases with empyema. In high TB prevalence settings, children are often initiated on TB therapy without a microbiological diagnosis. Confirming the diagnosing of TB is challenging in young children with sputum culture of a 50% sensitivity at best; clinicians often rely on contact history, non-specific symptoms, and radiological evidence. However, TB is often associated with mortality in children with severe pneumonia, and so early treatment is critical [81].

Viral pathogens are an important cause of pneumonia disease burden across all LMICs, and access to supportive measures such as oxygen and ventilation should be made a priority for severe cases. With the ongoing COVID-19 pandemic, acute respiratory infection with SARS-CoV-2 is generally mild in children, whilst post-infectious outcomes may be more complicated. More research is needed, especially in LMICs [82]. The development and rollout of an effective RSV vaccine would play a major role in preventing childhood pneumonia. In addition, the burden of bacterial disease is higher in populations that are not vaccinated. Systems should be strengthened to provide equitable and universal access to vaccination against important causes of severe pneumonia in children.

### Identification of pneumonia

Even within the PERCH Network, severity of disease varied greatly between sites. Since the review, a number of manuscripts from individual PERCH sites have described site findings [75,76,79,80,83-87]. Variation in disease severity by PERCH site was likely due to several factors. First, high and low mortality settings differ inherently from one another regarding HIV infection and other comorbid infectious disease rates, access to care, and vaccine programmes; second, the inclusion of wheezing, often associated with chest indrawing, even in non-severe cases, varied between sites; and lastly, the heterogeneity in bacterial case definition was complex and relied on carriage data [88,89].

There are many challenges with different biological specimens and diagnostic methods used to determine the aetiology of pneumonia [90], especially for bacteria. Lung tissue is ideal, but impractical. Bacteria are an important cause of severe pneumonia, but blood cultures, considered the gold standard, have low diagnostic sensitivity (10%-15%). PCR techniques may improve the detection of pneumococcal bacteraemia, including in cases with pre-existing antibiotic treatment. *S. pneumoniae* and *H. influenzae* may be detected with culture or PCR in samples from pleural fluid [91,92]. However, the detection of *S. pneumoniae* by PCR (*lytA*) in culture negative blood [93,94] and lung aspirate [24] specimens is not universally regarded as diagnostic of pneumococcal pneumonia in children, as detection by PCR may reflect carriage rather than disease. Nasopharyngeal aspirate can be used to detect *M.tuberculosis,* especially given the increasing availability of Xpert MTB/RIF [95]. In children with respiratory distress, the use of sampling such as nasopharyngeal aspirate or stool has advantages over more invasive sampling such as induced sputum or gastric aspirate [96]. Serology based tests for atypical organisms are unreliable for determining aetiology; they lack specificity and are more useful with paired convalescent serology. Upper respiratory tract samples do not necessarily reflect the organisms in the lower airways or lungs, especially for bacteria as colonisation is common [97]. Lastly, some LMICs have limited access to RT-PCR testing for viruses.

### Recommendations for antibiotic treatment

The current WHO guidelines for the treatment of pneumonia in children include clear indications for the use of antibiotics [47,98]. Based on the available epidemiological data included in this review, treatment for community acquired pneumonia should target *S. pneumoniae* and *H. influenzae* with oral amoxicillin. *H. influenzae* susceptibility may be variable, however given low rate of identification in the review, amoxicillin remains acceptable. For severe community acquired pneumonia, parenteral amoxicillin (or penicillin G) and gentamicin are appropriate as per current guidelines. If there is no or poor response to treatment or any signs of *S. aureus* infection (empyema, pneumatoceles, cellulitis, osteomyelitis), treatment should include parenteral flucloxacillin and gentamicin. In children 5-14 years, providers should consider adding a macrolide if atypical pathogens are suspected or confirmed.

Oseltamivir for influenza may be important in older children with pneumonitis or other signs of severe influenza. In lower risk children, studies have reported variable rates of effectiveness across different respiratory outcomes [99,100]. For severe community-acquired pneumonia with hypoxaemia or para-pneumonic effusion or empyema, treatment should target *S. pneumoniae, S. aureus*, and *H. influenzae* with parenteral flucloxacillin and gentamicin, or parenteral flucloxacillin and ceftriaxone.

In children with HIV, treatment should include antibiotics as described for community-acquired pneumonia, plus anti-TB treatment if there are supportive features such as recent contact or poor response to antibiotics. In addition, treatment for opportunistic pathogens such as *P.jirovecii* or CMV is considered for HIV-infected infants with severe pneumonia.

Local epidemiology and susceptibility patterns should guide second line therapy. In settings where methicillin-resistant *S. aureus* (MRSA) is common, among high-risk populations with evidence of *S. aureus* pneumonia (pneumatocoeles, associated soft-tissue, bone and joint infection), treatment should include vancomycin or another agent against MRSA.

### Limitations

There were several limitations identified in the included studies. First, the high variability in testing strategies and methodologies makes it difficult to compare findings across studies. Due to the heterogeneity between sites, the ability to pool results was limited. Second, case definitions for pneumonia, including those of severity, varied across studies; there was, however, some similarity in the main pathogens identified. Third, studies tested for different pathogens. For example, atypical bacteria were mainly included in studies from the WPRO region, while pleural effusion studies generally only tested for bacteria, actively excluding TB-associated pleural effusions. Fourthly, many studies had no control group, which is important when attributing cause to viral pathogens. Fifthly, while *S. pneumoniae* was still a common bacteria detected in the era of PCV use, studies did not aim to demonstrate the impact of PCV vaccination on aetiology and need to be interpreted in the context of PCV vaccination coverage. Lastly, studies used variable, often broad age groups, yet aetiology is age-related. Overall further research is needed and possible applications to policy and antibiotic selection in childhood pneumonia should be ultimately guided by local health care systems, stakeholders, and resources.

## CONCLUSIONS

We identified that a number of pathogens, including RSV, influenza, human metapneumovirus, *S. pneumoniae*, *H. influenzae, S. aureus*, and *M.tuberculosis*, as important targets for prevention and treatment of childhood ALRI in LMICs. Bacterial pathogens are still responsible for a large proportion of severe or complicated pneumonia, but vaccines against RSV are likely to play a large role in preventing pneumonia. Future research should focus on strengthening the context-specific diagnostic facility capacities for improving local knowledge of viral and bacterial pneumonia aetiology, including identification of pneumonia severity in children. Future studies should include a consistent case definition (eg, WHO pneumonia case definitions), distinguish pneumonia from bronchiolitis where possible, and disaggregate data according to age, as well as clinical and epidemiological risk factors. In addition, an increased emphasis on research that includes very severe and fatal pneumonia in more settings is advisable, especially as we start to monitor replacement in countries using PCV.

## Additional material


Online Supplementary Document
