# Supplementary material for: Aetiology of childhood pneumonia in low- and middle-income countries in the era of vaccination: a systematic review
Source: J Glob Health. 2022 Jul 23;12:10009. doi: 10.7189/jogh.12.10009 (PMC9305023; doi:10.7189/jogh.12.10009)
Supplement: Online Supplementary Document [file jogh-12-10009-s001.pdf]

Online supplementary materials

APPENDIX S1

MEDLINE aetiology search

1. exp *Pneumonia/

2. ((respiratory adj3 infection*) or pneumonia or pneumonias or lung-inflammation* or lobitis or nonspecific-inflammatory-lung-disease* or peripneumonia or pleuropneumonia or pleuropneumonitis or pneumonic-lung* or pneumonic-pleurisy or pneumonic-pleuritis or pneumonitides or pneumonitis or pulmonal-inflammation* or pulmonary-inflammation* or pulmonic-inflammation* or bronchiolitis).tw,kf.

3. *Pneumococcal Infections/

4. *Bacteremia/

5. exp *empyema, pleural/ or exp *pleural effusion/

6. *Streptococcus pneumoniae/

7. exp *Haemophilus/

8. (bacter?emia or empyema or pleural or bronchial or bronchoalveolar or alveolar or endotracheal or tracheal).tw,kf.

9. (pneumoniae or pneumococc* or haemophilus).tw,kf,hw.

10. (4 or 5 or 8) and (6 or 7 or 9)

11. 1 or 2 or 3 or 10

12. (cause* or etiolog* or aetiolog*).tw,kf.

13. et.fs.

14. 12 or 13

15. exp *mortality/

16. *Death/ or *infant death/

17. mo.fs.

18. (autops* or postmortem or post-mortem or mortalit* or death* or surviv* or fatal* or failure or failed or poor-outcome*).tw,kf.

19. 15 or 16 or 17 or 18

20. (x-ray or xray or imaging or radiolog* or radiogra*).tw,kf,hw.

21. (ip or mi or vi).fs.

22. diagnostic*.tw,kf.

23. *nasopharynx/mi, vi or exp *oropharynx/mi, vi

24. (lung or blood or serolog* or sputum or culture* or specimen* or PCR or polymerase-chain-reaction* or pleural-fluid* or NP-OP or swab* or nasopharyngeal-and-oropharyngeal or naso-pharyngeal-and-oro-pharyngeal or nasopharyngeal-oropharyngeal or naso-pharyngeal-oro-pharyngeal).tw,kf,hw.

25. 20 or 21 or 22 or 23 or 24

26. (bacteri* or viral or pathogen* or organism* or pneumoniae or aureus or bordatella-pertussis or b-pertussis or respiratory-syncytial-virus or influenza-virus or bocavirus or adenovirus or parainfluenza-virus or influenzae or cytomegalovirus).tw,kf.

27. *pneumovirus/ or *respiratory syncytial viruses/ or *respiratory syncytial virus, human/

28. *Bordetella pertussis/

29. *Streptococcus pneumoniae/

30. exp *Staphylococcus aureus/

31. exp *Haemophilus influenzae/

32. 26 or 27 or 28 or 29 or 30 or 31

33. exp World Health Organization/

34. World Health Organization.tw,kf.

35. developing countries/

36. (austere or (limited adj2 resource*) or (low adj2 resource*) or (transitioning adj econom*) or (third adj world) or LMIC or LMICs or (lami adj countr*) or (transitional adj countr*) or (low adj gdp) or (low adj gnp) or (low adj gross adj domestic) or (low adj gross adj national) or ((emerging or developing or (low adj income) or (middle adj income) or (low adj3 middle) or underdeveloped or under-developed or (less* adj developed) or underserved or under-served or deprived or poor*) and (countr* or nation*1 or econom* or population or world))).tw,kf.

37. exp africa/

38. americas/ or exp caribbean region/ or exp central america/ or latin america/ or mexico/ or exp south america/

39. europe/ or exp europe, eastern/ or exp transcaucasia/

40. antarctic regions/ or exp atlantic islands/ or exp indian ocean islands/ or exp pacific islands/

41. New Guinea/

42. asia/ or exp asia, central/ or asia, southeastern/ or borneo/ or cambodia/ or east timor/ or indonesia/ or laos/ or malaysia/ or mekong valley/ or myanmar/ or philippines/ or thailand/ or vietnam/ or asia, western/ or bangladesh/ or bhutan/ or india/ or middle east/ or afghanistan/ or iran/ or iraq/ or jordan/ or lebanon/ or oman/ or saudi arabia/ or syria/ or turkey/ or yemen/ or nepal/ or pakistan/ or sri lanka/ or far east/ or china/ or tibet/ or exp korea/ or mongolia/

43. (Afghanistan or Albania or Algeria or Angola or Antigua or Argentina or Armenia* or Aruba or Azerbaijan or Bahrain or Bangladesh or Barbados or Barbuda or Belarus or Byelarus* or Byelorussian or Belorussian or Belorus* or Belize or Benin or Bhutan or Bolivia or Bosnia or Botswana or Brasil or Brazil or Bulgaria or (Burkina adj Fas*) or (Upper adj Volta) or Burma or Burundi or Cambodia or Khmer or Kampuchea or Cameron* or Cameroon* or (Cape adj Verde) or (Cabo adj Verde) or (Central adj African adj Republic) or Chad or Chile or China or Colombia or Comoros or (Comoro adj Island*) or Comores or Mayotte or Congo or Kongo or (Cook adj Island*) or (Costa adj Rica) or (Cote adj D'ivoire) or Croatia or Cuba or Cyprus or Czech* or Djibouti or Dominica or Dominican or (East adj Timor) or (East adj Timur) or Ecuador or Egypt or El-Salvador or (Equatorial adj Guinea) or Eritrea or Estonia or Ethiopia or Fiji or (French adj Somaliland) or Futuna or Gabon or (Gabonese adj Republic) or Gambia or Gaza or (Georgia* adj Republic) or Ghana or Grenada or Guam or Guatemala or Guinea or Guiana or Guyana or Haiti or Herzeg* or Hercegovina or Honduras or Hungary or India or Indonesia or Iran or Iraq or (Ivory adj Coast) or Jamaica or Jordan or Kazakh* or Kenya or Kiribati or Korea or Kosovo or (Kyrgyz adj Republic) or Kyrgyzstan or Kirghizia or Kirghiz or Kirgizstan or Laos or (Lao* adj2 Democratic adj Republic) or (Lao* adj PDR) or Latvia or Lebanon or Lesotho or Basutoland or Liberia or Libya or Lithuania or Macedonia or Madagascar or (Magalasy adj Republic) or Malawi or Malay* or Sabah or Sarawak or Maldives or Mali or (Marshall adj Island*) or Mauritania or Mauritius or (Agalega adj Island*) or Mexico or Micronesia or Moldov* or Mongolia or Montserrat or Montenegro or Morocco or Ifni or Mozambique or Myanma* or

Namibia or Nauru or Nepal or (Netherlands adj Antilles) or (Dutch adj Antilles) or (New adj Guinea) or (New adj Caledonia) or Nicaragua or Niue or Niger or Nigeria or (Northern adj Mariana adj Island*) or Nyasaland or Oman or Pakistan or Palau or Panama or (Papua adj New adj Guinea) or PNG or Palestine or Paraguay or Peru or Philipines or Philippines or Phillipines or Phillippines or Poland or (Puerto adj Rico) or Yemen or Romania or Roumania or Rumania or Russia* or Rwanda or Ruanda or (Saint adj Kitts) or (St adj Kitts) or Nevis or (Saint adj Vincent) or (St adj Vincent) or Grenadines or Samoa* or (Navigator adj Island*) or (Saint adj Lucia) or (St adj Lucia) or (Saint adj Helena) or (St adj Helena) or (Sao adj Tome) or (Saudi adj Arabia) or Senegal or Serbia or Seychelles or (Sierra adj Leone) or Slovenia or Slovak* or (South adj Africa) or (Solomon adj Island*) or Somalia or (Sri adj Lanka) or Ceylon or Sudan or Surinam* or Swaziland or Syria or Tajikistan or Tadzhikistan or Tadjikistan or Tadzhik or Tanzania or Thailand or Tibet or Timor-Leste or Togo or (Togolese adj Republic) or Tokelau or Tonga or Trinidad or Tobago or Tunisia or Turkey or Turkmenistan or Turkmen or Tuvalu or Uganda or Ukraine or Uruguay or Urundi or USSR or (Soviet adj Union) or "Union of Soviet Socialist Republics" or Uzbekistan or Vanuatu or (New adj Hebrides) or Venezuela or Vietnam or (Viet adj Nam) or (Wallis adj2 Futuna) or (United adj Arab adj Republic) or (West adj Bank) or (West adj Indies) or Yemen or Yugoslavia or Zaire or Zambia or Zimbabwe or Rhodesia).tw,kf.

44. (africa or americas or caribbean or (central adj America) or (latin adj America) or (south adj America) or (eastern adj Europe) or Transcaucasia or antarctic or (atlantic adj island*) or (indian adj ocean adj island*) or (pacific adj island*) or polynesia or (central adj asia) or (southeast* adj asia) or (south-east* adj asia) or borneo or mekong or (western adj asia) or (middle adj east) or (far adj east)).tw,kf.

45. 33 or 34 or 35 or 36 or 37 or 38 or 39 or 40 or 41 or 42 or 43 or 44

46. (infan* or toddler* or pre-schooler* or preschooler* or kinder or kinders or kindergarten* or kinder-aged or boy or boys or girl or girls or child or children or childhood or youngster* or kid or kids or pediatric* or paediatric* or school-age* or schoolage* or schoolchild* or schoolgirl* or schoolboy*).af.

47. 11 and (14 or 19) and 25 and 32 and 45 and 46

48. limit 47 to (english language and yr="2010 -Current")

49. limit 48 to (case reports or comment or editorial or guideline or letter or practice guideline)

50. 48 not 49

<u>PubMed aetiology search</u>

#1 Title/Abstract

(“respiratory” AND “infection*”) OR “pneumonia” OR “pneumonias” OR “lung-inflammation*” OR “lobitis” OR “nonspecific-inflammatory-lung-disease*” OR “peripneumonia” OR “pleuropneumonia” OR “pleuropneumonitis” OR “pneumonic-lung*” OR “pneumonic-pleurisy” OR “pneumonic-pleuritis” OR “pneumonitides” OR “pneumonitis” OR “pulmonal-inflammation*” OR “pulmonary-inflammation*” OR “pulmonic-inflammation*” OR “bronchiolitis” OR “pneumococcal-infection*” OR “pneumococcus-infection*” OR “pneumococcal-disease*” OR “parapneumonic” OR ((“bacteremia” OR “bacteraemia” OR “empyema” OR “pleural” OR “bronchial” OR “bronchoalveolar” OR “alveolar” OR “endotracheal” OR “trachael”) AND (“pneumoniae” OR “pneumococc*” OR “haemophilus”))

#2 Title/Abstract

“cause*” OR “etiolog*” OR “aetiolog*” OR “autops*” OR “postmortem” OR “post-mortem” OR “mortalit*” OR “death*” OR “surviv*” OR “fatal*” OR “failure” OR “failed” OR “poor-outcome*”

#3 Title/Abstract

“x-ray” OR “xray” OR “imaging” OR “radiolog*” OR “radiogra*” OR “isolation” OR “purification” OR “microbiolog*” OR “virolog*” OR “diagnostic*” OR “lung” OR “blood” OR “serolog*” OR “sputum” OR “culture*” OR “specimen*” OR “PCR” OR “polymerase-chain-reaction*” OR “pleural-fluid*” OR “NP-OP” OR “swab*” OR “nasopharyngeal-and-oropharyngeal” OR “naso-pharyngeal-and-oro-pharyngeal” OR “nasopharyngeal-oropharyngeal” OR “naso-pharyngeal-oro-pharyngeal”
#4 Title/Abstract
“bacteri*” OR “viral” OR “pathogen*” OR “organism*” OR “pneumoniae” OR “aureus” OR “bordatella-pertussis” OR “b-pertussis” OR “respiratory-syncytial-virus” OR “influenza-virus” OR “bocavirus” OR “adenovirus” OR “parainfluenza-virus” OR “influenzae” OR “cytomegalovirus” OR “pneumovirus”
#5 Title/Abstract
“World Health Organization” OR “WHO” OR "austere" OR "limited resource" OR "limited resources" OR "resource limited" OR "low resource" OR "low resource" OR "transitioning economy" OR "transitioning economies" OR "lami country" OR "lami countries" OR "transitional country" OR "transitional countries" OR "low gdp" OR "low gnp" OR "low gross domestic" OR "low gross national" OR "third world" OR "lmic" OR "lmics" OR (("emerging" OR "developing" OR "low income" OR "middle income" OR ("low" AND "middle") OR "underdeveloped" OR "under developed" OR "underserved" OR "under served" OR (“less” AND “developed”) OR "deprived" OR "poor") AND ("country" OR "countries" OR "nation" OR "nations" OR "economy" OR "economies" OR "population" OR "world")) OR "afghanistan" OR "albania" OR "algeria" OR "angola" OR "antigua" OR "argentina" OR "Armenia" OR "aruba" OR "azerbaijan" OR "bahrain" OR "bangladesh" OR "barbados" OR "barbuda" OR "belarus" OR "Byelarus" OR "Belorussia" OR "byelorussia" OR "Belorus" OR "belize" OR "benin" OR "bhutan" OR "bolivia" OR "bosnia" OR "botswana" OR "brasil" OR "brazil" OR "bulgaria" OR "burkina Faso" OR "Upper Volta" OR "burma" OR "burundi" OR "cambodia" OR "khmer" OR "kampuchea" OR "Cameroon" OR "Cape Verde" OR "Cabo Verde" OR "Central African Republic" OR "chad" OR "chile" OR "china" OR "colombia" OR "comoros" OR "comoro Islands" OR "comores" OR "mayotte" OR "congo" OR "kongo" OR "cook Islands" OR "Costa Rica" OR "Cote D'ivoire" OR "croatia" OR "cuba" OR "cyprus" OR "Czechia" OR "Czech Republic" OR "czechoslovakia" OR "djibouti" OR "dominica" OR "East Timor" OR "East Timur" OR "ecuador" OR "egypt" OR "el salvador" OR "Equatorial Guinea" OR "eritrea" OR "estonia" OR "ethiopia" OR "fiji" OR "French Somaliland" OR "futuna" OR "gabon" OR "Gabonese Republic" OR "gambia" OR "gaza" OR (“Georgia*” AND “republic”) OR "ghana" OR "grenada" OR "guam" OR "guatemala" OR "guinea" OR "guiana" OR "guyana" OR "haiti" OR “Herzeg*” OR "hercegovina" OR "honduras" OR "hungary" OR "india" OR "indonesia" OR "iran" OR "iraq" OR "Ivory Coast" OR "jamaica" OR "jordan" OR Kazakh* OR "kenya" OR "kiribati" OR "korea" OR "kosovo" OR "Kyrgyz Republic" OR "kyrgyzstan" OR "kirghizia" OR "kirghiz" OR "kyrgyzstan" OR "Lao" OR "laos" OR "latvia" OR "lebanon" OR "lesotho" OR "basutoland" OR "liberia" OR "libya" OR "lithuania" OR "macedonia" OR "madagascar" OR "Magalasy Republic" OR "malawi" OR Malay* OR "sabah" OR "sarawak" OR "maldives" OR "mali" OR "marshall Islands" OR "mauritania" OR "mauritius" OR "mexico" OR "micronesia" OR "Moldova" OR "mongolia" OR "montserrat" OR "montenegro" OR "morocco" OR "ifni" OR "mozambique" OR Myanma* OR "namibia" OR "nauru" OR "nepal" OR "Netherlands Antilles" OR "Dutch Antilles" OR "New Guinea" OR "New Caledonia" OR "nicaragua" OR "niue" OR "niger" OR "nigeria" OR ("Northern Mariana" AND “Island*”) OR "nyasaland" OR "oman" OR "pakistan" OR "palau" OR "panama" OR "Papua New Guinea" OR "PNG" OR "palestine" OR "paraguay" OR "peru" OR

"philipines" OR "philippines" OR "phillipines" OR "phillippines" OR "poland" OR "Puerto Rico" OR "yemen" OR "romania" OR "roumania" OR "rumania" OR "Russia" OR "rwanda" OR "ruanda" OR "Saint Kitts" OR "St Kitts" OR "nevis" OR "Saint Vincent" OR "St Vincent" OR "grenadines" OR "Samoa" OR "navigator Islands" OR "Saint Lucia" OR "St Lucia" OR "Saint Helena" OR "St Helena" OR "Sao Tome" OR "Saudi Arabia" OR "senegal" OR "serbia" OR "seychelles" OR "Sierra Leone" OR "slovenia" OR "Slovakia" OR "South Africa" OR "solomon Islands" OR "somalia" OR "Sri Lanka" OR "ceylon" OR "sudan" OR "Surinam" OR "suriname" OR "swaziland" OR "syria" OR "tajikistan" OR "tadzhikistan" OR "tadjikistan" OR "tadzhik" OR "tanzania" OR "thailand" OR "tibet" OR "timor leste" OR "togo" OR "Togolese Republic" OR "tokelau" OR "tonga" OR "trinidad" OR "tobago" OR "tunisia" OR "turkey" OR "turkmenistan" OR "turkmen" OR "tuvalu" OR "uganda" OR "ukraine" OR "uruguay" OR "urundi" OR "ussr" OR "Soviet Union" OR "Union of Soviet Socialist Republics" OR "uzbekistan" OR "vanuatu" OR "New Hebrides" OR "venezuela" OR "vietnam" OR "Viet Nam" OR "Wallis" OR "United Arab Republic" OR "West Bank" OR "West Indies" OR "yemen" OR "yugoslavia" OR "zaire" OR "zambia" OR "zimbabwe" OR "rhodesia" OR "africa" OR "americas" OR "caribbean" OR "central America" OR "latin America" OR "south America" OR "eastern Europe" OR "transcaucasia" OR "antarctic" OR "atlantic islands" OR "indian ocean" OR "pacific islands" OR "polynesia" OR "central asia" OR (“southeast*” AND “asia”) OR (“south-east*” AND “asia”) OR "south east asia" OR "south eastern asia" OR "borneo" OR "mekong" OR "western asia" OR "middle east" OR "far east"

#6 Title/Abstract

“infan*” OR “toddler*” OR “pre-schooler*” OR “preschooler*” OR “kinder” OR “kinders” OR “kindergarten*” OR “kinder-aged” OR “boy” OR “boys” OR “girl” OR “girls” OR “child” OR “children” OR “childhood” OR “youngster*” OR “kid” OR “kids” OR “pediatric*” OR “paediatric*” OR “school-age*” OR “schoolage*” OR “schoolchild*” OR “schoolgirl*” OR “schoolboy*”

#7 All fields

NOTNLM OR publisher[sb] OR inprocess[sb] OR pubmednotmedline[sb] OR indatareview[sb] OR pubstatusaheadofprint

#8 #1 AND #2 AND #3 AND #4 AND #5 AND #6 AND #7

Limits: english language; 2010-current; excluded case reports, comments, letters, editorials, guidelines and practice guidelines

Table S1: Characteristics of case-control studies

Lead author and publication date	Country	WHO U5MR/ 1000 live births	PCV introduced at time of study	Hib introduced at time of study	Aim of study	Healthcare level	Study period	Population description/ Eligibility criteria	Case definition for case group	Case definition for comparison group	Total # LRTI participants	Total # comparison participants
AFRO WHO REGION												
LOWER-MIDDLE INCOME COUNTRIES												
Breiman 2015	Kenya	41.1	No	Yes -2001	Compare aetiology & epidemiology from population-based infectious disease surveillance site in Kibera, Nairobi.	Community clinic/ primary care	March 2007 - Feb 2011	Interviewers visited enrolled households every two weeks to identify children who met case definition. Asymptomatic controls enrolled from primary clinic. Each month enrolled up to six controls in each age band and frequency-matched to cases by known HIV status.	Clinic-based SARI case definition in children aged <5 yrs. Modified WHO IMCI algorithm for severe and very severe pneumonia.	Children aged <5 years visiting clinics for non-severe illness, immunisations or medicine refills. No fever, no respiratory symptoms or diarrhoea in the past 2 weeks.	2592 enrolled: 815 with swabs; 836 with blood cultures.	115
Feikin 2013	Kenya	41.1	No	Yes - 2001	Bacterial and viral aetiologies of severe acute respiratory illness (SARI) in children by age group, hospitalization status and season.	District/ Secondary Hospital	March 2007 - Feb 2010	Ongoing population-based, infectious disease surveillance in rural Nyanza Province, Kenya. Community, clinic and hospital surveillance for children <5 years. Asymptomatic controls enrolled from Lwak Hospital.	Met WHO IMCI definitions for severe and very severe pneumonia.	Children visiting clinics for non-severe illness, immunisations or medicine refills. No fever, no respiratory symptoms or diarrhoea in the past 2 weeks.	2973 (20%) SARI patients, 1600 (54%) hospitalised and 1373 (46%) outpatients	93
Hammitt 2012	Kenya	41.1	No	Yes - 2001	Determine aetiology among infants and children with severe pneumonia admitted to a rural Kenyan district hospital. Lessons were applied to the design of the larger multisite PERCH study.	District/ Secondary Hospital	Jan 2010 - Feb 2011	Case patients were children 1-59 months admitted to Kilifi District Hospital (KDH) who had severe pneumonia or very severe pneumonia. Controls were a convenience sample of children attending the outpatient clinic at KDH or at 2 peripheral health centres.	WHO defined severe / very severe pneumonia	Two groups of controls: (1) those with symptoms of an URTI (cough, runny or blocked nose, or sore throat), (2) those without any respiratory infection symptoms or signs.	2606 admitted, 964 (37.0%) met case definition, 810 (84.0%) enrolled.	369

UPPER-MIDDLE INCOME COUNTRIES												
Zar 2016	South Africa	33.8	Yes -2009	Yes -2000	Investigate pneumonia aetiology in a birth cohort study (Drakenstein Child Health Study) using a nested case-control design.	Community clinic/ primary care	May 2012 – Dec 2014	Undertaken at two public, primary health-care clinics in Paarl. Birth cohort followed up and given PHC and routine immunisations. Continuous pneumonia surveillance at clinics and local hospital.<br><br>Nested case-control study included participants aged 0-19 months, who develop pneumonia.	Any episode of WHO-defined non-severe pneumonia or severe pneumonia	Controls were asymptomatic or had URTI symptoms.<br><br>Controls were matched to cases by birth date, age of presentation and site.	967 children in total birth cohort; 284 pneumonia cases enrolled.	719 (74%) in cohort;<br>418 controls in CCS.
SEARO WHO REGION												
LOWER-MIDDLE INCOME COUNTRIES												
Chowdhury 2020	Bangladesh	30.2	Yes - 2015	Yes - 2009	Viral pneumonia aetiology among young children with severe acute malnutrition (SAM) in an urban hospital. Pneumonia outcome according to viral aetiology within 30 days of admission.	Tertiary/ Teaching Hospital	April 2015 - Dec 2017	Severely malnourished children 0-59 months admitted to Dhaka Hospital, International Centre for Diarrhoeal Disease Research Bangladesh.<br>Cases and controls met case definitions, gave written informed consent. Excluded if planning to migrate out of Dhaka within one month.	Cases were severely malnourished and met WHO clinical criteria for pneumonia. Also enrolled children as cases if they had SAM, cough, and radiological pneumonia.	Controls were SAM children with no pneumonia on admission, and no respiratory symptoms and/or signs of pneumonia within the past 10 days prior to admission.	656 screened and 360 enrolled as cases	507 screened and 334 enrolled as controls
UPPER-MIDDLE INCOME COUNTRIES												
Piralam 2020	Thailand	9.1	No	No	Pneumococcal colonization density among young children hospitalized with pneumonia.	District/ Secondary Hospital	January 2012 - February 2014	PERCH enrolment in rural Thailand in 2 study sites (Nakhon Phanom and Sa Kaeo).<br><br>Criteria as per PERCH study (O'Brien 2019).	WHO-defined severe or very severe pneumonia (using pre-2013 definitions)	Healthy community controls or controls with mild RTI	Enrolled 224 severe/ very severe pneumonia cases	659 community controls [254 (38.5%) controls had RTI]
MIXED WHO REGIONS AND INCOME CLASSIFICATIONS												
GABRIEL NETWORK												
Benet 2017	**SEARO:** India (IN);	**LIC** MG 53.6,	**IN** No; **MG** Yes - 2012;	**IN** Yes - 2013;	Microbiological agents linked to hypoxemia in	District/ Secondary Hospital	May 2010 – Jun 2014	Consecutive paediatric hospitalised pneumonia patients enrolled during	WHO clinical pneumonia.	Non-hypoxaemic pneumonia	405 cases - 70 with hypoxemia	335

	**AFRO:** Madagascar (MG), Mali (ML); **PAHO:** Paraguay (PY).	ML 97.8; **LMIC** IN 36.6; **UMIC** PY 20.2	**ML** Yes -2011; **PY** Yes -2012	**MG** Yes - 2008; **ML** Yes - 2005; **PY** Yes - 2003	hospitalized children with pneumonia in developing countries. Identify predictors of hypoxemia and factors associated with death.		(Exact study periods varied across sites)	each season for ≥1-year period.<br>Children aged 2-60 months who met case definitions. Written informed consent.<br>Patients with key missing data excluded.	WHO radiologically confirmed pneumonia.<br><br>Hypoxaemic pneumonia.			
Benet 2017	**WPRO:** Cambodia (KH), China (CN), Mongolia (MN); **SEARO:** India (IN), **AFRO:** Madagascar (MG), Mali (ML); **PAHO:** Haiti (HT), Paraguay (PY).	**LIC:** HT 64.8, MG 53.6, ML 97.8; **LMIC:** KH 28, IN 36.6, MN 16.3; **UMIC** CN 8.6, PY 20.2.	**KH** No; **CN** No; **HT** No; **IN** No; **MG** Yes -2012; **ML** Yes - 2011; **MN** No; **PY** Yes -2012	**KH** Yes - 2010; **CN** No; **HT** Yes - 2013; **IN** Yes - 2013; **MG** Yes – 2008; **ML** Yes - 2005; **MN** Yes - 2005; **PY** Yes - 2003	Assess microorganisms associated with pneumonia in children <5 years old in developing countries	District/ Secondary Hospital	May 2010 – Jun 2014 (Exact study periods varied across sites)	Consecutive paediatric pneumonia patients enrolled during each season for ≥1-year period.<br>Case inclusion criteria: Hospitalised patients 2-60 mos.; Met WHO clinical or radiological pneumonia definitions; First symptoms appearing in last 14 days; Written informed consent.<br>Control inclusion criteria: Aged 2-60 mos.; Met control definition; Written informed consent.	WHO clinical pneumonia.<br>WHO radiologically confirmed pneumonia	No signs/ symptoms of respiratory illness/URTI; hospitalised for surgery or attending routine outpatient appointment at hospital site.	1168 screened and 888 enrolled	870
PERCH NETWORK											
O'Brien 2019	**AFRO:** The Gambia (GM); Mali (ML); Zambia (ZM); South Africa (ZA); Kenya (KE); **SEARO:** Bangladesh (BD); Thailand (TH).	**LIC:** GM 58.4; ML 97.8; **LMIC:** BD 30.2; KE 41.1; ZM 57.8; **UMIC** ZA 33.8; TH 9.1	**GM** Yes - 2010; **ML** Yes - 2011; **ZM** Yes - 2013; **ZA** Yes - 2009; **KE** Yes - 2011; **BD** No; **TH** No.	**GM** Yes - 2000; **ML** Yes -2005; **ZM** Yes - 2004; **ZA** Yes -2000; **KE** Yes - 2002; **BD** Yes - 2009; **TH** No.	Characterise causes of severe childhood pneumonia requiring hospital admission. Reports clinical and microbiological findings of the PERCH study using a traditional analytical approach.	District and Tertiary Hospitals	August 2011 - January 2014	Eligible cases 1-59 months enrolled to one of the nine study locations in seven countries.<br>Controls randomly selected from same catchment area.<br><br>Cases and controls excluded if hospitalised within preceding 14 days, discharged as a PERCH case within preceding 30 days, and residence outside study	Severe pneumonia defined as cough or difficulty breathing with lower chest wall indrawing.<br>Very severe pneumonia defined as cough or difficulty breathing and >=1 danger sign.<br>Elevated respiratory rate not part of case definitions.	Controls were enrolled regardless of respiratory symptoms but were ineligible if they met the PERCH case definition.	5723 eligible children screened, 4232 (73.9%) enrolled.	8994 children contacted; 5530 (61.5%) screened and 5478 (60.9%) eligible.

								catchment area. Cases excluded if lower chest wall indrawing resolved after bronchodilator therapy for children with wheeze.				
Thea 2017	**AFRO:** The Gambia (GM); Mali (ML); Zambia (ZM); South Africa (ZA); Kenya (KE); **SEARO:** Bangladesh (BD); Thailand (TH).	**LIC:** GM 58.4; ML 97.8; **LMIC:** BD 30.2; KE 41.1; ZM 57.8; **UMIC** ZA 33.8; TH 9.1	**GM** Yes - 2010; **ML** Yes - 2011; **ZM** Yes - 2013; **ZA** Yes - 2009; **KE** Yes - 2011; **BD** No; **TH** No.	**GM** Yes - 2000; **ML** Yes -2005; **ZM** Yes - 2004; **ZA** Yes -2000; **KE** Yes - 2002; **BD** Yes - 2009; **TH** No.	Assess prevalence of pathogens in induced sputum (IS) compared with nasopharyngeal/ oropharyngeal specimens. Determine whether presence or quantity of respiratory pathogens detected with PCR in IS specimens was associated with clinical pneumonia status.	Tertiary/ Teaching Hospital	August 2011 - January 2014	Criteria as per PERCH study (O'Brien 2019)	WHO-defined severe and very severe pneumonia<br><br>WHO radiologically-confirmed pneumonia.	Healthy community controls or controls with mild RTI.<br><br>Mock "Non-pneumonia" control group with IS specimens with SP/VSP and normal CXR.	4232 cases (severe 2862 and very severe 1370)	5325 controls

- WHO clinical pneumonia criteria: Cough and/or dyspnea and tachypnea (in children 2-12 months of age: breathing rate >=50 cycles per minute; in children 12-59 months of age: breathing rate>=40 cycles per minute).
- WHO radiological confirmed pneumonia: Primary endpoint pneumonia, other infiltrate, no consolidation/infiltrate/effusion.
- WHO severe pneumonia: cough or difficulty breathing and lower chest-wall indrawing.
- WHO very severe pneumonia: cough or difficulty breathing and any of the following symptoms or signs: unable to drink/breast-feed, vomits everything, convulsions, lethargic or unconscious, stridor when calm, cyanosis or oxygen saturation <90%.
- Mild respiratory tract infection (RTI): cough, runny nose, or at least one of (a) ear discharge, wheezing, or difficulty breathing, and (b) either temperature of >38.0◦C in previous 48 hours or history of sore throat.
- WHO severe malnutrition: <-3 z score from the median of weight for height/length, weight for age, or nutritional oedema.

Table S2: Characteristics of post-mortem studies

Lead Author and publication date	Country	WHO U5MR/ 1000 live births	PCV introduced	Hib introduced	Aim of study	Study design	Healthcare level	Study period	Population description	Inclusion/ Exclusion criteria	Case definition for cases	Total # of participants and final assessment
AFRO WHO REGION												
LOWER-MIDDLE INCOME COUNTRIES												
Bates 2016	Zambia	57.8	Yes – 2013	Yes - 2004	Describe microbiological and histopathological findings from post-mortem lung examination among inpatient children who died. Determine burden of respiratory pathogens.	Prospective cross-sectional autopsy study	Tertiary/ Teaching Hospital	Aug 2011 - June 2014	Inpatient paediatric deaths at National referral centre, University Teaching Hospital, Lusaka, Zambia. Recruited Monday to Friday.	All children aged 0-15 years of age who died in the inpatient wards at UTH were eligible for inclusion in the study.	Histopathologically confirmed pneumonia	1471 screened; 121 consented 111 had lung pathology
UPPER-MIDDLE INCOME COUNTRIES												
Chawana 2019	South Africa	33.8	Yes - 2009	Yes - 2000	Evaluate acceptability and utility of minimally invasive tissue sampling as proof of concept, in ascertaining the causal pathway of death in children in Soweto, South Africa (prelude to CHAMPS program)	Diagnostic test accuracy study	Tertiary/ Teaching Hospital	June 2015 - August 2016	Children who died in any medical, surgical or emergency departments at Chris Hani Baragwaneth Hospital, Soweto. Those certified as dead upon arrival at the hospital. Daily screening of death registries.	All children aged 0-14 years of age who died. Excluded cases in which MITS procedure not performed within 72 hours.	DeCoDe panel for post mortem cause of death attribution	30.8% (127 of 412 deaths) with 32 cases of CAP
MIXED WHO REGIONS AND INCOME CLASSIFICATIONS												
CHAMPS (Child Health and Mortality Prevention Surveillance) NETWORK												
Taylor 2020	**SEARO:** Bangladesh (BD); **AFRO:** Mali (ML); Mozambique (MZ); South Africa (ZA); Kenya (KE).	**LIC:** ML 97.8, MZ 73.2; **LMIC:** BD 30.2, KE 41.1; **UMIC:** ZA 33.8	**ML** Yes - 2011; **MZ** Yes - 2013; **ZA** Yes - 2009; **KE** Yes - 2011; **BD** Yes - 2015;	**ML** Yes -2005; **MZ** Yes -2009; **ZA** Yes -2000; **KE** Yes -2002; **BD** Yes - 2009	Provide data from the first 2 years of the CHAMPS study on all under-5 deaths and stillbirths, using MITS* to obtain detailed, high quality and pathogen specific cause of death data.	Prospective mortality surveillance	Both facility and community recruitment	Dec 2016 - Dec 2018	Autopsy study for deaths from all causes as part of the CHAMPS Network. All deaths notified by primary healthcare facilities and outside facilities.	Children aged <60 months or stillbirths, resided within study catchment area. Notification within 36 h of death (or 72 h if post-mortem refrigeration). Excluded if no consent given.	Stillborn or deceased children <5 years of age from any cause	2385 cases screened; 1430 eligible for MIT; 933 had MIT done (180 still births, 449 neonatal deaths, 304 children's deaths).

Table S3: Characteristics of empyema studies

Lead Author and publication date	Country	WHO U5MR/ 1000 live births	PCV introduced at time of study	Hib introduced at time of study	Aim of study	Study design	Health care level	Study period	Population description	Inclusion/ Exclusion criteria	Case definition for cases	Total # of participants in LRTI group
PAHO WHO REGION												
UPPER-MIDDLE INCOME COUNTRIES												
Feris-Iglesias 2014	Dominican Republic	28.8	No	Yes - 2001	Determine predominant aetiologies causing pleural effusions and estimate proportion of disease that would be prevented through PCV introduction.	Prospective pneumonia surveillance	Tertiary/ Teaching Hospital	Jul 2009 – June 2011	Children <15 years with pneumonia and effusion. Admitted Robert Reid Cabral Children's Hospital, Santo Domingo.	Included: Met study case definition; Excluded: No written informed consent	History of fever/ Temp >=38 °C, tachypnoea (RR>30/min children <8yo and >25/min children 8-16yo), and CXR evidence of pleural effusion requiring thoracocentesis	All 121 enrolled patients had pleural fluid cultured, and 112 (92.6%) had pleural fluid specimens tested by PCR
SEARO WHO REGION												
LOWER-MIDDLE INCOME COUNTRIES												
Dass 2011	India	36.6	No	No	Describe empyema management in tertiary care centre in India wrt clinical characteristics, bacterial aetiology, management, outcome and complications	Cross sectional study	Tertiary/ Teaching Hospital	Jan 2006 – June 2010	All cases <15yo diagnosed with empyema, identified from inpatient records and discharge summaries.	Included patients with aspiration of pus from pleural space. Excluded patients with significant comorbidities and tubercular empyema.	Cases with empyema	160 identified; 150 included in the analysis
UPPER-MIDDLE INCOME COUNTRIES												
Lochindarat 2014	Thailand	9.1	No	No	To determine the frequency of *S. pneumoniae* causing empyema and parapneumonic pleural effusions in children using molecular and standard microbiological techniques.	Prospective observational study	Tertiary/ Teaching Hospital	Jan 2008 – Nov 2009	Hospital-based study at nine centres. Study period at each centre was 12 months to account for seasonal variations. Included children <16 years admitted with empyema or parapneumonic pleural effusions.	Included if met case definition and required drainage of pleural fluid/ thoracocentesis. Excluded if in another clinical trial; pleural effusion d/t malignancy, collagen vascular disease, or melioidosis; previously enrolled and no/inadequate PF sample.	Child admitted with empyema or parapneumonic pleural effusion.	141 children screened, 71 (50%) enrolled (29 empyema, 42 parapneumonic pleural effusions).

AFRO WHO REGION												
LOW-INCOME COUNTRIES												
Howie 2014	The Gambia	58.4	No	Yes - 1998	To elucidate the aetiology of severe pneumonia in children in the Gambia.	Cross sectional study	Tertiary/ Teaching Hospital	2007-2009	All children 2-59 months with CAP recruited from the Medical Research Council hospital in Fajara; Royal Victoria Teaching Hospital in Banjul; and major health centres at Fajikunda, Serekunda, and Brikama.	Included: (1) Met severe pneumonia case definition, (2) Written informed consent; Excluded: Children with cough of >2 weeks, or severe anaemia (Hb<6 g/dL) or confirmed wheeze.	*WHO severe pneumonia definition (modified) **WHO radiological pneumonia	Severe pneumonia cases N=207; Cases with radiological pneumonia N=75; Cases undergoing lung or pleural aspiration N=55.
LOWER-MIDDLE INCOME COUNTRIES												
Kuti 2014	Nigeria	119.9	No	Yes - 2012	To determine the pattern of presentation and the factors that predispose children with pneumonia to developing parapneumonic pleural effusions, as well as the hospital outcome.	Retrospective observational study	Tertiary/ Teaching Hospital	Jan 2011 – Dec 2013	Children aged 1 month - 15 years, admitted for pneumonia in Wesley Guild Hospital, Ilesa. Majority (85.7%) of children with effusions were infants. Case notes retrieved from unit's medical records department.	Included: Meeting study case definition. Excluded: Children with pleural effusions from malignancy, renal pathology, other non-pneumonic causes including tuberculous effusions.	*Clinical pneumonia **Para-pneumonic effusion based on radiological evidence	1470 children admitted; 352 (23.9%) had pneumonia; 28 (8.0%) had para-pneumonic effusions
UPPER-MIDDLE INCOME COUNTRIES												
Zampoli 2015	South Africa	33.8	Yes – 2009 (Cohort A – All unimmunised)	Yes - 2000	To investigate the aetiology of empyema in South African children and estimate the impact of PCV on empyema incidence.	Cohort A Prospective surveillance<br><br>Cohort B retrospective record review	Tertiary/ Teaching Hospital	Dec 2006 – Dec 2014	Children <12 years with empyema recruited at Red Cross War Memorial Children's Hospital, referral hospital in Cape Town, South Africa. Two consecutive cohorts. Cohort A: Direct pulmonology service referral. Cohort B: Retrospective hospital electronic ICD 10-coded	Included children meeting case definition. Excluded pleural effusion after surgery or trauma; children in cohort A presenting with a suspected TB-associated pleural effusion; children in cohort B with probable or culture-confirmed TB pleural effusions.	Children hospitalized with empyema defined as a pleural effusion that after pleural tap was purulent or turbid on inspection or showed neutrophil predominance on cell count.	Cohort A: 142 participants; Cohort B: 81 participants, 59 excluded and 22 (27%) enrolled.

									admissions database search and pulmonology service records for all patients with pleural effusion, empyema or pyothorax.			
Ghoor 2018	South Africa	33.8	Yes - 2009	Yes - 2000	To describe the clinical epidemiology of empyema at a secondary-tertiary hospital with a paediatric pulmonology referral service, in a setting of high HIV and TB prevalence.	Retrospective descriptive study	Tertiary/ Teaching Hospital	Jan 2012 – Dec 2016	Children <14 years diagnosed with empyema at Chris Hani Baragwanath Academic Hospital, Soweto, Johannesburg, South Africa, identified through pulmonology and discharge summary administrative databases.	Cases who met the case definition for empyema were included.<br>Excluded if abscess only, not hospitalised, diagnosed with chylothorax, lung cysts or hydatid cysts.	Doctors diagnosed empyema by visualising purulent material drained from the pleural space or on ultrasound and/or CT scan	154 screened database cases and 65 enrolled
MIXED WHO REGIONS AND INCOME CLASSIFICATIONS												
PERCH NETWORK												
Ebruke 2020	**SEARO**:<br>Bangladesh (BD);<br>**AFRO**:<br>The Gambia (GM), Mali (NL), South Africa (ZA)	**LIC**:<br>GM 58.4, ML 97.8;<br>**LMIC**:<br>BD 30.2;<br>**UMIC**:<br>ZA 33.8	**BD**: No<br>**GM**: Yes - 2010,<br>**ML**: Yes - 2011,<br>**ZA**: Yes – 2009	**BD**: Yes - 2009<br>**GM**: Yes - 1998,<br>**ML**: Yes - 2006,<br>**ZA**: Yes – 2000	Report findings from lung aspirate and pleural fluid specimens collected in the PERCH study	Secondary analysis for cases from case-control study.	Combination of district and tertiary hospitals	Aug 2011 – Jan 2014	Children 1-59 months from four PERCH sites where lung aspiration collection was ethical approved. Screening for cases was done during predefined hours, except for Mali, where a systematic sampling process was used.	Cases eligible for lung aspiration with peripheral confluent alveolar consolidation on CXR, written informed consent, and no contraindications.<br>Only pleural fluid specimens obtained within 3 days of enrolment included in analysis.<br>Excluded children with wheeze whose lower chest wall indrawing resolved with bronchodilator therapy	Peripheral confluent alveolar consolidation and pleural fluid identified on CXR	2757 enrolled in PERCH, 622 (23%) had CXR confluent alveolar consolidation, of whom 48 (8%) had lung aspirate done; 44 (92%) included in analysis

*Clinical pneumonia diagnosis: age-specific tachypnoea, cough, and evidence of respiratory distress, reduced or absent breath sounds, bronchial breath sound or coarse crepitations with or without significant radiological findings to suggest radiologic pneumonia.

**Parapneumonic effusion diagnosis: based on radiological evidence of fluid collection in the pleural space (i.e. obliteration of the costophrenic angles or as a layer of fluid adjacent to the lateral chest wall) with positive free-flowing yield on percutaneous pleural aspiration.

Table S4: Characteristics of surveillance studies

Lead Author and publication date	Country	WHO U5MR/ 1000 live births	PCV introduced at time of study	Hib introduced at time of study	Aim of study	Healthcare level	Study period	Population description	Inclusion/ Exclusion criteria	Case definition for LRTI group	Total # participants in LRTI group and tested
EMRO WHO REGION											
LOWER-MIDDLE INCOME COUNTRIES											
Ali 2016	Pakistan	69.3	Yes - 2013	Yes - 2009	To determine incidence of eight viruses and their subtypes in children aged <2 years old with severe pneumonia in a rural district of Pakistan	Community clinic/ primary care	Oct 2011 - June 2014	Children aged 0-2 years in rural district of Matiari, located in Sindh province, Pakistan. Children enrolled as newborns and followed up fortnightly.	Included: Newborns up to 14 days without major congenital abnormality; parents/guardians informed consent; Excluded: Families planning to move out of study area during 6 months after enrolment.	WHO severe pneumonia case definition (2005)*	817 enrolled, 692 (85%) tested
SEARO WHO REGION											
UPPER-MIDDLE INCOME COUNTRIES											
Olsen 2010	Thailand	9.1	No	No	To present results for first 30 months of active pneumonia surveillance in Thailand, focusing on the incidence of viral and atypical bacterial pathogens.	Two provincial, 16 district and 2 military hospitals	Sep 2003 - Dec 2005	Active, population based surveillance for patients of all ages with pneumonia at 8 hospitals (1 provincial, 6 district and 1 military) in Sa Kaeo province and any of the 12 hospitals (1 provincial, 10 district and 1 military) in Nakhon Phanom province.	Included: Met case definition. Patients who had chest radiograph performed within 48 hrs of admission. Excluded: No written informed consent	ALRI case definition **	15403 admissions, 8351 (54%) had CXRs, 3910 (47%) enrolled (<5yo 1325 enrolled).
Baggett 2012	Thailand	9.1	No	No	To describe incidence and epidemiology of hospitalisations associated with influenza A(H1N1) pdm09 and influenza viruses overall and compare characteristics.	Tertiary/ Teaching Hospital	Jan 2009 – Dec 2010	Patients of all ages admitted with ALRI	Included: ALRI cases as per ALRI definition; Excluded: Admission over weekend/evening when study nurse not available, no patient/guardian informed consent.	ALRI case definition **	7207 enrolled, 2436 children aged <5 years

Naorat 2013	Thailand	9.1	No	No	To describe incidence, epidemiology, and clinical characteristics of ALRI hospitalisations due to RSV among all age groups.	District/ Secondary Hospital	Jan 2008 - Dec 2011	Active population-based surveillance for hospitalised cases of community-acquired ALRI in all 20 hospitals in 2 rural Thailand provinces (Sa Kaeo and Nakhon Phanom). All ages systematically sampled, consented and enrolled.	Included: Met study case definition; Excluded: No written informed consent	ALRI case definition **	<5yo 19,199 hospitalized with ALRI, 4,839 (24%) enrolled.<br><br>5-19yo 7,220 hospitalized, 1,802 (25%) enrolled.
Bunthi 2019	Thailand	9.1	No	No	To describe methodology of severe and fatal pneumonia (SevPn) surveillance system and provide preliminary results on the surveillance objectives over a five-year period.	Across all levels	Dec 2010 - Dec 2015	People of all ages with severe pneumonia across 30 different health care sites in Thailand	Included: Met case definition. Cases with CAP who died without being ventilated and within 48 hours of admission. Excluded: Illness onset >=2 weeks pre-identification or hospitalised within prior week. Hospital-acquired pneumonia, chronic pulmonary disease, swallowing dysfunction, or neurological condition causing inability to perform daily activities.	Severe pneumonia case defined as CAP with radiographic findings consistent with pneumonia, no aetiology identified by laboratory testing and in patient aged >=2 months, requiring ventilator support and hospitalized <=48 hours.	Total 972,<br>< 5 years 600 (62%)
AFRO WHO REGION											
LOW-INCOME COUNTRIES											
O'Callaghan-Gordo 2011	Mozambique	73.2	No	No	To present data on the epidemiology of several respiratory viruses associated with clinical pneumonia. The role of concomitant bacterial and malaria infections as well as HIV status on viral pneumonia cases was also evaluated.	District/ Secondary Hospital	Sep 2006 - Sep 2007	Children <5 years admitted to Manhica District Hospital (MDH) were enrolled. MDH is the referral health facility for Manhica District, a rural area of Maputo Province in Southern Mozambique.	Included: Met study case definition; Excluded: No written informed consent	**Clinical severe pneumonia** Cough &/or increased respiratory rate (WHO definition) & >=1: indrawing, nasal flaring, grunting or crackles on examination.	2951 <5yo admitted; 28% (835/2951) enrolled;<br><br>NPA from 97% (807/835)

Razanajatovo 2018	Madagascar	53.6	Yes - 2012	Yes - 2008	To identify aetiologies of SARI and to assess clinical features of hospitalisation linked to SARI in 2 hospitals during 3 years of surveillance.	Other: One secondary and one tertiary hospital	Nov 2010 – July 2013	Two hospital sites: Soavinandriana CENHOSOA national referral hospital of Antananarivo, capital of Madagascar and secondary level public hospital of Moramanga which includes semiurban and rural areas. Hospitalised patients of all ages.	Included: Met SARI case definition. Onset of illness <7 days before hospitalisation. Excluded:No written informed consent	SARI WHO case definition ##	876 all ages: < 5yrs 710 (81.1%); 5-14yrs 37 (4.2%).
LOWER-MIDDLE INCOME COUNTRIES											
Berkley 2010	Kenya	41.1	No	Yes - 2002	To determine viral aetiology, incidence, and clinical features among infants and children meeting WHO clinical criteria for severe pneumonia or very severe pneumonia admitted to a rural Kenyan district hospital.	District/ Secondary Hospital	Jan 2007 – Dec 2007	Children aged 1 day - 12 years residing in census area and admitted to the paediatric wards. Recruited 2 further sets of children (mild URTI and well) resident in the census area by convenience sampling for comparison.	Included: Met WHO criteria for the clinical syndromes of severe pneumonia	WHO severe* or very severe# case definition.<br><br>Mild URTI = cough, runny or blocked nose, sore throat, or sneezing.<br><br>Well = no URTI/LRTI & attending Hospital for routine immunisation.	LRTI=759/922 eligible (82%),<br><br>Outpatient URTI group = 96<br><br>Well control participants = 57
UPPER-MIDDLE INCOME COUNTRIES											
Cohen 2015	South Africa	33.8	Yes -2009	Yes - 2000	To describe the incidence, aetiology and factors associated with death among HIV-infected and HIV-uninfected children aged <5 years hospitalised with LRTI in South Africa from 2009 through 2012.	Other: 3 district and 1 tertiary hospital	Feb 2009 – Dec 2012	Active, prospective, hospital-based surveillance was implemented in 3 of the 9 provinces of South Africa in 2009. Additional surveillance site introduced in 2010. All patients <5 years of age admitted from Monday through Friday were eligible.	Included: Met study case definition; Excluded: No written informed consent	SARI WHO case definition ##	13815 screened, 8723 (63%) enrolled.<br>All 8723 had NPA and blood for lytA; 2893/8723 (33%) had sterile site specimens for bacterial culture
PAHO WHO REGION											
UPPER-MIDDLE INCOME COUNTRIES											

Verani 2013	Guatemala	26.2	No	Yes - 2005	To measure the burden of hospitalised ARI in the catchment area and characterise ARI aetiology.	Other:	Nov 2007 – Dec 2011	Ongoing daily surveillance for all ages conducted at primary public hospital in Santa Rosa & Quetzaltenango departments and both serve as the regional reference hospitals.	Included: Met study case definition; Excluded: No written informed consent	ALRI case definition **	4276 met case definition, 3,964 (93%) enrolled

*WHO case definition of severe pneumonia was used in this study. This definition includes the presence of chest in drawing and/or rapid breathing (i.e., children under 2months of age >60breaths/min; children between 2and 12 months of age >50breaths/min; children over12 months-5 years of age >40breaths/min) with or without fever

#WHO very severe pneumonia=cough or difficult breathing plus >=1 of hypoxia, defined as an oxygen saturation 90% by fingertip pulse oximetry, inability to drink or breastfeed, inability to sit, or impaired consciousness at admission.

**Acute lower respiratory infection (ALRI) case = evidence of both active infection (at least one of: reported fever, reported chills, measured temperature >38.2◦C or <35◦C, or an abnormal white blood cell count or differential) and lower respiratory tract disease (at least one of: abnormal breath sounds, documented tachypnoea, cough, sputum production, or dyspnoea) in a hospitalized patient. Verani: For children <2 years: Not eating, drinking or breastfeeding, pausing repeatedly while drinking or breastfeeding, chest indrawing, nasal flaring, noisy breathing.

##Cohen: SARI case definition = case of hospitalized-LRTI was defined as a hospitalized child with <7 days symptom duration meeting age-appropriate clinical case definitions (aged 2 days through <3 months of age with physician diagnosis of LRTI, including bronchitis, bronchiolitis, pneumonia and/or pleural effusion).

Razanajatovo: SARI case definition = fever (T>=38◦C) or history of fever and cough that required hospitalization. For children <5 years, eligibility criteria were suspected sepsis or SARI diagnosed by physician including bronchiolitis, pneumonia, bronchitis, pleural effusion, and cough or difficult breathing.

Table S5: Characteristics of cohort studies

Lead Author and publication date	Country	WHO U5MR/ 1000 live births)	PCV introduce d at time of study	Hib introduced at time of study	Aim of study	Healthcare level	Study period	Population description	Inclusion / Exclusion criteria	Case definition for LRTI group	Total number of participants in LRTI group
AFRO WHO REGION											
LOW- INCOME COUNTRIES											
Lanaspa 2015	Mozambique	73.2	No	No	To determine PCP prevalence among children admitted with severe clinical pneumonia to a district hospital in southern Mozambique and to describe clinical, laboratory, microbiologic and radiologic features.	District/ Secondary Hospital	Sep 2006 – Sep 2007	Children 0 - 5 years old admitted to Mahica District Hospital, referral health facility for rural area in Mozambique with WHO defined severe pneumonia.	Included: WHO defined severe pneumonia; Excluded: Clinical signs >2 weeks and household contacts of know tuberculosis cases.	WHO severe pneumonia	2943 admitted children, 834 severe pneumonia and provided consent.
Annamalay 2016	Mozambique	73.2	No	Yes - 2009	To describe the prevalence of human rhinovirus species in children hospitalised with pneumonia in Manhica, Mozambique and associations with demographic, clinical and laboratory features.	District/ Secondary Hospital	Sep 2010 – April 2013	Children aged 0-10 years with ALRI symptoms in the Manhica district, Mozambique	Included: Children with WHO defined ALRI; Excluded: Prior study enrolment, antibiotics or antimalarial drugs during preceding 2 weeks, history of cough for >2 weeks, active TB or history of direct contact with TB case, SpO2<85% on examination.	Fever (or a history of fever (>37.5°C) in the preceding 24 hours and clinical pneumonia according to the WHO definition.	277 cases<br>38 children were HIV-infected, 31 children were co-infected with malaria
LOWER-MIDDLE INCOME COUNTRIES											
Assane 2018	Senegal	43.6	Yes - 2014	Yes - 2005	To investigate viral and bacterial infections associated with ARIs in children under 5 years in Senegal.	Tertiary/ Teaching Hospital	Jan 2015 – Jan 2016	Conducted at Albert Royer Paediatric hospital, the Roi Baudouin Hospital, and the Abass Ndao Hospital, all located in Dakar, Senegal. Children aged 0-5 years presenting to hospital with ARI were screened.	Included: Children aged under 5 years attending hospital with an upper or lower airway infection	Acute respiratory infection – both upper and lower airway infection	162; 42 cases defined as pneumonia with positive pathogen aetiology result. 42/162 = 25.9%.
SEARO WHO REGION											

LOWER-MIDDLE INCOME COUNTRIES											
Jullien 2020	Bhutan	29.7	No	Yes - 2012	To describe epidemiology, aetiology, clinical and radiological presentation of WHO-defined pneumonia among children 2-59 months admitted to Jigme Dorji Wangchuck National Referral Hospital in Thimphu.	Tertiary/ Teaching Hospital	July 2017 – June 2018	Prospective hospital-based study conducted at the Jigme Dorji Wangchuck National Referral Hospital in Thimphu. Children aged 2-59 months hospitalised with WHO-defined pneumonia.	Included: Met WHO pneumonia case definition, Written consent.<br>Excluded: Children admitted in the preceding seven days or evidence of a foreign body in the respiratory tract.	WHO 2013 definitions for pneumonia, severe pneumonia or severe respiratory distress.	1062 admitted, 286 (27%) with respiratory symptoms and 189 (66%) enrolled.<br>BC 148/189 (78.3%); NPW 129/189 (68.3%)
Mathew 2015	India	36.6	No	Yes - 2013	To determine microbiologic aetiology of CAP in a cohort of Indian children using multiple biological specimens and the relationship between aetiology and pneumonia severity (CAPES Study).	Tertiary/ Teaching Hospital	April 2011 – Mar 2013	Advanced Pediatrics Centre at PGIMER Chandigarh. Children aged 1 month to 12 years, with WHO IMCI CAP, identified through active (household visits) and passive (outpatient and ED) surveillance.	Included: Met study case definition.<br>Excluded: Children with duration of illness >7 days; received antibiotics for >24 hours at presentation; previous hospitalization within the preceding 30 days; wheeze that disappeared after single dose of bronchodilator	Pneumonia defined as per the WHO IMCI definition for CAP.	36676 screened, 3144 eligible, 2345 enrolled. Blood culture 2285 (97.4%), NPA 2323 (99.1%).
UPPER-MIDDLE INCOME COUNTRIES											
Aman 2020	Indonesia	25	No	Yes - 2013	Identify pathogens associated with SARI, compare with hospital assessed aetiology, describe characteristics, determine performance of SARI criteria in identification of influenza viruses.	Tertiary/ Teaching Hospital	2013-2016	Patients with SARI within the AFIRE cohort: Aged >1-years old presenting to one of 8 Indonesia tertiary hospitals with fever and SARI.	Included: Met case definition<br>AFIRE inclusion criteria: fever, onset < 10 days, requiring hospitalisation<br>Excluded if no consent.	WHO defined SARI*	1464 enrolled, 607 (42%) aged 1-18 years; total 420 SARI<br>(244 resp, 176 not resp disease)
WPRO WHO REGION											
UPPER-MIDDLE INCOME COUNTRIES											
Nathan 2020	Malaysia	7.8	No	Yes - 2004	To determine aetiology of pneumonia, factors associated with bacterial pneumonia and association between co-infections and severity of	Tertiary/ Teaching Hospital	Oct 2014 - Oct 2016	University Malaya Medical Centre in Kuala Lumpur, Malaysia. Patients recruited via convenient	Included: Met study case definition; Excluded: (1) Children who had doctor diagnosed asthma or recurrent wheezing (more than 2 episodes), (2)	WHO very severe pneumonia definition	307 enrolled and 300 tested (7 samples lost)

					disease, in children admitted with severe pneumonia.			sampling from the paediatric wards. All children 1-month to 5-years-old admitted with pneumonia who met study criteria.	Refused blood taking, (3) Symptoms > 7 days, (4) Unable to come for follow-up, (5) Chronic disease, (6) No chest radiograph performed.		
Zhang 2011	China	8.6	No	No	To determine the aetiology of hospitalised community acquired pneumonia in children in China.	Tertiary/ Teaching Hospital	Oct 2004 - Oct 2005	Children 2 months to 14 years with CAP admitted to Department of Paediatrics, Second Hospital of Lanzhou University. Hospital serves urban and rural community with high levels of air pollution.	Included: Children 2 months to 14 years, Admitted directly from community with CAP, Meeting CAP definition.<br><br>Excluded: Children diagnosed with TB, Lack of parental consent, No convalescent serum sample.	CAP defined as the simultaneous finding of fever (37.5°C) and/or respiratory symptoms (cough, expectoration) and infiltrates compatible with pneumonia on CXR.	884 (29%) children hospitalised with CAP were eligible, 821 (93%) of the children admitted with CAP completed the study.<br>Return for follow-up 3-4 weeks after discharge for serology and follow-up CXR.

*Severe acute respiratory infection (SARI) per the WHO definition (a) an acute respiratory illness, (b) history of fever or measured fever of ≥38°C, (c) cough, (d) onset within the past 10 days, and (e) requiring hospitalization

Acute respiratory illness (ARI) defined as: any sign or symptom related to the respiratory tract including coryza, nasal congestion, sore throat, haemoptysis or dyspnoea.

Pneumonia defined as per the WHO IMCI definition as cough or difficult breathing associated with age-defined tachypnoea. Tachypnoea was defined as respiratory rate >60/min for infants <2 months; >50/min for infants 2-12 months; >40/min for children >12-60 months; and >30/min for children >60-144 months.

WHO definitions 2013: (1) Pneumonia: History of cough or reported breathing difficulty, AND Increased respiratory rate according to age OR chest indrawing. (2) Severe pneumonia: History of cough or reported breathing difficulty AND at least one of the following: Oxygen saturation <90% or central cyanosis, Severe respiratory distress (e.g. grunting, very severe chest indrawing), Signs of pneumonia with a general danger sign: inability to breastfeed or drink, lethargy or reduced level of consciousness, convulsions.

Very severe pneumonia definition: history of cough and/or shortness of breath with examination findings of age-defined tachypnoea and one of the following: recessions, saturation <92% on air, poor feeding or lethargy.

Table S6: Characteristics of cross-sectional studies

Lead Author and publication date	Country	WHO U5MR/ 1000 live births	PCV introduced at time of study	Hib introduced at time of study	Aim of study	Healthcare level	Study period	Population description	Inclusion/ Exclusion criteria	Case definition for LRTI group	Total # participants LRTI group & proportion tested
AFRO WHO REGION											
LOWER-MIDDLE INCOME COUNTRIES											
Kwofie 2012	Ghana	47.9	No	Yes - 2002	To determine the burden of respiratory viruses among children hospitalized at the Komfo Anokye Teaching Hospital for acute lower respiratory illness.	Tertiary/ Teaching Hospital	Jan 2008 – Dec 2008	Children ≤5 years hospitalized for ARTI at the Komfo Anokye Teaching Hospital, Ashanti Region, Kumasi, Ghana.<br>2-3 patients/week, until maximum of 11 patients per month.	Included: (1) Met case definition for WHO severe or very severe pneumonia, (2) Written consent;<br>Excluded: (1) If both blood and NPS samples could not be obtained.	# WHO-IMCI severe pneumonia or very severe pneumonia	128
PAHO WHO REGION											
UPPER-MIDDLE INCOME COUNTRIES											
Nascimento-Carvalho 2016	Brazil	14.4	No	Yes - 2000	To assess whether there was difference in the frequency of bacterial and viral infections among children with CAP in distinct severity subgroups.	Tertiary/ Teaching Hospital	Sep 2003 – May 2005	All children 2-59 months meeting the study case definition of CAP seen at the Emergency Room of the Federal University of Bahia Hospital, in Salvador, North-eastern Brazil.	Included: Met CAP diagnosis, Previously healthy children, Written consent;<br><br>Excluded: Chronic lung disease except asthma, underlying comorbidities, Other concurrent infections, Suspected/diagnosed immunodeficiency, Transferred from other health-care units.	CAP diagnosis: respiratory complaints, fever or difficulty breathing plus detection of pulmonary infiltrate or pleural effusion on CXR. Only children with tachypnoea or chest indrawing included in analysis.	322 screened, 181 (56.2 %) enrolled.
WPRO WHO REGION											
UPPER-MIDDLE INCOME COUNTRIES											

Xu 2018	China	8.6	No	No	To describe the frequency and types of different infectious aetiologies among hospitalized children and adults in Tianjin during 2015 and 2016.	Tertiary/ Teaching Hospital	Jan 2015 – Dec 2016	Enrolled hospitalised patients of all ages admitted in the respiratory medicine ward, paediatric medicine ward, or ICU in a tertiary hospital (Third Central Hospital, Tianjin) who met the SARI case definition.	**Included:** Patients hospitalised in the relevant wards at the study site; Met SARI criteria;<br><br>**Excluded:** No consent by participant/parent	##Revised WHO SARI case definition (2014)	7,156 hospitalised, 2,290 (32%) met case definition - 618 aged <15 years.<br>Specimens:<br>All ages 585,<br><5 years 96,<br>5-14 years 62.
Zhong 2019	China	8.6	No	No	To investigate the viral and atypical bacterial aetiology of LRTI in children and compare the clinical characteristics of HPIV single infection with those of co-infection.	Tertiary/ Teaching Hospital	Dec 2013 – June 2015	Children 1-71 months hospitalised with LRTI at conducted prospectively at the Pediatric Respiratory Department of Yuying Children's Hospital affiliated to Wenzhou Medical University, Zhejiang, China.	**Included:** Children hospitalised during study period; within 7 days of the disease at admission and a diagnosis of LRTI;<br>**Excluded**: PCR failure or insufficient sample; incomplete clinical data.	$ Zhu Futang Practice of Pediatrics LRTI definition including bronchitis, bronchiolitis, and pneumonia.	1367 children screened and 1335 (98%) enrolled.

*WHO severe pneumonia definition (modified) = cough or difficulty in breathing, plus any of the following: lower chest wall indrawing, nasal flaring, or oxygen saturation <90% on pulse oximetry.

**WHO radiological pneumonia = endpoint consolidation or pleural effusion.

WHO-IMCI severe pneumonia or very severe pneumonia: Severe pneumonia defined as history of cough and/ or difficult breathing of less than 3 weeks duration, with lower chest wall recession. Severe pneumonia in addition to cyanosis and/or inability to feed or drink was classified as very severe pneumonia.

##Revised WHO SARI case definition (2014) including fever (axillary temperature >=38°C) and cough with onset within 10 days.

$ Zhu Futang Practice of Pediatrics LRTI definition: clinical symptoms, including severe cough, fever, tachypnoea, wheezing, and respiratory distress signs such as nasal flaring, retraction, cyanosis, and abnormal auscultatory findings (wheezing and crackles), or radiologic evidence indicative of an LRTI. Clinical syndromes of bronchitis, bronchiolitis, and pneumonia were included in the LRTI category.

Bronchitis was diagnosed based on the clinical manifestations including severe cough with or without fever, symmetrical breath sounds without permanent rales on auscultation, and increased bronchovascular shadows in CXR examination.

Bronchiolitis in patients aged <24 months with lower respiratory symptoms of wheezing, tachypnoea, and signs of respiratory distress such as nasal flaring, intercostal/subcostal retractions, and central cyanosis.

Pneumonia based on clinical findings, including fever, tachypnoea, and respiratory distress, with the presence of focal or diffuse crackles, decreased vesicular sounds, and radiographic findings such as patchy and macular shadows and/or atelectasis, and/or air bronchograms.

Table S7: Characteristics of other studies

Lead Author and publication date	Country	WHO U5MR/ 1000 live births	PCV introduced at time of study	Hib introduced at time of study	Aim of study	Study design	Healthcare level	Study period	Population description	Inclusion/Exclusion criteria	Case definition for LRTI group	Total # of participants in LRTI group
WPRO WHO REGION												
LOWER-MIDDLE INCOME COUNTRIES												
Dembele 2019	Philippines	28.4	Yes - 2013	Yes - 2011	To define the aetiological, demographic and environmental factors and clinical findings associated with mortality due to childhood pneumonia among hospitalised children in the Philippines.	Case series	3 Tertiary and 1 secondary hospital	Jun 2008 – Mar 2016	Children 8 days to <5 years with pneumonia hospitalised in four hospitals (urban and rural) in the Philippines. Study periods varied among the hospitals.	**Included:** Met case definition. **Excluded:** Admitted for another illness, developed pneumonia symptoms during hospitalisation, admitted to another hospital within last 3 days, prior hospital admission without 1-week symptom-free period when readmitted.	Severe or very severe pneumonia according to 2005 WHO IMCI algorithm	5128 enrolled, 5054 (98.6%) with outcome data - 5054 had NPS and 3018 (60%) had blood cultures
Guerrier 2013	Cambodia	28	Yes - 2015	Yes - 2010	To describe viral aetiologies, clinical and epidemiologic characteristics among children <5 years of age admitted for LRTI in 2 hospitals in Cambodia.	Prospective study	District/ Secondary Hospital	Apr 2007 – Feb 2010	All children <5 years of age admitted with LRTI to two rural secondary provincial hospitals located in Takeo Province and Kampong Cham Province, Cambodia.	**Included:** Met case LRTI criteria, Onset of illness <14 days pre-hospitalisation; **Excluded:** Discharged from hospital previous 21 days, Newborns who never left hospital, Uncomplicated URTI, Proven or suspected TB or HIV or non-infectious respiratory symptoms	LRTI diagnosed on clinical grounds with symptoms of cough or respiratory distress associated with tachypnoea, with or without fever.	1250 screened, 1006 enrolled - pneumonia cases 423 (42%), bronchiolitis 428 (42%), ULRTI 155 (15%).
Huong 2014	Vietnam	20.7	No	Yes - 2010	To determine the prevalence of and risk factors for severe CAP caused by *M. pneumoniae, C. pneumoniae* and *L. pneumophila* in hospitalised children with CAP, 1-15 years old in Vietnam.	Prospective hospital-based study	Tertiary/ Teaching Hospital	Jul 2010 – Mar 2012	Children aged 1 to 15 years with CAP recruited at the National Hospital of Pediatrics, Hanoi.	**Included:** Met study definition, Written consent	# Atypical pathogen-positive CAP (ApCAP) case definition ##Severe pneumonia (PIDS and IDSA)	722
UPPER-MIDDLE INCOME COUNTRIES												

Chen 2013	China	8.6	No	No	To investigate the epidemiology of *M. pneumoniae* & C. *pneumoniae* infections among children hospitalised with acute respiratory infections in Suzhou, China, & association between incidence rates and climatic conditions.	Prevalence study	Tertiary/ Teaching Hospital	Jan 2006 – Dec 2006	Consecutive hospitalised children 1 month - 13 years with ARTI in Soochow University Children's Hospital Suzhou, Jiangsu Province, China.	Included: Met study case definition.<br>Excluded: Children with chronic lung disease, immunodeficiency, congenital heart disease, or bronchopulmonary dysplasia	**ARI:** Fever >3d, severe symptoms of cough, wheeze, tachypnoea, chest retractions.<br>**URTI:** nasal obstruction/ discharge, fever, or sore throat.<br>**LRTI:** wheeze, chest retractions, tachypnoea, auscultatory findings, CXR evidence.	1598 children with ARTI
Oumei 2018	China	8.6	No	No	To investigate the role of eight common viral respiratory pathogens and *M. pneumoniae* in the aetiology of childhood CAP and the associated epidemiologic characteristics of these pathogens.	Prospective observational study	Tertiary/ Teaching Hospital	Jan 2015 – Dec 2015	Conducted at 10 hospital sites in nine cities. 1500 hospitalized children 6 months to 14 years. (150 cases/each hospital) with CAP were enrolled in the study. Clinicians screened and enrolled eligible children 8 h per day, 7 days per week.	Included: Met case definition.<br>Excluded: No written informed consent	Pneumonia cases using Child CAP Guidelines of the Chinese Medicine Association: recent fever (>37.5°C), cough and/or dyspnoea, tachypnoea, and sputum; fixed moderate or fine rales or dry rales during inspiration on lung auscultation; and radiological confirmation of pneumonia (presence of consolidation), other infiltrate or pleural effusion.	1500
Jiang 2017	China	8.6	No	No	To investigate the aetiologic spectrum and occurrence of co-infections in children with CAP.	Prospective observational study	Tertiary/Teaching Hospital	Jan 2015 – Dec 2015	Consecutive children 1 month to 14 years old with CAP admitted to Children's Hospital of Soochow University, SE Jiangsu Province of East China.	Included: Met study case definition, Written informed consent.<br>Excluded: Preterm birth <=34 weeks GA, Hospitalisation in last 4 weeks, Pre-existing conditions: Immunodeficiency, history of chronic lung disease, or congenital heart disease.	Preceding fever (defined as body temperature >=38°C), and clinical (chest retractions, tachypnoea, nasal flaring, hypoxia, or abnormal auscultatory findings) and radiologic evidence of CAP.	846 enrolled and sampled.
EURO WHO REGION												
UPPER-MIDDLE INCOME COUNTRIES												

Aykac 2018	Turkey	10.6	Yes - 2011	Yes - 2007	To determine the prevalence and seasonal distribution of viruses in childhood respiratory infections in a tertiary hospital in Turkey	Prevalence study	Tertiary/ Teaching Hospital	Dec 2006 – Dec 2015	Children 0-18 years presenting to outpatient/inpatient departments of a Turkish hospital (Hacettepe University Medical Faculty Ihsan Dogramacı's Child Hospital) over a 10-year period with ARI symptoms	Included: Met study case definition.<br>Excluded: Duplicate specimens when same results, Samples that did not have test results because of failure of PCR or insufficient sampling/missing details.	LRTI = one or more of hypoxia <95%, age-related tachypnoea, signs of respiratory distress or abnormal breath sounds.<br>URTI = cough, nasal congestion, rhinorrhoea, and/or fever without associated wheezing or dyspnoea	All in- and out-patients = 1240 LRTI = 264<br>URTI = 64.
PAHO WHO REGION												
UPPER-MIDDLE INCOME COUNTRIES												
Jonnalagadda 2017	Ecuador	14.2	Yes - 2010	Yes - 2003	To describe the epidemiological characteristics and aetiology of pneumonia in a trial investigating zinc as an adjunct treatment for severe pneumonia.	Observational study within context of RCT	Tertiary/ Teaching Hospital	Feb 2008 – April 2010	Children 2-59m with severe pneumonia (RCT designed to measure efficacy of daily zinc on duration of pneumonia symptoms). Enrolled at main paediatric referral hospital, Baca Ortiz Hospital in Quito, Ecuador.	Included: Met study eligibility criteria, Written informed consent.<br>Excluded: Marasmus or kwashiorkor, measles, pneumonia due to foreign body aspiration, hepatic or renal disease, sepsis, congenital abnormalities, very severe or complicated pneumonia.	Cough and/or difficulty breathing, tachypnoea, hypoxemia (SpO2 <90%) and at least one of the following: rales, wheezing, diminished breath sounds, bronchial breath sounds or pleural rub.	450 enrolled, 406 (90.2%) had blood or NPS
SEARO WHO REGION												
LOWER-MIDDLE INCOME COUNTRIES												
Chisti 2014	Bangladesh	30.2	No	Yes - 2009	To determine the prevalence of bacteraemia and TB in severely malnourished children with pneumonia, compare Xpert MTB/RIF with culture in sputum samples, and compare TB diagnosis from induced sputum versus gastric lavage.	Prospective descriptive study	Tertiary/ Teaching Hospital	Apr 2011 – June 2012	Severely malnourished children 0-5 years, admitted to inpatient wards of the Dhaka Hospital with respiratory symptoms (cough and/or respiratory distress) and radiological pneumonia	Included: Only severely malnourished children with pneumonia	Respiratory symptoms (cough and/or respiratory distress) and radiological pneumonia<br><br>*WHO radiological classification of pneumonia	405
AFRO WHO REGION												
LOW-INCOME COUNTRIES												

Graham 2011	Malawi	49.7	No	Yes - 2002	To describe the causes of severe and very severe pneumonia in Malawian children and infants >2 months of age and to examine the impact of HIV infection on causes and outcome.	Prospective descriptive study	Tertiary/ Teaching Hospital	Jul 2005 – Nov 2006	Children 2mos-14 yrs admitted to Queen Elizabeth Central Hospital, Blantyre, with clinical diagnosis of severe pneumonia. Monday to Thursday each week.	Included: (1) Met case LRTI criteria, (2) Written informed consent; Excluded: (1) Children with severe malnutrition	WHO-defined severe and very severe pneumonia	350 screened and 327 enrolled
UPPER-MIDDLE INCOME COUNTRIES												
Morrow 2014	South Africa	33.8	No	Yes - 2000	To describe the incidence, clinical features and outcome of PCP in children when diagnosed with PCR.	Prospective observational study	Tertiary/ Teaching Hospital	Nov 2006 – August 2008	Consecutive children hospitalised for acute hypoxic pneumonia at Red Cross War Memorial Children's Hospital in Cape Town, South Africa. Median age 3.2 (2.1-4.6) months	Included: Met case definition, Written informed consent; Excluded: Received treatment for PCP in the preceding 2 weeks, if they had been on PCP therapy for the acute illness for more than 48 hours or if informed consent was not obtainable.	Acute onset of respiratory illness requiring hospitalization, defined as age specific tachypnoea, hypoxia (SpO2<90%), and diffuse lung disease not associated with wheezing	202 sampled and tested
MIXED WHO REGIONS AND INCOME CLASSIFICATIONS												
GABRIEL NETWORK												
Dananche 2018	**WPRO:** Cambodia (KH), China (CN), Mongolia (MN); **SEARO:** India (IN), **AFRO:** Madagascar (MG), Mali (ML); **PAHO:** Haiti (HT), Paraguay (PY).	**LIC:** HT 64.8, MG 53.6, ML 97.8; **LMIC:** KH 28, IN 36.6, MN 16.3; **UMIC** CN 8.6, PY 20.2.	**KH** No; **CN** No; **HT** No; **IN** No; **MG** Yes - 2012; **ML** Yes - 2011; **MN** No; **PY** Yes - 2012	**KH** Yes - 2010; **CN** No; **HT** Yes - 2013; **IN** Yes - 2013; **MG** Yes - 2008; **ML** Yes - 2005; **MN** Yes - 2005; **PY** Yes - 2003	To estimate the age- and country-stratified proportion of influenza-related pneumonia. Secondary objectives were the description of clinical and microbiological characteristics of particularly bacterial co-infections in influenza-related pneumonia cases.	Secondary analysis of cases from CCS	Mixture of district and tertiary hospitals	Dec 2009 – Jan 2014	Pneumonia cases 2-59 months enrolled in a large, multicentre, prospective case-control study in nine urban and rural settings from eight countries. Enrolment of at least 100 hospitalised cases from each site over a 12-month period.	Included: 1) Clinical features of pneumonia, 2) WHO radiological pneumonia on CXR, 3) Informed consent signed, 4) Symptoms appearing within the last 14 days;<br>Excluded: 1) Presence of wheezing at auscultation, or 2) minors whose parents or legal guardian declined to sign informed consent statement.	*WHO radiological classification of pneumonia<br><br>**WHO clinical pneumonia	888 enrolled (Haiti 101, Mongolia 108, India 167, Mali 118, China 39, Paraguay 99, Cambodia 176, Madagascar 80)

*WHO radiological classification of pneumonia: presence of end-point consolidation or other (non-end-point) infiltrate in lungs

**WHO pneumonia: Cough and/or dyspnoea, and tachypnoea (in children between 2 and 12 months of age: breathing rate >=50 cycles per minute; in children between 12 months and 59 months of age: breathing rate >=40 cycles per minute)

Atypical pathogen-positive CAP (ApCAP) case definition: 1) patients with clinical symptoms of pneumonia, confirmed by radiography and 2) presence of MP and/or CP and/or LP, detected in BAL, identified by multiplex PCR and ELISA based specific IgM antibodies against MP, or CP, or LP in one of paired sera.

Criteria for classification of severe pneumonia (Pediatric Infectious Diseases Society and Infectious Diseases Society of America): Major criteria (>=1 major criteria): invasive mechanical ventilation; fluid refractory shock; hypoxemia requiring fraction of inspired oxygen (FiO2) greater than inspired concentration or flow feasible in general care area;; Minor criteria (>=2 minor criteria): respiratory rate higher than WHO classification for age; apnoea; increased laboured breathing; the ratio between partial pressure of arterial oxygen (PaO2) and fraction of inspired oxygen (FiO2) < 250; multilobar infiltrates (>=2 lobes); Pediatric Early Warning Signs (PEWS) score > 6; altered mental status; hypotension; presence of effusion.
